# Cultural Differences in Strength of Conformity Explained Through Pathogen Stress: A Statistical Test Using Hierarchical Bayesian Estimation

**DOI:** 10.3389/fpsyg.2018.01921

**Published:** 2018-10-11

**Authors:** Yutaka Horita, Masanori Takezawa

**Affiliations:** ^1^Department of Psychology, Teikyo University, Tokyo, Japan; ^2^Department of Behavioral Science, Hokkaido University, Sapporo, Japan

**Keywords:** collectivism, conformity, pathogen stress, institution, Galton’s problem, hierarchical model, Bayesian estimation

## Abstract

The severity of the environment has been found to have played a selective pressure in the development of human behavior and psychology, and the historical prevalence of pathogens relate to cultural differences in group-oriented psychological mechanisms, such as collectivism and conformity to the in-group. However, previous studies have also proposed that the effectiveness of institutions, rather than pathogen stress, can account for regional variation in group-oriented psychological mechanisms. Moreover, previous studies using nations as units of analysis may have suffered from a problem of statistical non-independence, namely, Galton’s problem. The present study tested whether or not regional variation in pathogen stress, rather than government effectiveness, affected collectivism and conformity to social norms by adjusting the effect of global regions using hierarchical Bayesian estimation. We found that the overall effect of pathogen stress remained significant in only one out of the four indices of the regional variability of conformity, and the effects of the government effectiveness also disappeared. Instead, we found that significant effects of both pathogen stress and government effectiveness in specific regions of the world, but these effects were not stable across the measurements. These results indicate that both the effects of pathogen stress and government effectiveness need further reevaluation.

## Introduction

Humans live in large-scale groups, and each group forms different cultural values. Studies in cross-cultural psychology have reported cultural differences in psychological mechanisms (Triandis, 1995; Hofstede, 2001; Gelfand et al., 2004). Recently, the reasons for these cross-cultural differences in values have been explained as adaptation to ecological threats.

A group of cultural and evolutionary psychologists have argued that ecological threats such as pathogen stress have played a significant role in adapting human psychology to local ecology. A series of studies based on the “behavioral immune system hypothesis” (Schaller, 2011; Murray and Schaller, 2016) has argued that the prevalence of infectious diseases was one of the important ecological variables to explain the cultural differences in human collectivistic psychology and behaviors (Fincher et al., 2008; Schaller and Murray, 2008; Thornhill et al., 2009, 2010; Murray and Schaller, 2010, 2012; Murray et al., 2011, 2013; Schaller, 2011; Fincher and Thornhill, 2012; Van Leeuwen et al., 2012; Cashdan and Steele, 2013; Varnum, 2013; Murray, 2014; Tybur et al., 2016). Infectious diseases historically served as significant ecological hazards and imposed strong selection pressure on humans. Humans, along with other animals, are thought to have acquired functionally adaptive psychological or behavioral responses to the dangers posed by infectious diseases. In contexts characterized by a higher prevalence of infectious diseases, avoiding interactions with unknown out-group members might have been an adaptive response, since extroversion brings a greater risk of exposure to infections. In addition, conformity to social norms has also been argued to be beneficial in such contexts, because the traditional norms or rituals incorporate methods of avoiding infection, and individuals gain significantly more social support from in-group members by adhering to these norms (Schaller, 2011; Murray and Schaller, 2016).

Cross-cultural studies have provided evidence supporting this hypothesis. Previous studies that conducted group-level analysis with countries as units of analysis have shown that the regional variation in the historical prevalence of pathogens was positively associated with regional variation in collectivism (Fincher et al., 2008; Murray and Schaller, 2010; Thornhill et al., 2010; Cashdan and Steele, 2013). Although collectivism is a broad and multifaceted concept, researchers have further found positive relationships with other, more concrete variables, including the diversity of extraversion personality (Schaller and Murray, 2008), tightness of social norms (Gelfand et al., 2011), strength of family ties (Fincher and Thornhill, 2012), group-oriented moral concerns (Van Leeuwen et al., 2012), individuals’ authoritarian personality (Murray et al., 2013), adherence to traditional norms (Tybur et al., 2016), and conformity (Murray et al., 2011; Murray and Schaller, 2012; Varnum, 2013). Thus, these studies suggested that harsh natural environments functioned as selection pressure to enhance the psychology supporting in-group cooperation.

Other researchers have argued that the effect of pathogen stress has been confounded with the other social factors such as effectiveness of institutions (Hruschka and Henrich, 2013; Hruschka et al., 2014). Hruschka and Henrich (2013) found that the relationships between pathogen stress and the regional level of collectivism and in-group favoritism disappeared when regional differences in the effectiveness of modern institutions were controlled. These differences represent how effectively regions provide civil or public services, which were significantly (and negatively) associated with regional levels of collectivism and in-group favoritism (Hruschka and Henrich, 2013). Thus, such recent studies have raised doubts about the effects of pathogen stress on the development of group-oriented psychological mechanisms.

Nation-unit analyses always suffer from Galton’s problem (Mace and Pagel, 1994), in which units of analysis fail to ensure statistical independence. These previous studies employed regression analysis using countries (or regions) as units of analysis (Fincher et al., 2008; Thornhill et al., 2010; Murray et al., 2011; Hruschka and Henrich, 2013). However, countries are not statistically independent units; neighboring countries often share a common historical ancestry and thus tend to share similar cultural traits, not as a result of adaptation to a similar local ecology but simply as a result of shared historical backgrounds. Therefore, results of simple country-level analysis are not reliable, since they incorrectly assume the statistical independence of their units, which raises the risk of spurious associations. Currie and Mace (2012) found no consistent associations between pathogen prevalence and one form of human social behavior (religious participation) within each global region (i.e., Africa, Europe, East Eurasia, and so on).

Previous studies have accounted for this problem in several different ways. Some grouped countries into global regions related to shared historical and geographical backgrounds, computing correlations with these as units of analysis (Fincher et al., 2008; Murray et al., 2011; Fincher and Thornhill, 2012; Murray, 2014). However, this solution greatly reduces the number of data points to 6–10. Correlation coefficients calculated using such small samples are known to be unreliable and often exhibit inflated effect sizes (Yarkoni, 2009; Schönbrodt and Perugini, 2013). A common solution that is often employed in anthropology is to use the Standard Cross-Cultural Sample (SCCS; Murdock and White, 1969). This is a database that mainly consists of traditional smaller-scale societies sampled so that the cultural and historical relationships among the data points are minimized. Certain scholars have analyzed the SCCS to test the influence of pathogen prevalence on group-oriented psychology (Cashdan and Steele, 2013; Murray et al., 2013) and ritualized behavior (Murray et al., 2017). However, this solution is not feasible for the analysis of different types of data, in which nation states are a primary unit. More importantly, it has been recently shown that significant autocorrelations exist in the SCCS (Dow and Eff, 2008), which raises the question of the validity of the SCCS as a dataset for solving Galton’s problem (for other problems of the SCCS, see Mace and Holden, 2005; Nettle, 2009). Researchers seem to have agreed that the best approach to take is the phylogenetic one (Mace and Pagel, 1994; Mace and Holden, 2005; Beheim and Bell, 2011; Botero et al., 2014; Mathew and Perreault, 2015), in which the influences of shared cultural and historical ancestry are statistically controlled using phylogenetic information estimated from other sources, such as language family, historical records, and spatial proximity. However, the information necessary for applying the phylogenetic approach is not always available.

In this study, we propose another method for handling Galton’s problem for nation-level analysis: hierarchical Bayesian models, with global regions as random effects. Nettle (2009) reviewed anthropological studies on the influence of pathogen prevalence on human social systems, explicitly discussing the advantages of this approach. Galton’s problem can be rephrased to state that countries sharing a common historical and cultural ancestry should be correlated with each other and cannot be assumed to be statistically independent data points. This non-independence is often exhibited as spatial autocorrelation. With the use of global regions as random effects, autocorrelation can be appropriately controlled, even without knowing the exact processes that produce the correlations among neighboring countries (Nettle, 2009). The benefits of this approach have recently been demonstrated in the context of language evolution. Using cross-cultural, correlational analyses, Kashima and Kashima (1998) found that the presence of a linguistic phenomenon called the “pronoun-drop effect” is significantly correlated with the level of collectivism of the given culture. Lee (2017) conducted hierarchical Bayesian modeling to test the robustness of the finding, showing that the relationship between the phenomenon and individualism was not universal but observed only in a particular language family (the Indo-European language family).

We conducted hierarchical Bayesian modeling to estimate the effects of both regional pathogen stress and government effectiveness on variables related to group-oriented psychology. In our models, each country was nested within one of the six global regions, and the global regions were included as random effects. Under this framework, countries belonging to the same global region was assumed to share a common effect of the global region.

We conducted the analyses with two dependent variables: collectivism and conformity. Although collectivism is a broad and multifaceted concept, it appears to be a core dependent variable in the literature and its relationship with pathogen prevalence has been replicated in multiple studies. Collectivism includes both intergroup-oriented (e.g., xenophobic attitudes or barriers between social groups) and intragroup-oriented (e.g., conformity to social norms or in-group members) psychological constructs. Past studies have suggested that the regional strength of pathogen stress is more strongly related to the latter than to the former (Cashdan and Steele, 2013; Tybur et al., 2016). Thus, to rigorously examine the association of environmental severity with in-group oriented psychological constructs, we particularly focused on a concept of conformity, testing the hypothesis that pathogen stress affects conformity, even when the effect of institutions is controlled for.

## Materials and Methods

### Measures and Analysis

The basic unit in our analysis is geographical regions. In most cases, the unit is a country (e.g., France), but in some exceptional cases, culturally distinct regions within a country were used as basic units of analysis (e.g., Hong Kong). These treatments follow the previous studies (Fincher et al., 2008; Murray and Schaller, 2010; Thornhill et al., 2010; Murray et al., 2011; Hruschka and Henrich, 2013). We used standardized scores for all variables of analysis (except for categorical variables such as global regions).

### Independent Variables

#### Pathogen Stress

We used numerical estimates of the historical prevalence of pathogens provided by Murray and Schaller (2010), which reported the index of the historical prevalence of nine different infectious diseases (leishmania, schistosoma, trypanosome, leprosy, malaria, typhus, filarial, dengue, and tuberculosis) in the early 1900s within 230 geopolitical regions.

#### Government Effectiveness and GDP Per Capita

We used the World Bank’s measure of government effectiveness, which measures “*perceptions of the quality of public services*, *the quality of the civil service and the degree of its independence from political pressures*, *the quality of policy formulation and implementation*, *and the credibility of the government’s commitment to such policies* (World Bank, 2018).” The index includes the quality of bureaucracy, infrastructures such as roads and public transportation system, the quality of education system, and so on. The score was estimated ranging from −2.5 to 2.5 for each nation or region. Higher values mean better the quality of public services. We calculated the mean score from 1981 to 2008 and used this for analysis.

We also used GDP per capita as a factor representing the economic or institutional qualities of each region. We assessed GDP per capita from the World Bank’s data. We then averaged the scores of GDP per capita from 1981 to 2008. We also reported the results of an analysis using GDP per capita rather than government effectiveness as the independent variable.

### Dependent Variables

#### Individualism

We used Hofstede (2001) measures of individualism, which assess individualism and collectivism values, from more than 100,000 individuals worldwide. Higher scores indicate greater individualism, and lower scores indicate greater collectivism. This index is commonly used in studies that examine the relationship between pathogen stress and collectivism (Fincher et al., 2008; Murray and Schaller, 2010; Hruschka and Henrich, 2013). We retrieved updated scores for each country or region^1^. Other well-known measures exist that represent individualistic and collectivistic values in cross-cultural psychology (Suh et al., 1998; Gelfand et al., 2004). We also conducted that same analysis, only using each of them as a dependent variable, and report it in the **Supplementary Material** (see **Supplementary Analysis**). The results were nearly consistent with those done using Hofstede (2001) measure.

#### Regional Level of Conformity

We used cross-cultural survey data from the World Values Survey [WVS] (1981–2008), which are available electronically. This survey included questions about perceptions of human relationships. We used the following items, which represent sensitivity to being monitored by community members and positive views of obedience: *Conformity 1*: “One of my main goals in life has been to make my parents proud,” *Conformity 2*: “I make a lot of effort to live up to what friends expect,” *Conformity 3*: “Children should be encouraged to learn obedience at home,” and *Conformity 4*: “I seek to be myself rather than to follow others.” For *Conformity 1, 2*, and *4*, respondents answered degree of agreement (1: agree strongly, 2: agree, 3: disagree, 4: strongly disagree). For each region, we computed the percentage of respondents who had agreed with each statement (i.e., the percentage of respondents who had chosen 1: agree strongly or 2: agree). For *Conformity 3*, respondents were presented a list of qualities that children should learn at home (including obedience, unselfishness, religious faith, and so on), and were asked to choose qualities that they consider important. For each region, we computed the percentage of respondents who had chosen “obedience.” Previous studies have used other indicators to judge the strength of conformity. For example, Murray et al. (2011) used effect sizes for behavioral-conformity experiments, dispositional variability, and the proportion of the population who are left-handed. However, these sample sizes are too small (*n* = 17, 33, 20, respectively) to obtain converged results for complex models as in our hierarchical model. In order to keep as large sample sizes as possible, we used the above four items, which were asked from 1981 to 2009 in the WVS, and we could retrieve data from at least 50 regions. Previous studies also used two of the above four items (*Conformity 1* and *Conformity 3*), and reported that the percentage of the population who prioritize obedience and strength of family ties was strongly correlated with regional pathogen stress (Murray et al., 2011; Fincher and Thornhill, 2012). Fincher and Thornhill (2012) used an index of “strength of family ties” including the item *Conformity 1*. However, Hruschka and Henrich (2013) reported that government effectiveness, rather than pathogen stress, explained regional differences in the strength of family ties. In the present study, we investigated whether pathogen stress still had an effect on these items, which were expected to reflect conformity to community, even after controlling for both institutional factors and global regions.

### Global Region

To adjust for the effects of shared historical and cultural factors, we used six global regions defined by the World Bank based on geographic regions or income levels. We coded each country as follows: 1 = Sub-Saharan Africa, 2 = East Asia and Pacific, 3 = Europe and Central Asia, 4 = Latin America and Caribbean, 5 = Middle East and North Africa, and 6 = South Asia. Adopting a strategy from Hruschka and Henrich (2013), we classified some higher-income countries according to shared cultural backgrounds. For example, although the United States, Canada, New Zealand, and Australia are geographically distant from Europe, we grouped them with Europe and Central Asia. The number of countries or regions of each global region used for each analysis is shown in **Supplementary Table S1**.

### Models

We conducted three linear regression models to predict the level of conformity in each region from the regional prevalence of pathogens and government effectiveness. We did not assume a unique effect of global region in Model 1, while we did consider this in Models 2 and 3. To ensure the convergence of each model and avoid the problem of multicollinearity, we examined regression models including only two independent variables. In the following three models, we assumed that the dependent variable (standardized score), *y_i_*, obeys normal distribution [i.e., *y_i_* ∼ Normal(*ŷ_i_*, *σ_y_*)]. We implemented a normality assumption to keep the results comparable with those of previous studies, which used a standard linear model.

#### Model 1

First, we assumed Model 1 as follows:

(1) $$\begin{array}{l} {\hat{y}}_{i}=a_{0}+\text{GE}x_{1,i}+\text{PS}x_{2,i},\\ y_{i}∼\text{Normal}({\hat{y}}_{i},\sigma_{y}), \end{array}$$

where *i* represents the index of countries or regions, *x*_1,_*_i_* and *x*_2,_*_i_* represent the independent variables (government effectiveness and pathogen stress, respectively). *y_i_* represents the dependent variable. *a*_0_, GE, and PS are parameters. *a*_0_ represents an intercept. GE and PS represent the coefficient of the fixed effect of each independent variable. *ŷ_i_*, the expected value of the dependent variable, is predicted by government effectiveness and pathogen stress. We assumed that the value of the dependent variable, *y_i_*, obeys a normal distribution in which the mean equals *ŷ_i_* and the standard deviation equals *σ_y_*. We estimated the values of *a*_0_, GE, PS, and *σ_y_*. We set the uninformed priors (Box and Tiao, 1973) of *σ_y_* to the uniform distribution [0, ∞]. This model serves as a benchmark for replicating analyses from past studies in which countries were assumed to be statistically independent units and the effect of shared cultural backgrounds were not controlled for.

#### Model 2

We conducted two types of hierarchical linear regression analysis to adjust for the effects of global regions. Each country was nested within one of the six global regions, which served as a random effect in the model (i.e., unique effect by global regions) as well as a fixed effect (i.e., common effect across global regions). Model 2 is the random intercept model in which the global regions were used as a random effect affecting the intercept:

(2) $$\begin{array}{l} {\hat{y}}_{i}=a_{j}+\text{GE}x_{1,i}+\text{PS}x_{2,i},\\ a_{j}∼\text{Normal}(\mu_{a},\sigma_{a}),\\ y_{i}∼\text{Normal}({\hat{y}}_{i},\sigma_{y}), \end{array}$$

where *j* represents the global region (*j* ∈ [1,6]), and *a_j_* represents a random effect specific to the global region affecting the intercept. It is assumed that *a_j_* obeys the normal distribution [i.e., *a_j_* ∼ Normal(μ*_a_*, *σ_a_*)]. *a_j_* is sampled from the normal distribution in which the mean equals μ*_a_* and standard deviation equals *σ_a_*. The values of *a_j_*, GE, PS, μ*_a_*, *σ_a_*, and *σ_y_* were estimated as parameters. We set the uninformed priors of μ*_a_* to the uniform distribution [–∞, ∞], and those of *σ_a_* and *σ_y_* to the uniform distribution [0, ∞].

#### Model 3

We also conducted Model 3 using global region as a random effect affecting both the intercept and the slopes as follows:

(3) $$\begin{array}{lll} {\hat{y}}_{i} & = & a_{j}+{\text{GE}}_{j}x_{1,i}+{\text{PS}}_{j}x_{2,i},\\ a_{j} & ∼ & \text{Normal}(\mu_{a},\sigma_{a}),\\ {\text{GE}}_{j} & ∼ & \text{Normal}(\mu_{\text{GE}},\sigma_{\text{GE}}),\\ {\text{PS}}_{j} & ∼ & \text{Normal}(\mu_{\text{PS}},\sigma_{\text{PS}}),\\ y_{i} & ∼ & \text{Normal}({\hat{y}}_{i},\sigma_{y}). \end{array}$$

In this model, we assume that not only intercepts but also each effect of GE and PS differs according to the global region. GE*_j_* was also sampled from the normal distribution in which the mean equals μ_GE_ and standard deviation equals *σ*_GE_. PS*_j_* was also sampled in similar manner. μ_GE_ and μ_PS_ represent slopes globally affecting government effectiveness and pathogen stress, respectively. The values of *a_j_*, GE*_j_*, PS*_j_*, μ*_a_*, *σ_a_*, μ_GE_, *σ*_GE_, μ_PS_, *σ*_PS_, and *σ_y_* were estimated as parameters. We set the uninformed priors of μ*_a_*, μ_GE_, and μ_PS_ to uniform distribution [–∞, ∞], and those of *σ_a_*, *σ*_GE_, *σ*_PS_, and *σ_y_* to the uniform distribution [0, ∞].

In our reporting of results using GDP per capita as an independent variable instead of government effectiveness, we changed the names of parameters, representing the effects of institution (i.e., “GE”) for “GDP” in each model.

### Bayesian Estimation

We estimated the values of parameters in each model using Bayesian estimation. We conducted MCMC simulations with four independent chains in each model. A total of 5,000 iterations per chain were conducted, and first 1,000 were discarded as burn-in steps. We checked the convergence of the MCMC simulations using the Gelman–Rubin statistic (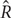 values) (Gelman and Rubin, 1992). 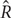 values were less than 1.10 for all parameters in each model, which means that the MCMC simulations converged. In addition, we also checked effective sample sizes (ESSs) of parameters, that is, the number of independent MCMC samples related to autocorrelation (the total number of MCMC samples was 16,000 samples). Greater ESSs are related to lower autocorrelation. In the **Supplementary Material**, we reported trace plots of MCMC simulations and the density plots of the posterior distributions of each parameter to understand visually whether or not the parameter values had converged. We estimated a 95% Bayesian credible interval for each parameter. If the interval did not contain zero, we interpreted the effect as significant. The MCMC simulations were conducted using the Stan and rstan package (Stan Development Team, 2017) with R v. 3.5.0 (R Core Team, 2018). Stan uses Hamiltonian Monte Carlo sampling for estimating parameters.

### Model Selection

We also evaluated the models by comparing the “Widely Applicable Information Criterion” (WAIC; Watanabe, 2010). WAIC values were calculated using the following definition, proposed in Gelman et al. (2013):

(4) $$\text{WAIC}=-2(\mathrm{lppd}-p_{\text{WAIC}}).$$

*lppd* means log pointwise posterior predictive density, and *p*_WAIC_ means effective number of parameters. The models with the smallest WAIC were selected as the best models. We calculated WAIC values using loo package with R.

## Results

### Zero-Order Correlations

We calculated the correlation coefficients (ρ) between dependent variables and independent variables (pathogen stress or government effectiveness) using Bayesian estimation (see the **Supplementary Method**). **Table 1** shows the zero-order correlation coefficients and their 95% Bayesian credible intervals. Like previous studies (Fincher et al., 2008; Murray and Schaller, 2010; Hruschka and Henrich, 2013), we also found that the regional historical pathogen stress was negatively and significantly correlated with the score for individualism. The regional scores for government effectiveness were positively and significantly correlated with it. The regional historical pathogen stress was significantly associated with all four indices of regional strength of conformity, and regional scores of government effectiveness were also significantly associated with indices of strengths of conformity. Note that *Conformity 4* was negatively correlated with pathogen stress and positively correlated with government effectiveness, since a higher score of *Conformity 4* indicates a willingness to be independent of others (“I seek to be myself rather than to follow others”). We also calculated the correlation coefficients between GDP per capita and conformity, and confirmed that it is strongly correlated with the individualism score and all four indices of conformity. We also confirmed that pathogen stress, government effectiveness, and GDP per capita were strongly correlated with each other. Pathogen stress was correlated strongly with government effectiveness (ρ : *Mean* = −0.68, *SD* = 0.04, *n* = 156, 95% CI [−0.76, −0.59], *ESS* = 10896) and GDP per capita (ρ : *Mean* = −0.60, *SD* = 0.05, *n* = 154, 95% CI [−0.70, −0.49], *ESS* = 12150). Government effectiveness was also strongly correlated with GDP per capita (ρ : *Mean* = 0.76, *SD* = 0.03, *n* = 197, 95% CI [0.70, 0.82], *ESS* = 9916). Hence, we decided not to enter all the three independent variables simultaneously to avoid the problem of multicollinearity. (We found that parameter estimation did not converge when the three independent variables were included in each multilevel model.)

**Table 1:** Posterior distribution of zero-order correlation coefficients between independent variables and dependent variables.

			Quantiles			Sample	
	*Mean*	*SD*	2.5%	50%	97.5%	Size	*ESS*
**Correlation coefficient of pathogen stress with**							
*Individualism*	−0.66	0.06	−0.76	−0.67	−0.54	100	11656
*Conformity 1*	0.71	0.06	0.59	0.71	0.81	83	10760
*Conformity 2*	0.54	0.08	0.37	0.55	0.68	81	13663
*Conformity 3*	0.49	0.08	0.33	0.50	0.63	94	14535
*Conformity 4*	–0.52	0.10	–0.70	–0.53	–0.30	51	12945
**Correlation coefficient of government effectiveness with**							
*Individualism*	0.68	0.05	0.57	0.69	0.78	103	10671
*Conformity 1*	–0.62	0.07	–0.74	–0.62	–0.47	85	12599
*Conformity 2*	–0.46	0.09	–0.62	–0.47	–0.28	83	16000
*Conformity 3*	–0.47	0.08	–0.62	–0.48	−0.30	95	16000
*Conformity 4*	0.43	0.11	0.19	0.44	0.64	51	14371
**Correlation coefficient of GDP per capita with**							
*Individualism*	0.63	0.06	0.50	0.63	0.74	102	12100
*Conformity 1*	−0.64	0.07	−0.75	−0.64	−0.50	84	11311
*Conformity 2*	−0.45	0.09	−0.61	−0.46	−0.27	82	14207
*Conformity 3*	−0.43	0.08	−0.58	−0.43	−0.25	94	14347
*Conformity 4*	0.46	0.11	0.22	0.47	0.66	50	14514

*ESS represents effective sample sizes of MCMC simulations.*

We also reported correlation coefficients between dependent variables in **Supplementary Table S2**. We confirmed that the individualism score was correlated negatively and significantly with the following three indices: *Conformity 1*, *Conformity 2*, and *Conformity 3*. *Conformity 4* was positively correlated with individualism, although the 95% Bayesian credible intervals included zero. The four indices of conformity were also correlated with each other, with the exception of the correlation between *Conformity 2* and *Conformity 3*, and that between *Conformity 3* and *Conformity 4.*

### Results of Bayesian Estimation

**FIGURE 1:**
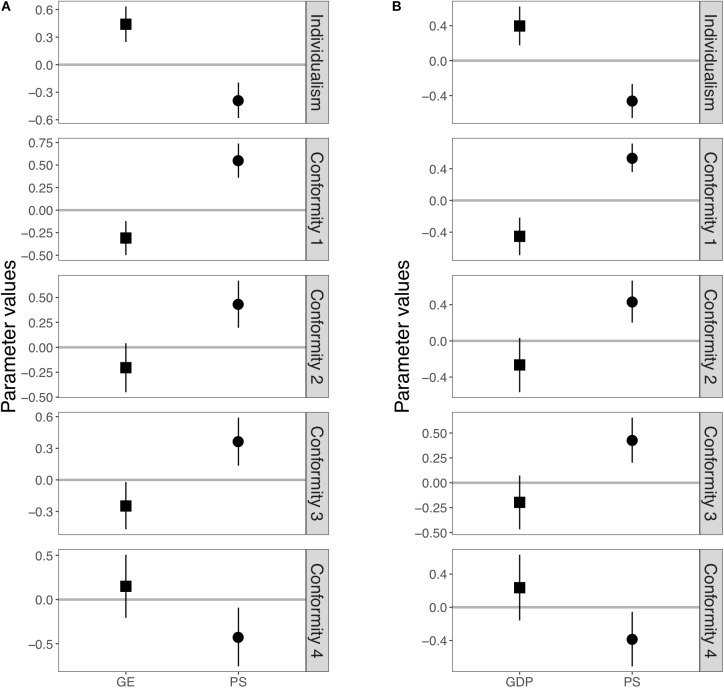
Posterior distributions of the estimated values of slopes in Model 1. **(A)** Results using government effectiveness as an independent variable. **(B)** Results using GDP per capita as an independent variable instead of government effectiveness. Squares and circles represent posterior mean of slope affecting government effectiveness (or GDP per capita) and pathogen stress, respectively. Each error bar represents a 95% Bayesian credible interval.

**Figure 1A** shows the inferred parameter values and the 95% Bayesian credible intervals in Model 1, which did not take the effect of shared cultural background into account (see **Supplementary Table S3** for numerical values). For the individualism score, it was confirmed that both pathogen stress (i.e., PS) and government effectiveness (i.e., GE) had a significant effect on it: that is, the 95% Bayesian credible intervals of both of two coefficients did not include zero. None of the indices of strength of conformity, the 95% Bayesian credible intervals of coefficients of pathogen stress included zero, which indicated that pathogen stress had significant positive effects on the regional strength of conformity. The coefficients of government effectiveness also had significant negative effects on *Conformity 1* and *Conformity 3*, but not on *Conformity 2* or *Conformity 4*.

**FIGURE 2:**
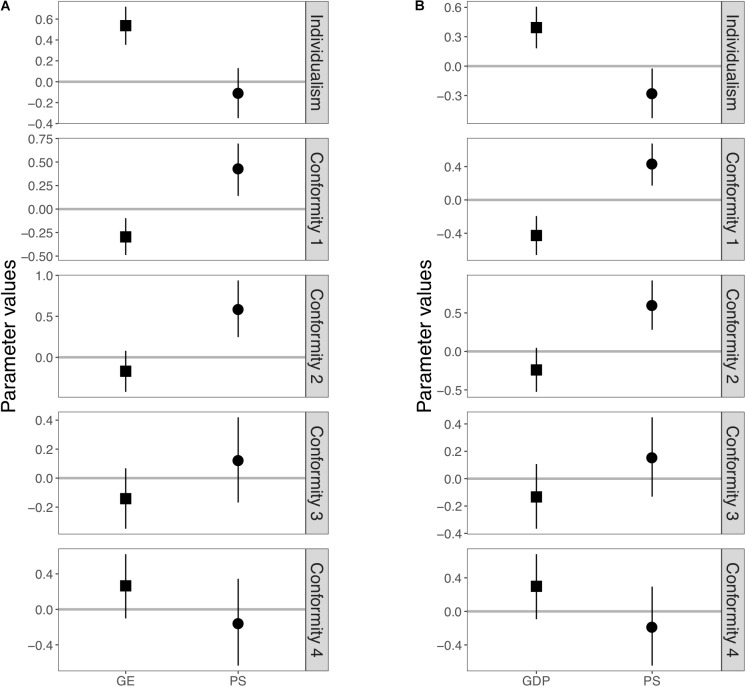
Posterior distributions of the estimated values of slopes in Model 2. **(A)** Results using government effectiveness as an independent variable. **(B)** Results using GDP per capita as an independent variable instead of government effectiveness. Squares and circles represent posterior mean of slope affecting government effectiveness (or GDP per capita) and pathogen stress, respectively. Each error bar represents a 95% Bayesian credible interval.

**Figure 2A** illustrated the results of Model 2, a random intercept model in which global regions were used as random intercepts. This model assumes that the effects of pathogen stress and government effectiveness are common to all global regions (see **Supplementary Table S4** for numerical values). Pathogen stress was not significantly related to the individualism score, whereas government effectiveness had still a significant effect on it. As noted in the introduction, Hruschka and Henrich (2013) found that government effectiveness could explain regional variations in individualism when they controlled for both pathogen stress and global regions using a standard regression model. However, for indices of conformity, pathogen stress still had significant positive effects on two indices. *Conformity 1* and *Conformity 2*, after both government effectiveness and global regions were controlled for, although the effects on the indices, *Conformity 3* and *Conformity 4*, disappeared. Also, government effectiveness still had a significant negative effect on *Conformity 1*. However, its effect on *Conformity 3*, which was significant in Model 1, disappeared in Model 2.

**FIGURE 3:**
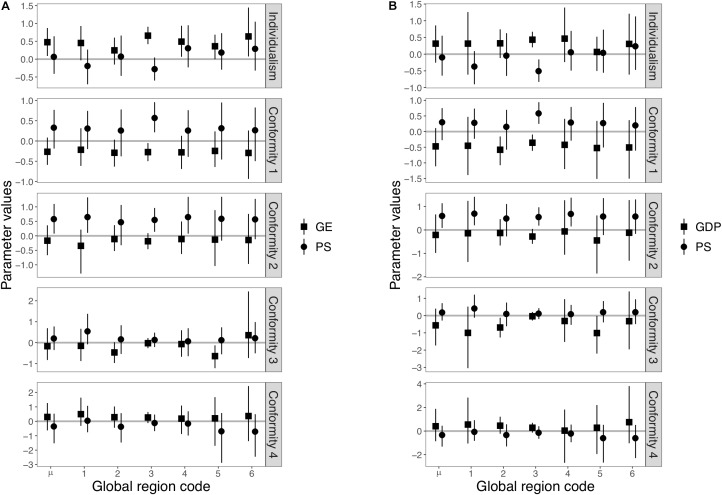
Posterior distributions of the estimated values of slopes in Model 3. **(A)** Results using government effectiveness as an independent variable. **(B)** Results using GDP per capita as an independent variable instead of government effectiveness. Squares and circles represent posterior means of slopes affecting government effectiveness (or GDP per capita) and pathogen stress, respectively. Each number on the horizontal axis represents a global region (1 = Sub-Saharan Africa, 2 = East Asia and Pacific, 3 = Europe and Central Asia, 4 = Latin America and Caribbean, 5 = Middle East and North Africa, and 6 = South Asia). μ in horizontal axis represents global slopes across global regions (i.e., μ_GE_, μ_GDP_, or μ_PS_). Each error bar represents a 95% Bayesian credible interval.

**Figure 3A** shows the results of Model 3, in which global regions were treated as both random slopes and intercepts. **Figure 3** shows means and 95% Bayesian credible intervals of parameters estimated for each global region (see **Supplementary Table S5** for numerical values). For the individualism score, the global effect of government effectiveness (i.e., μ_GE_) remained significant. Significant region-specific effects of government effectiveness on slopes were also found in three global regions (Europe and Central Asia, Latin America and Caribbean, and South Asia). Both global and region-specific effects of pathogen stress were insignificant. For the index of *Conformity 1*, we found that the global effects of both pathogen stress and government effectiveness across global regions were no longer significant (i.e., the 95% Bayesian credible intervals of μ_PS_ and μ_GE_ included zero). However, a significant region-specific effect of pathogen stress on slope was observed in Europe and Central Asia. A significant region-specific effect of government effectiveness was also observed in this region. For the index of *Conformity 2*, the global effect of pathogen stress (i.e., μ_PS_) remained significant. As with Model 2, the global effect of government effectiveness (i.e., μ_GE_) was not significant. Significant region-specific effects of pathogen stress on slopes were also found in three global regions (Sub-Saharan Africa, Europe and Central Asia, and Latin America and Caribbean). On the other hand, no significant region-specific slope effects of government effectiveness were found in any of the global regions. For the index of *Conformity 3*, we found that neither the global slope effects of pathogen stress nor government effectiveness were significant. None of the region-specific effects of pathogen stress on slopes were significant in any global region, while the negative region-specific effects of government effectiveness were significant in Middle East and North Africa. For the index of *Conformity 4*, neither the global and region-specific effects of pathogen stress nor government effectiveness were significant. **Supplementary Figures S1**–**S10** illustrates the relationship between government effectiveness or pathogen stress and each index by global region.

We summarize the results of above analysis here (see also **Supplementary Table S6** for additional details of the following summary). For the individualism score, the global effects of pathogen stress, which was significant in Model 1, disappeared in both Model 2 and Model 3, whereas the significant global effects of government effectiveness remained in all models. For the indices of conformity, the global effects of pathogen stress, which were significant for all four conformity indices in Model 1, remained significant for two indices in Model 2, and for only one index in Model 3. The global effects of government effectiveness that were significant for two conformity indices in Model 1 remained significant for only one index in Model 2, and for none in Model 3. These results suggest that the previous studies may have suffered from Galton’s problem, and the relationship between the collectivism, conformity, pathogen prevalence, and government effectiveness may not be universal phenomena. On the other hand, in Model 3, several region-specific effects were found to be significant. The region-specific effects of pathogen stress were significant in Europe and Central Asia for *Conformity 1* and *2*, and in Sub-Saharan Africa, Latin America, and Caribbean, for *Conformity 2*. For the Individualism, the region-specific effect of government effectiveness was significant in Europe and Central Asia, Latin America and Caribbean, and South Asia. The same region-specific effects were also significant in the Europe and Central Asia for the *Conformity 1*, and in the Middle East and North Africa for the *Conformity 3*.

Here, we reported additional tests using GDP per capita as the independent variable instead of government effectiveness (see **Figures 1B**, **2B**, **3B**, **Supplementary Figures S11**–**S15**, and **Supplementary Tables S7**–**S9**). See also **Supplementary Table S10** for further details on the following summary. We confirmed that the overall results using GDP as the independent variable were similar to the previous results. First, the global effects of pathogen stress, which were significant for individualism and all four conformity indices in Model 1, remained significant for individualism and two indices (*Conformity 1* and *2*) in Model 2. The global effects of GDP, which were significant for individualism and *Conformity 1* in Model 1, remained significant in Model 2. In Model 3, the global effects of pathogen stress remained significant for only *Conformity 2*, whereas that of GDP was not significant for any indices. These results are consistent with previous analyses and imply that the effects of pathogen stress and government effectiveness are not universal.

Most region-specific effects of pathogen stress were also replicated. As with the previous analyses, the region-specific effect of pathogen stress was again found significant in Europe and Central Asia for *Conformity 1* and *2*, and in Sub-Saharan Africa, Latin America and Caribbean, for *Conformity 2*. In addition, we found region-specific effects of pathogen stress in Europe and Central Asia for individualism, whereas this was not found in previous analyses. Patterns of region-specific effects of GDP differed from those for government effectiveness. Region-specific effects of GDP were found significant in Europe and Central Asia for Individualism and *Conformity 1*. We were unable to find significant region-specific effects for GDP in Latin America and Caribbean or South Asia for individualism, whereas effects were found for government effectiveness. Moreover, region-specific effects of GDP, which were not significant in previous analyses, were also found to be significant in East Asia and Pacific for *Conformity 1* and *Conformity 3*.

### Model Selection

**Table 2** shows the WAIC values for each model. For all combinations of dependent and independent variables, the WAIC for hierarchical models (i.e., Model 2 or Model 3) was consistently lower than a non-hierarchical model (i.e., Model 1). For *Conformity 4*, even though the WAIC value for Model 3 was higher than Model 1, the WAIC value for Model 2 was the lowest of all three models. These results indicate that hierarchical models considering region-specific effects are better and more appropriate than those of a standard non-hierarchical linear model, as was employed in the previous studies. The results of comparisons between Model 2 and Model 3 are mixed. Regardless of the type of independent variables, Model 3 fit better for the individualism score and *Conformity 3*; otherwise, Model 2 was better than Model 3. The results of the best fit models suggest that, when government effectiveness was controlled for, the global effects of pathogen stress (i.e., PS) remained significant only for two out of four indices of conformity (*Conformity 1* and *Conformity 2*). Similar results were obtained when GDP per capita was controlled; the global effect of pathogen prevalence remained significant for the same two conformity indices (*Conformity 1* and *Conformity 2*).

**Table 2:** WAIC values of each model (IND: *Individualism*, C1: *Conformity 1*, C2: *Conformity 2*, C3, *Conformity 3*, C4: *Conformity 4*).

Results in which government effectiveness was used as an independent variable					

	*IND*	*C1*	*C2*	*C3*	*C4*
Model 1	212.53	174.28	203.56	238.08	133.62
Model 2	189.86	170.35	197.42	213.25	133.41
Model 3	181.86	172.88	200.15	211.90	137.57

**Results in which GDP per capita was used as an independent variable**					

	***IND***	***C1***	***C2***	***C3***	***C4***

Model 1	218.11	168.63	201.78	237.46	131.04
Model 2	208.89	164.37	194.30	212.01	130.45
Model 3	202.40	167.66	197.23	210.05	133.99

## Discussion

Previous studies have consistently suggested that the historical prevalence of pathogens played an important role in explaining the cultural differences in group-oriented psychology. However, the correlation between pathogen stress and human collectivistic psychology found in previous studies may suffer from the problem of statistical non-independence, called Galton’s problem. We checked the robustness of the effect of pathogen stress on collectivism and conformity using a hierarchical linear model with MCMC simulations. First, we successfully reproduced the significant effects of regional pathogen stress on collectivism and all the four indicators of conformity without adjusting the effect of global regions. When adjusting the global regions to the random intercept model, significant global effects of pathogen stress remained on only two of the four indicators of conformity. Moreover, in the models including both random slopes and intercepts, the global effects of pathogen stress remained significant for only one indicator. Significant effects of pathogen stress were limited only in some global regions. However, these effects did not seem to be robust. For instance, the most robust local effect was found in Europe and Central Asia, but it was limited to only two out of four indicators of conformity. Model selection revealed that hierarchical models that took into account region-specific effects (Models 2 or 3) improved predictive accuracy more than a non-hierarchical model (Model 1). These results suggested the existence of spatial autocorrelations between countries that share a global region, and such correlations must be statistically controlled. For some indices of conformity, Model 2 fit to the data better than Model 3 did. For *Conformity 4*, predictive accuracy of Model 3 was worst in three models. This indicated that random slopes may be redundant parameters for the prediction of the variances of the regional level of conformity. However, even when only the best models are considered, we can conclude that either global or region-specific effects of pathogen stress are limited to only two indices of conformity.

Group-level analyses, which are often conducted in cross-cultural research, inevitably suffer from Galton’s problem (Mace and Pagel, 1994; Nettle, 2009) as neighboring groups or countries occasionally share a common descent and cannot be assumed to be statistically independent units. Two other approaches that do not use Bayesian multilevel modeling may be effective for handling this problem. One is the use of standard regression models using the effect of global regions as intercepts, an approach employed by Hruschka and Henrich (2013). The other is a standard regression model with interaction terms between the global region and independent variables. However, the Bayesian multilevel model approach has several advantages over these approaches. The Bayesian multilevel analysis with MCMC simulation can simultaneously estimate the region-specific effect (as the random effects of both slopes and intercepts) and the global effects. This is achieved by assuming that each effect of global region (e.g., PS*_j_* in our Model 3) is produced by a common variable (e.g., μ_PS_ in our Model 3). Under this assumption, we can avoid the problem of overfitting (i.e., the results of analysis fit to existing data well, but could not be generalized to unseen new data) even when the number of samples is small. Furthermore, the results of Bayesian multilevel models are in general intuitively interpretable.

Our analyses can be improved in the future. In our approach, selection of the global regions plays an important role and should influence model results. We followed Hruschka and Henrich (2013) in categorizing the countries into six global regions. However, not all the countries belonging to a global region necessarily share identical historical or cultural roots. The best method for adjusting the effects of shared ancestry is a phylogenetic approach (Mace and Pagel, 1994; Mathew and Perreault, 2015). Since it is usually difficult to reconstruct cultural phylogenetic trees of modern countries, the multilevel model is an effective candidate for partly overcoming Galton’s problem (Nettle, 2009). Further analyses, using different categorizations of global regions with variables other than conformity and collectivism that were found to be correlated with the prevalence of pathogens, are required to rigorously test the robustness of the effects of pathogen stress on the collectivistic psychological mechanisms.

Recent studies have raised doubts about the effect of pathogen stress on the development of collectivistic psychological mechanisms. As noted in the introduction, Hruschka and Henrich (2013) indicated that modern institutions rather than pathogen stress impact collectivism and in-group favoritism. In addition, Talhelm et al. (2014) indicated that forms of subsistence (e.g., rice-growing) rather than pathogen stress affected collectivistic styles of thinking. In contrast to these studies, recent studies suggested that regional levels of pathogen stress might be related to adherence to social norms, but not to negativity toward outgroups (Cashdan and Steele, 2013; Tybur et al., 2016). In the current study, we found that pathogen stress, if it exists, is related to only some group-oriented psychological mechanisms, such as concerns about evaluation by community members. Because the concept of collectivism includes many different group-oriented psychological mechanisms, scholars must divide it into its core aspects and test the effect of environmental severity on each of these.

A problem regarding the restricted samples from WEIRD (Western, Educated, Industrial, Rich, and Democratic) societies has been recently noted in cross-cultural studies (Henrich et al., 2010). Our analysis seems to illuminate the related possibility that pathogen stress has a significant effect on collectivism or conformity only in restricted global regions, such as European and Asian societies. However, the dataset still did not seem to be sufficient for making bold conclusions about region-specific effects. In general, Bayesian credible intervals (i.e., variance of posterior distributions) become larger as a sample size gets smaller. Significant region-specific effects of Europe and Central Asia might have been statistical artifacts stemming from a larger sample size in this region (for instance, relative to a few countries in the South Asia region). If mean values of significant region-specific effects are close to those of non-significant effects while standard deviations of the significant region-specific effects are much smaller, differences in sample size might cause such artifacts. Close inspection of means of the posterior distributions (**Figure 3** and **Supplementary Tables S5**, **S9**) suggests that this problem might have arisen on *Conformity 2* in Model 3 but not in the other cases. Although one advantage of using Bayesian estimation with MCMC simulation is that it can estimate parameter values even with small samples, Bayesian credible intervals need to be carefully interpreted. Additional data need to be included in future analyses to identify the robustness of region-specific effects of pathogen prevalence on collectivistic psychological mechanisms.

## Author Contributions

YH and MT contributed equally to the research design, data analysis, and writing of the manuscript.

## Conflict of Interest Statement

The authors declare that the research was conducted in the absence of any commercial or financial relationships that could be construed as a potential conflict of interest.

## Footnotes

1 https://www.hofstede-insights.com
