# Supplementary material for: Cultural Differences in Strength of Conformity Explained Through Pathogen Stress: A Statistical Test Using Hierarchical Bayesian Estimation
Source: Front Psychol. 2018 Oct 11;9:1921. doi: 10.3389/fpsyg.2018.01921 (PMC6193438; doi:10.3389/fpsyg.2018.01921)
Supplement: Supplementary file 1 [file Data_Sheet_1.PDF]

Supplementary material for "Cultural differences in strength of conformity explained through pathogen stress: A statistical test using hierarchical Bayesian estimation"

Yutaka Horita & Masanori Takezawa

Supplementary Figures

Supplementary Figure S1–S15: Scatter plots displaying the correlations between regional level of government effectiveness (pathogen stress or GDP per capita) and each index of regional strength of conformity by each global region.

Supplementary Tables

Supplementary Table S1: The number of countries or regions in each global region used for analysis.

Supplementary Table S2: Posterior distribution of zero-order correlation coefficients between dependent variables.

Supplementary Table S3: Numerical values of estimated parameters in Model 1 using both pathogen stress and government effectiveness as independent variables.

Supplementary Table S4: Numerical values of estimated parameters in Model 2 using both pathogen stress and government effectiveness as independent variables.

Supplementary Table S5: Numerical values of estimated parameters in Model 3 using both pathogen stress and government effectiveness as independent variables.

Supplementary Table S6: Summary of analysis results using both pathogen stress and government effectiveness as independent variables.

Supplementary Table S7: Numerical values of estimated parameters in Model 1 using both pathogen stress and GDP per capita as independent variables.

Supplementary Table S8: Numerical values of estimated parameters in Model 2 using both pathogen stress and GDP per capita as independent variables.

Supplementary Table S9: Numerical values of estimated parameters in Model 3 using both pathogen stress and GDP per capita as independent variables.

Supplementary Tables S10: Summary of analysis results using both pathogen stress and GDP per capita as independent variables.

Supplementary Method

Bayesian estimation of zero-order correlation coefficients

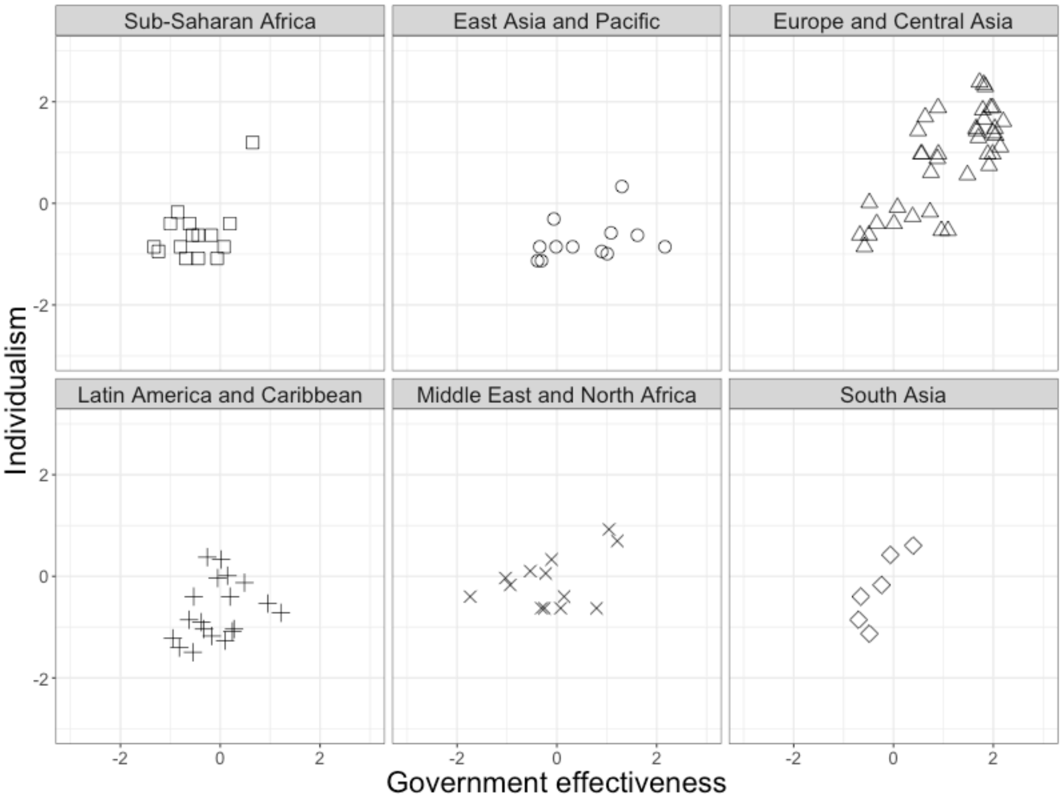

Supplementary Figure S1: Scatter plots displaying the correlation between regional level of government effectiveness and the indexes of *Individualism* by global region. Each point represents a country or region.

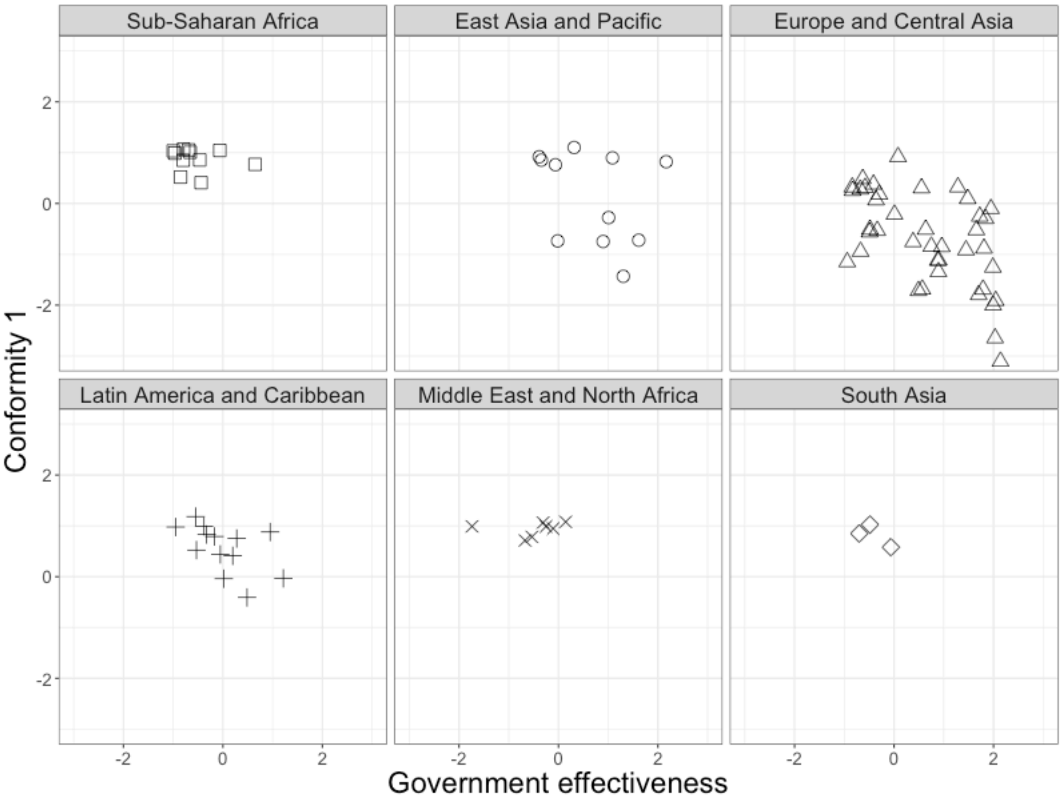

Supplementary Figure S2: Scatter plots displaying the correlation between regional level of government effectiveness and the indexes of *Conformity 1* by global region. Each point represents a country or region.

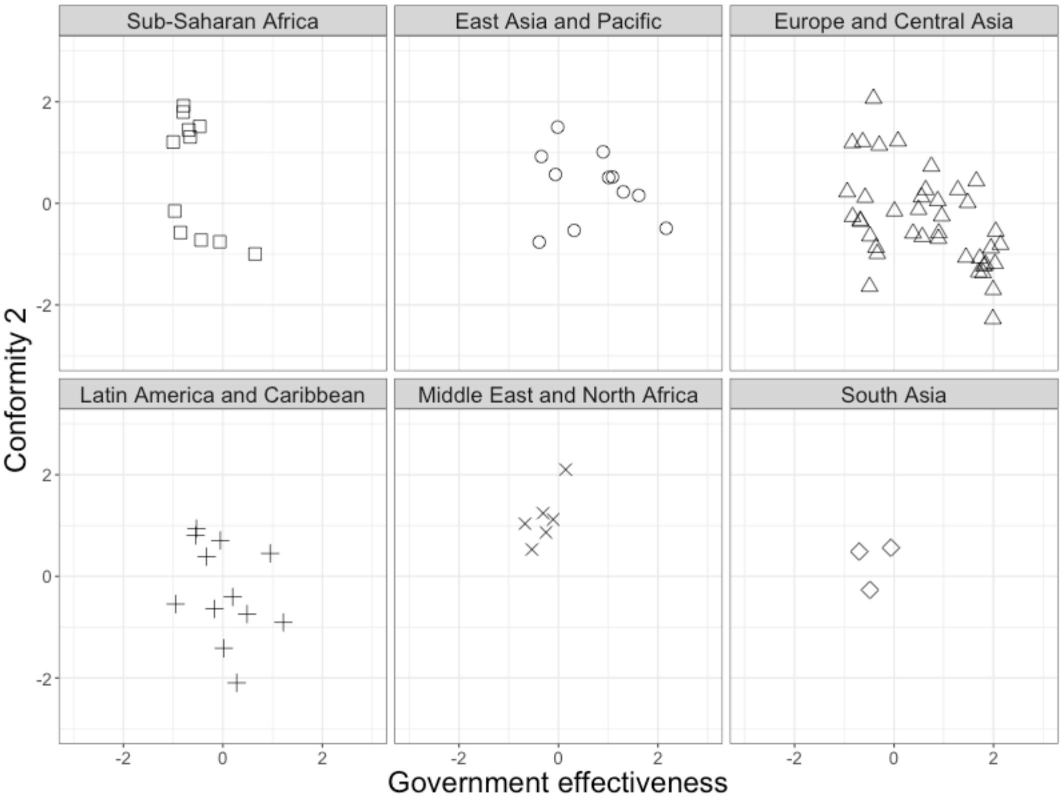

Supplementary Figure S3: Scatter plots displaying the correlation between regional level of government effectiveness and the indexes of *Conformity 2* by global region. Each point represents a country or region.

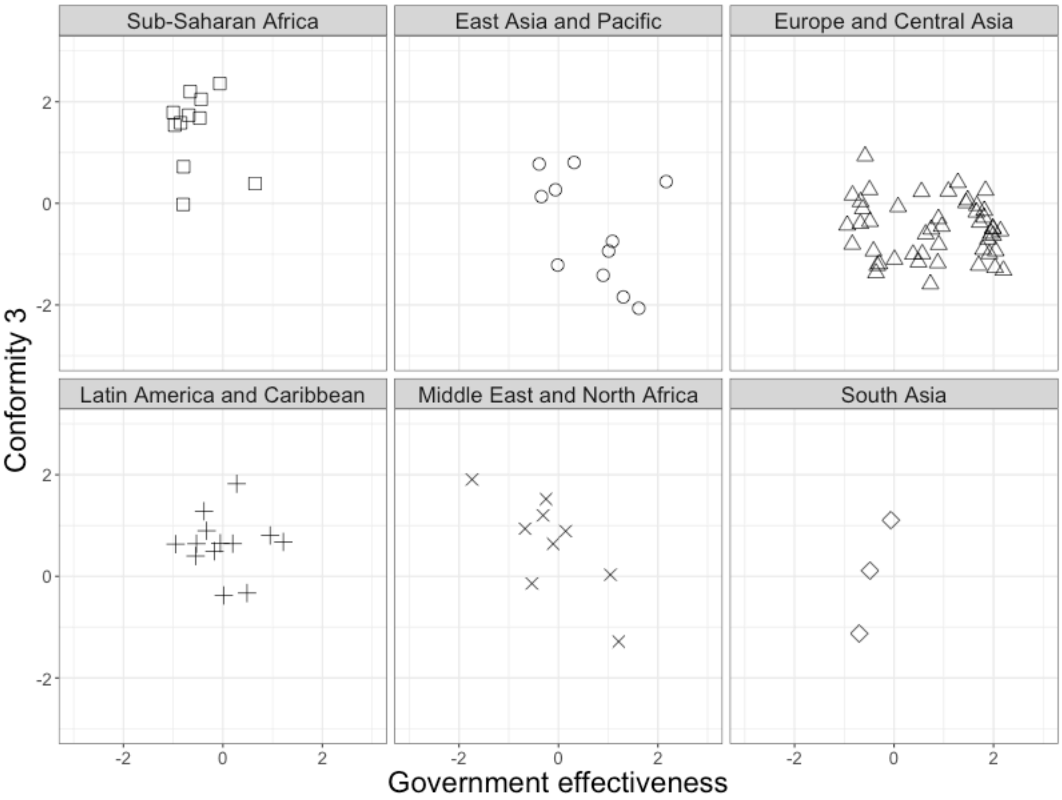

Supplementary Figure S4: Scatter plots displaying the correlation between regional level of government effectiveness and the indexes of *Conformity 3* by global region. Each point represents a country or region.

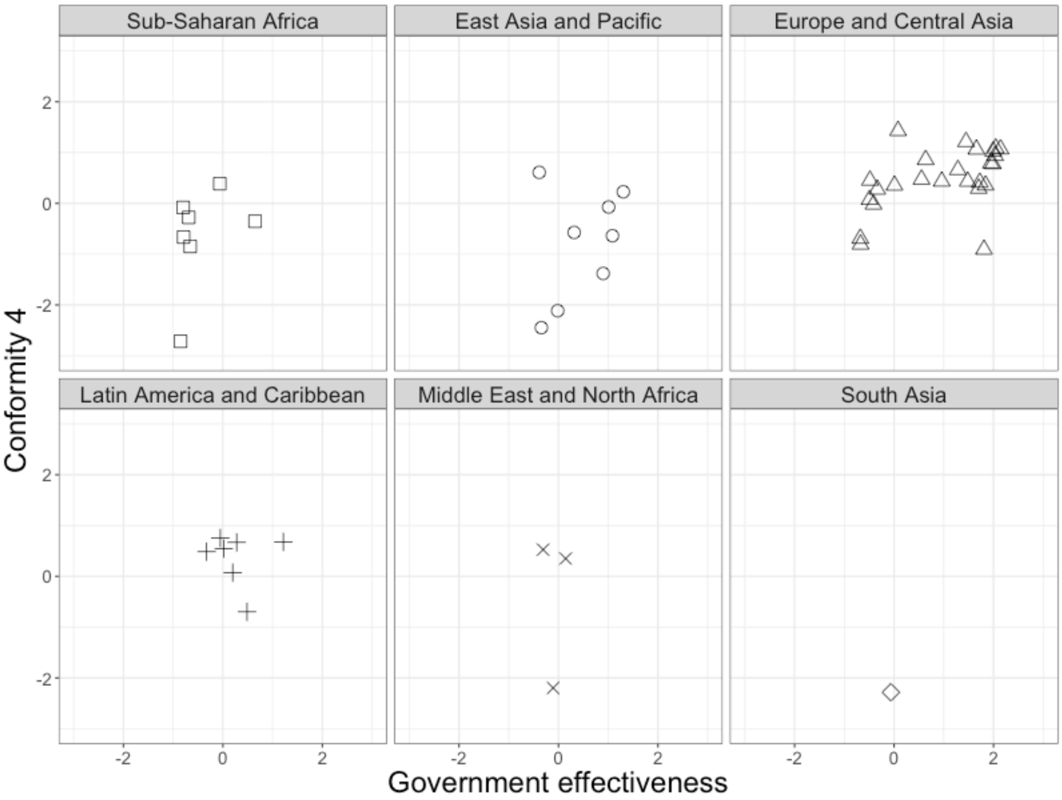

Supplementary Figure S5: Scatter plots displaying the correlation between regional level of government effectiveness and the indexes of *Conformity 4* by global region. Each point represents a country or region.

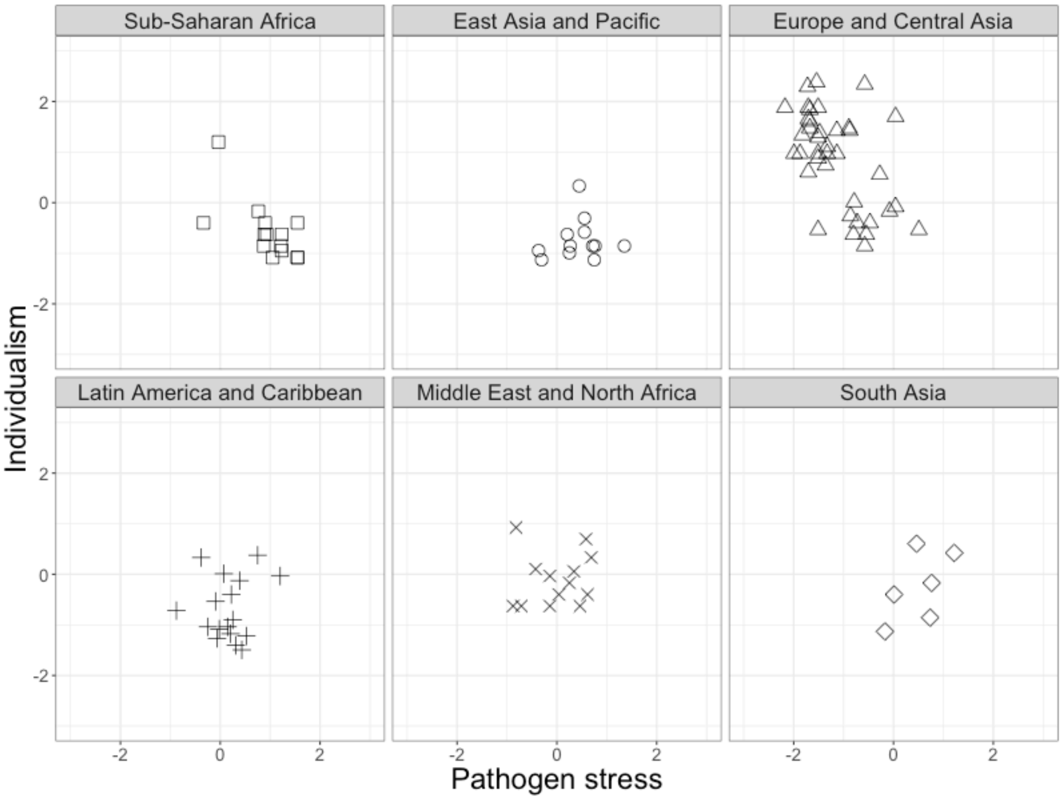

Supplementary Figure S6: Scatter plots displaying the correlation between regional level of pathogen stress and the index of *Individualism* by each global region. Each point represents a country or region.

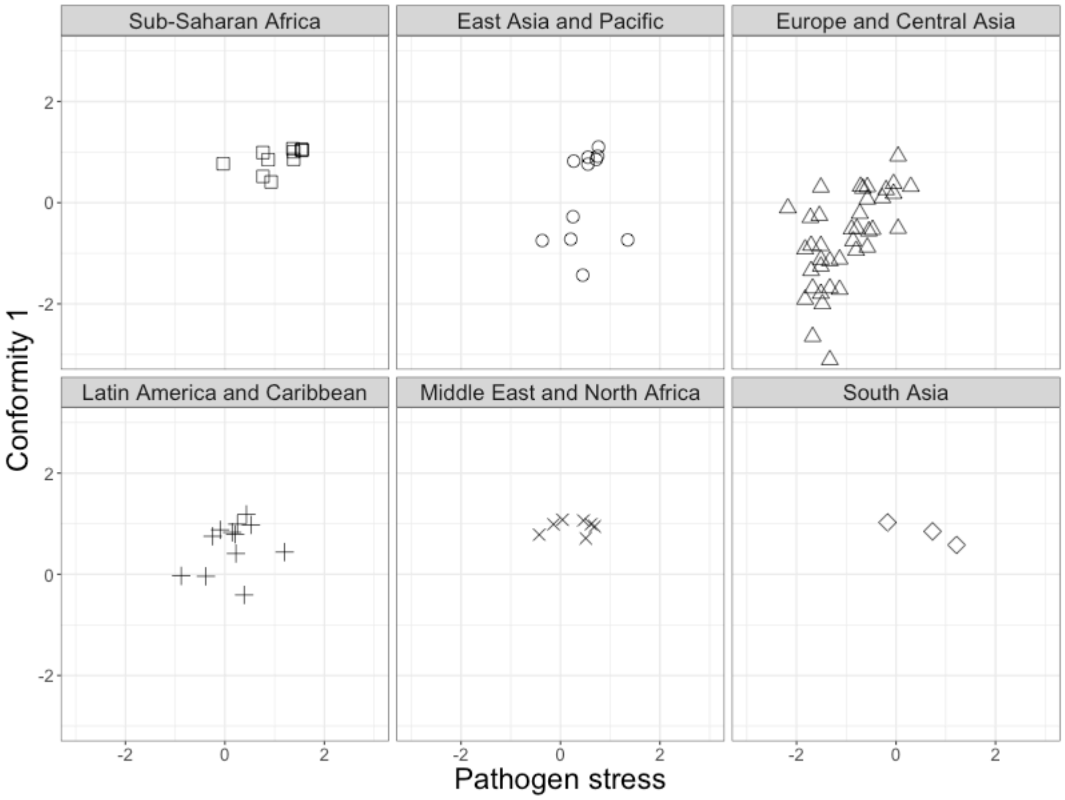

Supplementary Figure S7: Scatter plots displaying the correlation between regional level of pathogen stress and the index of *Conformity 1* by each global region. Each point represents a country or region.

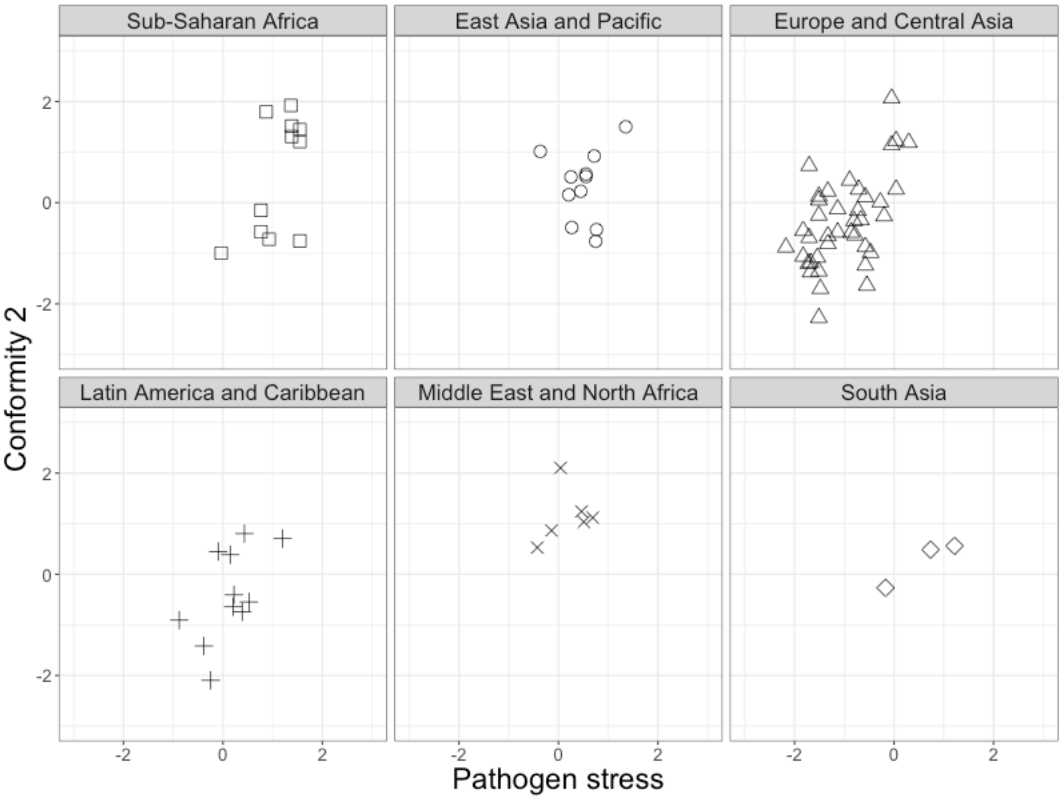

Supplementary Figure S8: Scatter plots displaying the correlation between regional level of pathogen stress and the index of *Conformity 2* by global region. Each point represents a country or region.

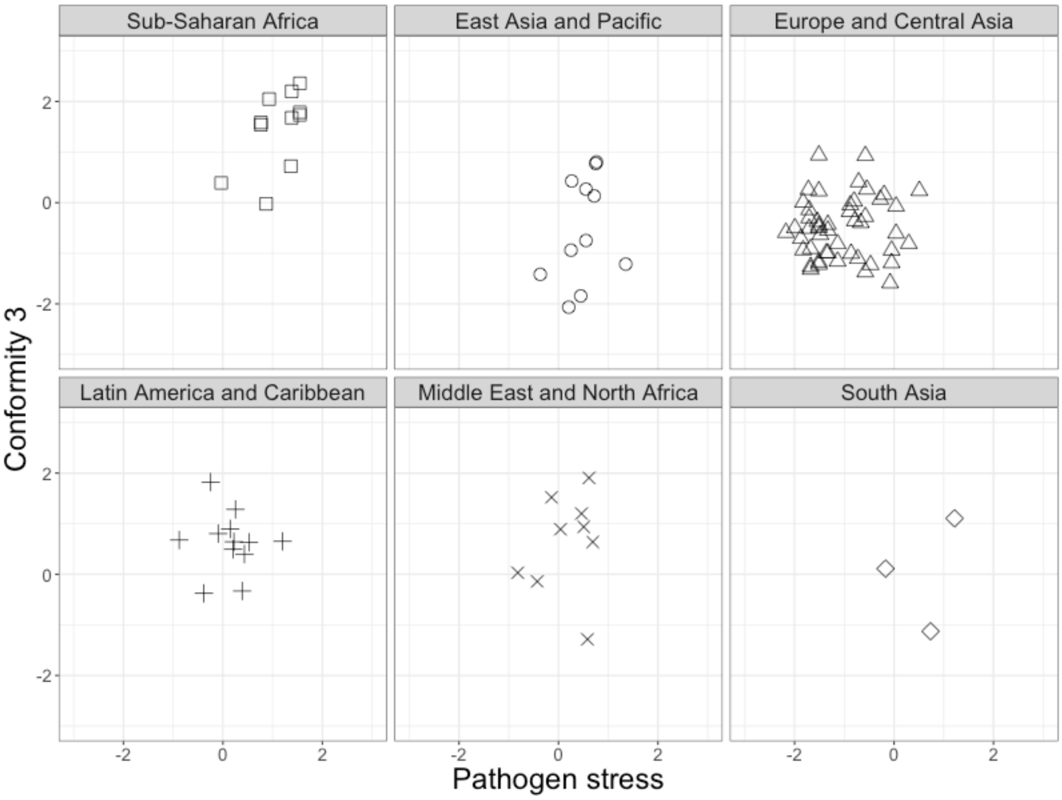

Supplementary Figure S9: Scatter plots displaying the correlation between regional level of pathogen stress and the index of *Conformity 3* by global region. Each point represents a country or region.

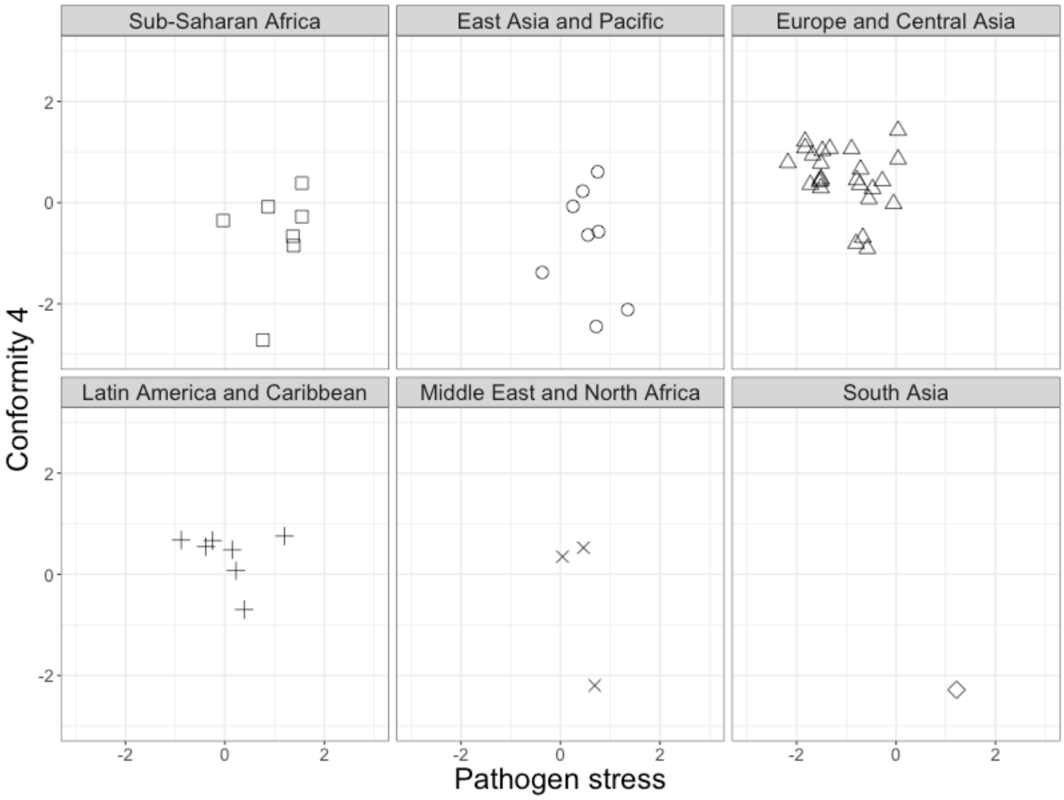

Supplementary Figure S10: Scatter plots displaying a correlation between regional level of pathogen stress and the index of *Conformity 4* by global region. Each point represents a country or region.

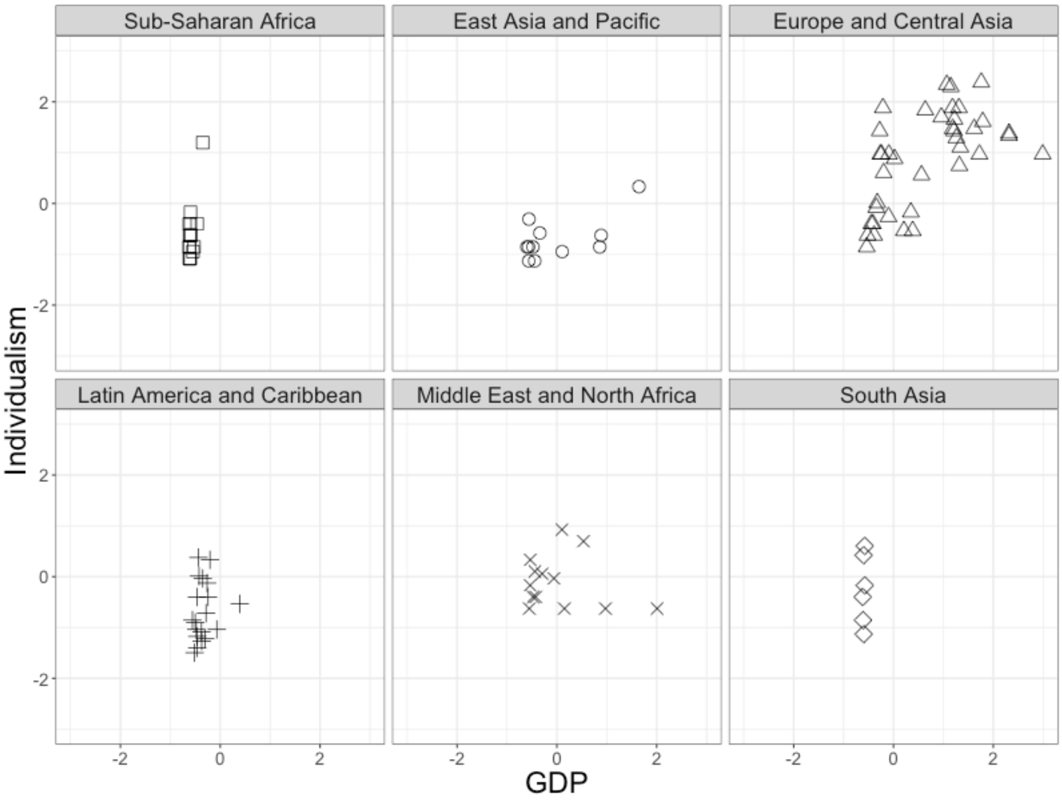

Supplementary Figure S11: Scatter plots displaying the correlation between regional level of GDP per capita and the index of *Individualism* by global region. Each point represents a country or region.

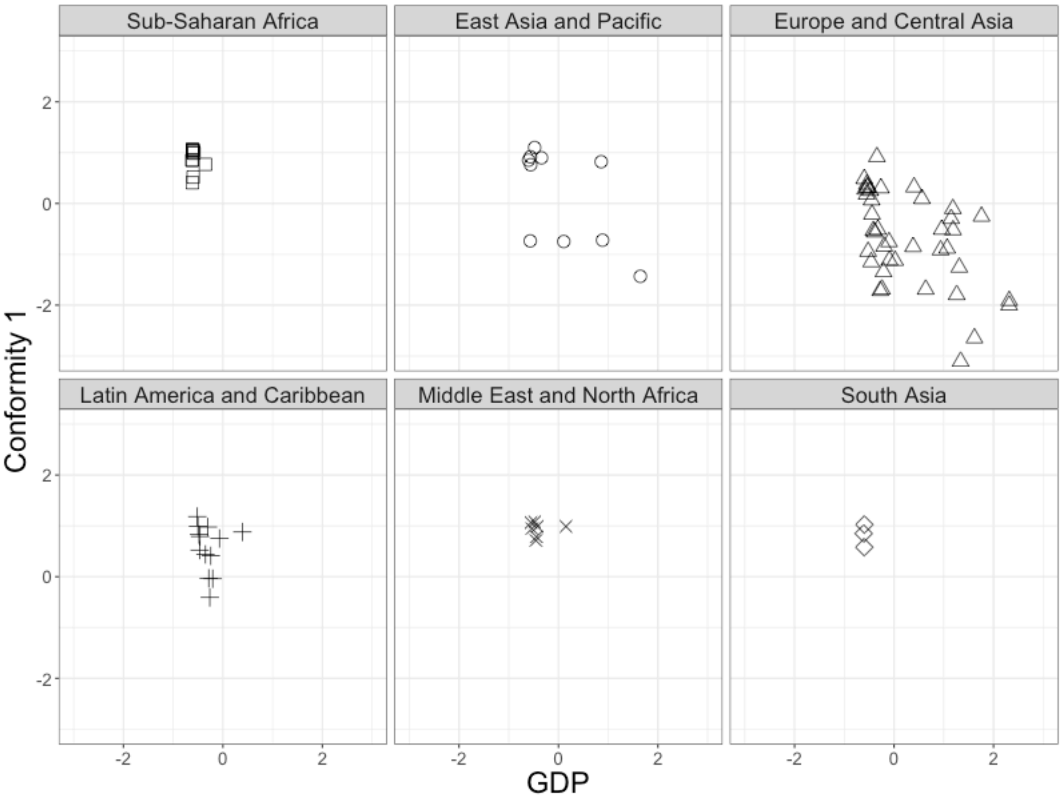

Supplementary Figure S12: Scatter plots displaying the correlation between regional level of GDP per capita and the index of *Conformity 1* by global region. Each point represents a country or region.

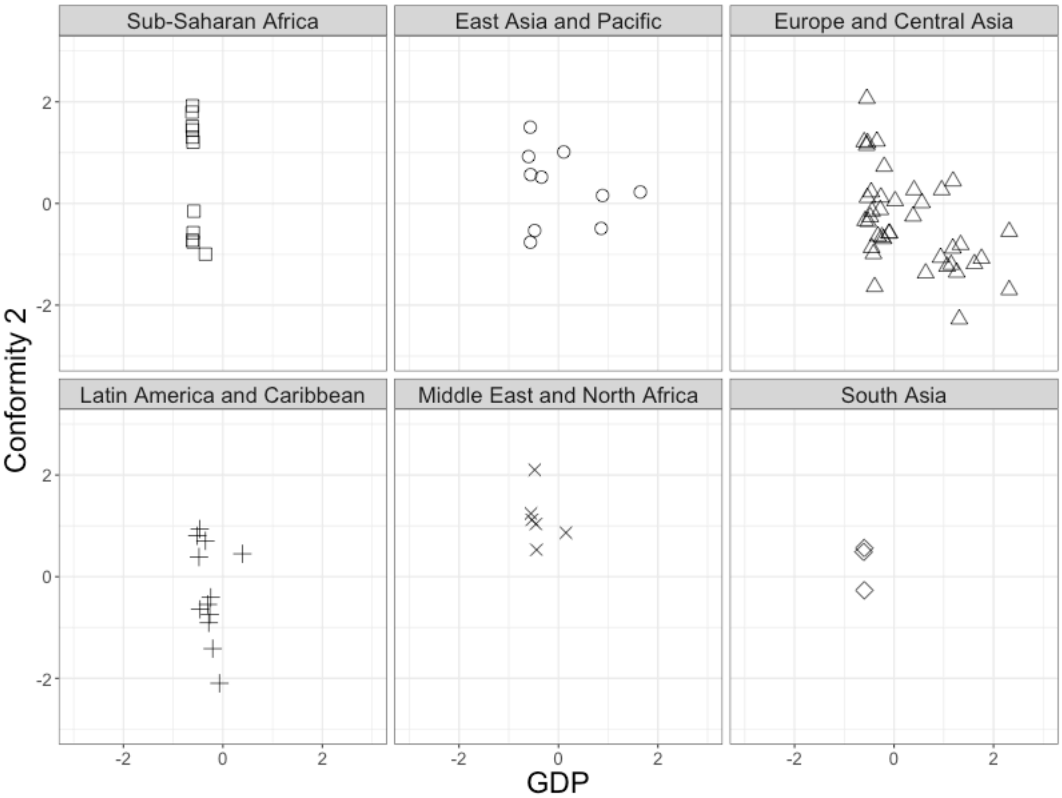

Supplementary Figure S13: Scatter plots displaying the correlation between regional level of GDP per capita and the index of *Conformity 2* by global region. Each point represents a country or region.

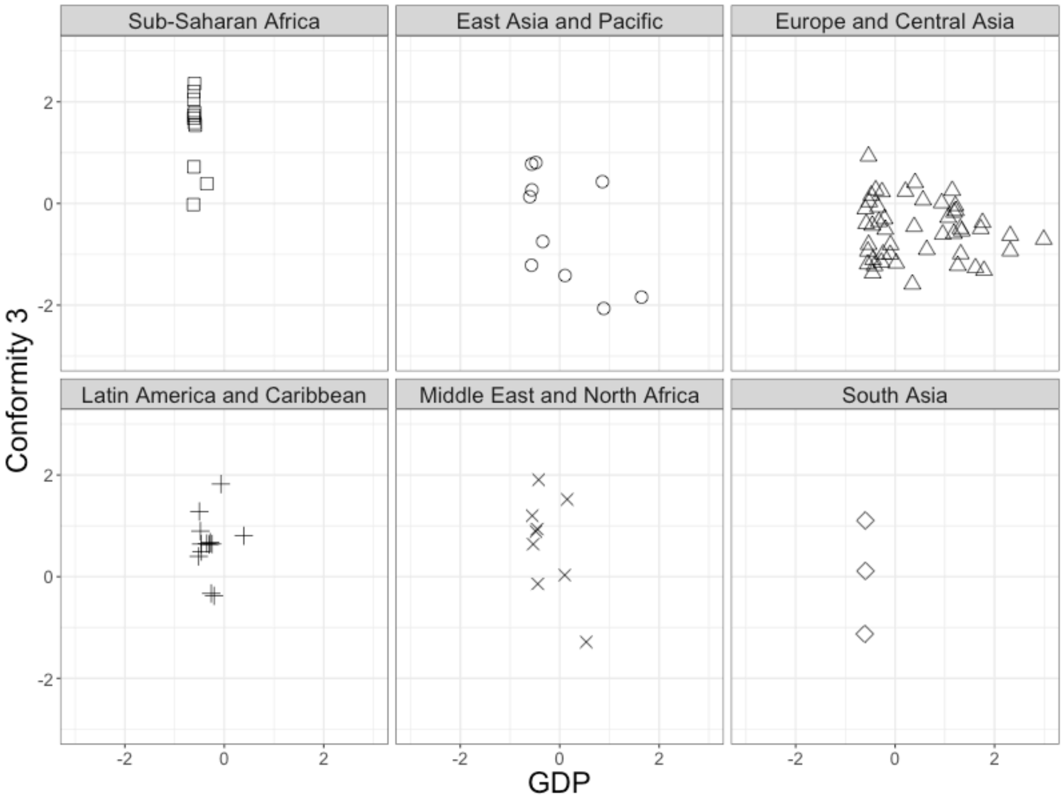

Supplementary Figure S14: Scatter plots displaying the correlation between regional level of GDP per capita and the index of *Conformity 3* by global region. Each point represents a country or region.

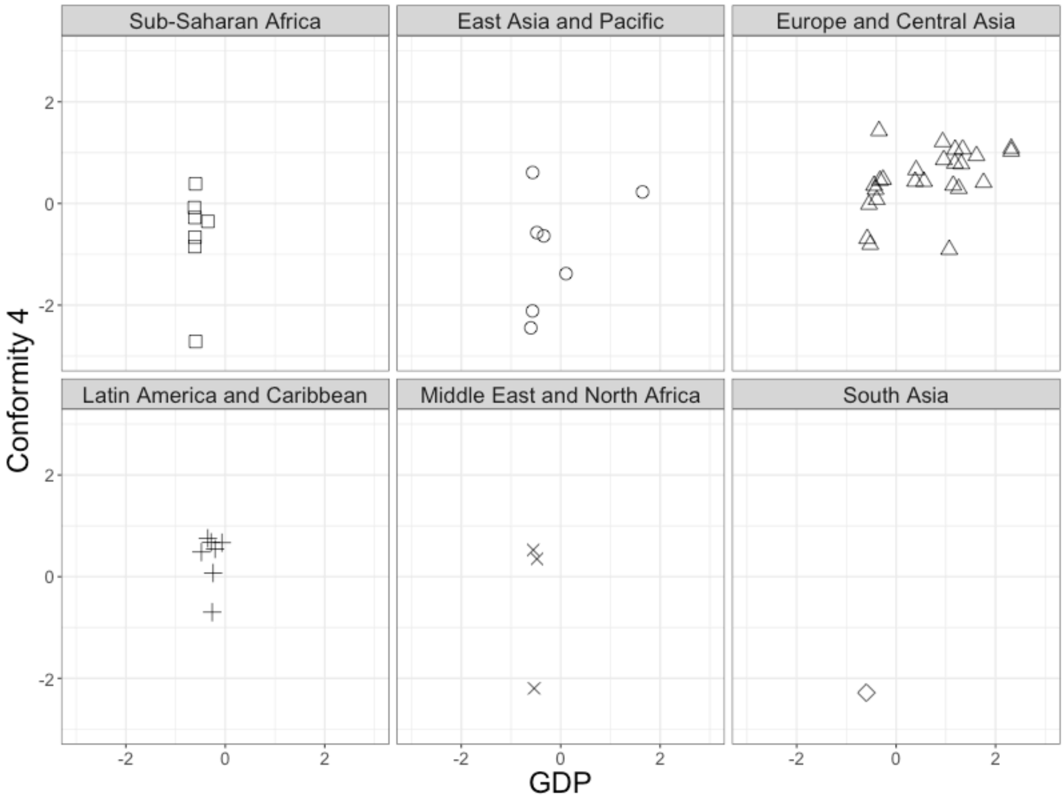

Supplementary Figure S15: Scatter plots displaying the correlation between regional level of GDP per capita and the index of *Conformity 4* by global region. Each point represents a country or region.

Supplementary Table S1: The number of countries or regions in each global region used for analysis (GE: government effectiveness, PS: pathogen stress, IND: *Individualism*, C1: *Conformity 1*, C2: *Conformity 2*, C3, *Conformity 3*, C4: *Conformity 4*).

	Dependent variables				
	IND	C1	C2	C3	C4
GE and PS were used as independent variables					
Sub-Saharan Africa	14	11	11	11	7
East Asia and Pacific	12	11	11	11	8
Europe and Central Asia	38	39	39	47	25
Latin America and Caribbean	17	12	11	12	7
Middle East and North Africa	13	7	6	9	3
South Asia	6	3	3	3	1
Total	100	83	81	93	51
GDP per capita and PS were used for independent variables					
Sub-Saharan Africa	14	11	11	11	7
East Asia and Pacific	11	10	10	10	7
Europe and Central Asia	38	39	39	47	25
Latin America and Caribbean	17	12	11	12	7
Middle East and North Africa	13	7	6	9	3
South Asia	6	3	3	3	1
Total	99	82	80	92	50

Supplementary Table S2: Posterior distribution of zero-order correlation coefficients between dependent variables. *ESS* represents effective sample sizes of MCMC simulations.

			Quantiles			Sample	
	Mean	*SD*	2.5%	50%	97.5%	size	*ESS*
Individualism and Conformity 1	–0.62	0.08	–0.75	–0.62	–0.45	70	12142
Individualism and Conformity 2	–0.40	0.10	–0.59	–0.40	–0.18	68	14609
Individualism and Conformity 3	–0.40	0.09	–0.58	–0.41	–0.21	80	14885
Individualism and Conformity 4	0.27	0.14	–0.03	0.27	0.52	45	16000
Conformity 1 and Conformity 2	0.47	0.09	0.29	0.47	0.63	83	16000
Conformity 1 and Conformity 3	0.66	0.06	0.53	0.66	0.77	85	10564
Conformity 1 and Conformity 4	–0.30	0.13	–0.53	–0.31	–0.04	51	16000
Conformity 2 and Conformity 3	0.15	0.11	–0.06	0.15	0.36	83	16000
Conformity 2 and Conformity 4	–0.32	0.13	–0.55	–0.32	–0.05	51	16000
Conformity 3 and Conformity 4	–0.16	0.14	–0.42	–0.16	0.12	51	16000

Supplementary Table S3: Numerical values of estimated parameter in Model 1 using both pathogen stress and government effectiveness as independent variables. GE and PS represent government effectiveness and pathogen stress, respectively. *ESS* represents effective sample sizes of MCMC simulations. Rows shaded in gray indicate the effects of slopes in which 95% Bayesian credible intervals of values did not include zero.

			Quantiles			
	Mean	*SD*	2.5%	50%	97.5%	*ESS*
Individualism						
a_0	–0.22	0.07	–0.37	–0.22	–0.08	13857
GE	0.44	0.10	0.25	0.44	0.63	10736
PS	–0.39	0.10	–0.58	–0.39	–0.19	11323
σ_y	0.69	0.05	0.60	0.69	0.80	13446
Conformity 1						
a_0	0.19	0.08	0.04	0.19	0.34	16000
GE	–0.31	0.10	–0.50	–0.31	–0.12	12934
PS	0.55	0.10	0.36	0.55	0.74	13420
σ_y	0.68	0.05	0.58	0.67	0.79	14347
Conformity 2						
a_0	0.13	0.10	–0.06	0.13	0.33	14358
GE	–0.20	0.12	–0.45	–0.20	0.04	11178
PS	0.43	0.12	0.20	0.43	0.67	11891
σ_y	0.84	0.07	0.72	0.84	0.99	14594
Conformity 3						
a_0	0.19	0.10	0.002	0.19	0.39	15191
GE	–0.25	0.11	–0.47	–0.25	–0.02	11100
PS	0.36	0.12	0.13	0.36	0.59	11223
σ_y	0.86	0.06	0.74	0.85	0.99	13363
Conformity 4						
a_0	–0.17	0.14	–0.45	–0.17	0.11	9963
GE	0.15	0.18	–0.21	0.15	0.51	7762
PS	–0.42	0.17	–0.76	–0.42	–0.09	8812
σ_y	0.88	0.09	0.72	0.87	1.09	11771

Supplementary Table S4: Numerical values of estimated parameter in Model 2 using both pathogen stress and government effectiveness as independent variables. GE and PS represent government effectiveness and pathogen stress, respectively. Subscript numbers under *a* represent global regions (1: Sub-Saharan Africa, 2 East Asia and Pacific, 3: Europe and Central Asia, 4: Latin America and Caribbean, 5: Middle East and North Africa, and 6: South Asia). *ESS* represents effective sample sizes of MCMC simulations. Rows shaded in gray indicate the effects of slopes in which 95% Bayesian credible intervals of values did not include zero.

			Quantiles			
	Mean	*SD*	2.5%	50%	97.5%	*ESS*
Individualism						
μ_a	–0.29	0.29	–0.87	–0.29	0.29	8551
GE	0.54	0.09	0.35	0.54	0.72	16000
PS	–0.11	0.12	–0.35	–0.11	0.13	10850
a_1	–0.20	0.18	–0.55	–0.19	0.16	16000
a_2	–0.93	0.20	–1.31	–0.93	–0.52	12602
a_3	0.14	0.15	–0.16	0.14	0.45	14116
a_4	–0.64	0.15	–0.93	–0.64	–0.36	16000
a_5	–0.06	0.16	–0.38	–0.06	0.26	16000
a_6	–0.10	0.23	–0.54	–0.09	0.36	16000
σ_a	0.61	0.33	0.25	0.53	1.44	7018
σ_y	0.60	0.05	0.52	0.60	0.70	16000
Conformity 1						
μ_a	0.28	0.21	–0.09	0.27	0.72	5287
GE	–0.30	0.10	–0.49	–0.30	–0.10	11251
PS	0.43	0.14	0.14	0.43	0.70	7365
a_1	0.26	0.20	–0.11	0.25	0.66	10565
a_2	0.17	0.19	–0.21	0.17	0.55	12158
a_3	–0.03	0.16	–0.36	–0.03	0.26	6655
a_4	0.44	0.18	0.10	0.44	0.80	7078
a_5	0.51	0.23	0.09	0.50	0.99	7103
a_6	0.35	0.27	–0.15	0.33	0.94	9989
σ_a	0.37	0.26	0.08	0.31	1.02	3637
σ_y	0.65	0.05	0.55	0.65	0.77	11364

Supplementary Table S4 (cond.): Numerical values of estimated parameters in Model 2 using both pathogen stress and government effectiveness as independent variables. GE and PS represent government effectiveness and pathogen stress, respectively. Subscript numbers under *a* represent global regions (1: Sub-Saharan Africa, 2 East Asia and Pacific, 3: Europe and Central Asia, 4: Latin America and Caribbean, 5: Middle East and North Africa, and 6: South Asia). *ESS* represents effective sample sizes of MCMC simulations. Rows shaded in gray indicate the effects of slopes in which 95% Bayesian credible intervals of values did not include zero.

			Quantiles			
	Mean	*SD*	2.5%	50%	97.5%	*ESS*
Conformity 2						
μ_a	0.11	0.32	–0.54	0.11	0.74	9305
GE	–0.17	0.13	–0.42	–0.17	0.08	5108
PS	0.58	0.18	0.25	0.58	0.94	7998
a_1	–0.13	0.27	–0.66	–0.12	0.36	3075
a_2	0.15	0.25	–0.36	0.15	0.65	12278
a_3	0.31	0.18	–0.05	0.31	0.68	10656
a_4	–0.33	0.25	–0.82	–0.34	0.17	1164
a_5	0.71	0.33	0.12	0.71	1.37	4165
a_6	–0.04	0.37	–0.79	–0.02	0.66	16000
σ_a	0.62	0.40	0.14	0.53	1.62	3274
σ_y	0.79	0.07	0.67	0.78	0.96	616
Conformity 3						
μ_a	0.27	0.43	–0.59	0.27	1.16	9214
GE	–0.14	0.11	–0.35	–0.14	0.07	13657
PS	0.12	0.15	–0.17	0.12	0.42	10529
a_1	1.16	0.27	0.65	1.16	1.69	12857
a_2	–0.42	0.25	–0.91	–0.43	0.06	13922
a_3	–0.27	0.17	–0.60	–0.27	0.05	12949
a_4	0.60	0.20	0.19	0.60	0.99	16000
a_5	0.56	0.23	0.10	0.56	1.02	16000
a_6	–0.01	0.38	–0.75	–0.01	0.72	16000
σ_a	0.91	0.49	0.38	0.79	2.15	7100
σ_y	0.73	0.06	0.62	0.72	0.85	16000

Supplementary Table S4 (cond.): Numerical values of estimated parameters in Model 2 using both pathogen stress and government effectiveness as independent variables. GE and PS represent government effectiveness and pathogen stress, respectively. Subscript numbers under *a* represent global regions (1: Sub-Saharan Africa, 2 East Asia and Pacific, 3: Europe and Central Asia, 4: Latin America and Caribbean, 5: Middle East and North Africa, and 6: South Asia). *ESS* represents effective sample sizes of MCMC simulations. Rows shaded in gray indicate the effects of slopes in which 95% Bayesian credible intervals of values did not include zero.

			Quantiles			
	Mean	*SD*	2.5%	50%	97.5%	*ESS*
Conformity 4						
μ_a	–0.39	0.45	–1.39	–0.34	0.34	5231
GE	0.27	0.18	–0.10	0.27	0.62	6319
PS	–0.16	0.25	–0.63	–0.16	0.35	3443
a_1	–0.37	0.34	–1.08	–0.35	0.25	5874
a_2	–0.71	0.37	–1.44	–0.72	–0.03	3103
a_3	0.03	0.25	–0.43	0.02	0.54	4397
a_4	0.12	0.30	–0.43	0.11	0.73	5725
a_5	–0.35	0.39	–1.17	–0.33	0.41	9420
a_6	–1.06	0.79	–2.83	–0.95	0.12	3078
σ_a	0.75	0.57	0.07	0.63	2.17	2689
σ_y	0.82	0.09	0.66	0.81	1.03	6541

Supplementary Table S5: Numerical values of estimated parameters in Model 3 using both pathogen stress and government effectiveness as independent variables. GE and PS represent government effectiveness and pathogen stress, respectively. Subscript numbers under *a*, GE, and PS represent global regions (1: Sub-Saharan Africa, 2 East Asia and Pacific, 3: Europe and Central Asia, 4: Latin America and Caribbean, 5: Middle East and North Africa, and 6: South Asia). *ESS* represents effective sample sizes of MCMC simulations. Rows shaded in gray indicate the effects of slopes in which 95% Bayesian credible intervals of values did not include zero.

			Quantiles			
	Mean	*SD*	2.5%	50%	97.5%	*ESS*
Individualism						
μ_a	–0.38	0.26	–0.89	–0.38	0.13	6822
μ_{GE}	0.48	0.20	0.09	0.47	0.87	6604
μ_{PS}	0.07	0.27	–0.41	0.05	0.64	6929
a_1	–0.18	0.24	–0.64	–0.18	0.33	8870
a_2	–0.84	0.25	–1.34	–0.83	–0.37	5778
a_3	–0.17	0.18	–0.51	–0.17	0.18	10043
a_4	–0.70	0.15	–0.99	–0.70	–0.42	10297
a_5	–0.10	0.15	–0.39	–0.10	0.20	12569
a_6	–0.25	0.27	–0.78	–0.25	0.29	7768
GE_1	0.45	0.24	–0.03	0.46	0.93	10631
GE_2	0.25	0.20	–0.15	0.25	0.60	5397
GE_3	0.66	0.12	0.42	0.66	0.91	5398
GE_4	0.49	0.22	0.07	0.49	0.94	9425
GE_5	0.36	0.18	–0.002	0.37	0.69	9464
GE_6	0.64	0.34	0.08	0.59	1.45	7004
PS_1	–0.19	0.25	–0.71	–0.17	0.27	8240
PS_2	0.07	0.29	–0.47	0.06	0.66	8117
PS_3	–0.27	0.17	–0.60	–0.27	0.05	5718
PS_4	0.31	0.31	–0.23	0.30	0.95	6336
PS_5	0.19	0.26	–0.30	0.18	0.73	8485
PS_6	0.29	0.36	–0.32	0.26	1.05	6483
σ_a	0.51	0.30	0.16	0.44	1.27	5450
σ_{GE}	0.33	0.26	0.04	0.28	0.96	3146
σ_{PS}	0.46	0.32	0.07	0.40	1.28	4227
σ_y	0.56	0.04	0.48	0.56	0.65	16000

Supplementary Table S5 (cond.): Numerical values of estimated parameters in Model 3 using both pathogen stress and government effectiveness as independent variables. GE and PS represent government effectiveness and pathogen stress, respectively. Subscript numbers under *a*, GE, and PS represent global regions (1: Sub-Saharan Africa, 2 East Asia and Pacific, 3: Europe and Central Asia, 4: Latin America and Caribbean, 5: Middle East and North Africa, and 6: South Asia). *ESS* represents effective sample sizes of MCMC simulations. Rows shaded in gray indicate the effects of slopes in which 95% Bayesian credible intervals of values did not include zero.

			Quantiles			
	Mean	*SD*	2.5%	50%	97.5%	*ESS*
Conformity 1						
μ_a	0.37	0.22	–0.10	0.36	0.82	953
μ_{GE}	–0.27	0.17	–0.59	–0.27	0.08	5800
μ_{PS}	0.33	0.24	–0.19	0.35	0.77	4562
a_1	0.40	0.26	–0.07	0.39	0.96	5750
a_2	0.25	0.23	–0.21	0.26	0.71	4975
a_3	0.10	0.19	–0.29	0.10	0.47	1382
a_4	0.48	0.18	0.14	0.47	0.83	6079
a_5	0.57	0.25	0.14	0.55	1.11	3353
a_6	0.44	0.29	–0.09	0.42	1.10	5386
GE_1	–0.22	0.22	–0.61	–0.24	0.31	5848
GE_2	–0.29	0.16	–0.63	–0.29	0.03	7327
GE_3	–0.27	0.11	–0.50	–0.27	–0.05	6332
GE_4	–0.28	0.20	–0.69	–0.28	0.13	7253
GE_5	–0.24	0.21	–0.64	–0.26	0.24	5460
GE_6	–0.29	0.28	–0.93	–0.29	0.26	6773
PS_1	0.31	0.24	–0.20	0.32	0.74	4450
PS_2	0.26	0.29	–0.38	0.29	0.78	4557
PS_3	0.57	0.19	0.21	0.57	0.96	1976
PS_4	0.26	0.29	–0.39	0.29	0.76	4466
PS_5	0.31	0.34	–0.46	0.34	0.95	4719
PS_6	0.27	0.33	–0.50	0.31	0.83	4412
σ_a	0.35	0.26	0.03	0.30	0.97	1821
σ_{GE}	0.19	0.20	0.01	0.13	0.71	1841
σ_{PS}	0.32	0.27	0.02	0.27	1.00	1933
σ_y	0.65	0.05	0.55	0.65	0.77	7140

Supplementary Table 5 (cond.): Numerical values of estimated parameter in Model 3 using both pathogen stress and government effectiveness as independent variables. GE and PS represent government effectiveness and pathogen stress, respectively. Subscript numbers under *a*, GE, and PS represent global regions (1: Sub-Saharan Africa, 2 East Asia and Pacific, 3: Europe and Central Asia, 4: Latin America and Caribbean, 5: Middle East and North Africa, and 6: South Asia). *ESS* represents effective sample sizes of MCMC simulations. Rows shaded in gray indicate the effects of slopes in which 95% Bayesian credible intervals of values did not include zero.

			Quantiles			
	Mean	*SD*	2.5%	50%	97.5%	*ESS*
Conformity 2						
μ_a	0.08	0.37	–0.67	0.09	0.78	4666
μ_{GE}	–0.17	0.26	–0.67	–0.17	0.36	5703
μ_{PS}	0.58	0.27	0.08	0.59	1.10	1551
a_1	–0.29	0.39	–1.13	–0.26	0.39	3265
a_2	0.15	0.31	–0.47	0.15	0.79	3437
a_3	0.30	0.21	–0.12	0.30	0.71	3348
a_4	–0.38	0.25	–0.87	–0.38	0.12	2774
a_5	0.76	0.36	0.08	0.75	1.46	3661
a_6	–0.05	0.42	–0.91	–0.04	0.75	3457
GE_1	–0.34	0.36	–1.30	–0.28	0.22	941
GE_2	–0.11	0.22	–0.52	–0.12	0.37	4179
GE_3	–0.19	0.14	–0.46	–0.18	0.09	4063
GE_4	–0.11	0.28	–0.63	–0.12	0.50	4815
GE_5	–0.13	0.43	–1.04	–0.15	0.89	797
GE_6	–0.14	0.41	–0.97	–0.15	0.76	7342
PS_1	0.65	0.30	0.10	0.64	1.33	2069
PS_2	0.47	0.34	–0.32	0.50	1.07	2373
PS_3	0.56	0.21	0.14	0.56	0.96	1813
PS_4	0.66	0.32	0.08	0.64	1.35	1729
PS_5	0.59	0.37	–0.17	0.59	1.35	2362
PS_6	0.58	0.34	–0.12	0.58	1.28	2852
σ_a	0.69	0.46	0.16	0.60	1.78	2550
σ_{GE}	0.33	0.33	0.03	0.23	1.16	1344
σ_{PS}	0.31	0.29	0.03	0.23	1.06	1589
σ_y	0.79	0.07	0.67	0.78	0.93	7190

Supplementary Table S5 (cond.): Numerical values of estimated parameter in Model 3 using both pathogen stress and government effectiveness as independent variables. GE and PS represent government effectiveness and pathogen stress, respectively. Subscript numbers under *a*, GE, and PS represent global regions (1: Sub-Saharan Africa, 2 East Asia and Pacific, 3: Europe and Central Asia, 4: Latin America and Caribbean, 5: Middle East and North Africa, and 6: South Asia). *ESS* represents effective sample sizes of MCMC simulations. Rows shaded in gray indicate the effects of slopes in which 95% Bayesian credible intervals of values did not include zero.

			Quantiles			
	Mean	*SD*	2.5%	50%	97.5%	*ESS*
Conformity 3						
μ_a	0.21	0.38	–0.53	0.21	0.95	4414
μ_{GE}	–0.17	0.39	–0.83	–0.20	0.69	4868
μ_{PS}	0.21	0.29	–0.35	0.21	0.77	5613
a_1	0.71	0.41	–0.10	0.72	1.50	4360
a_2	–0.23	0.33	–0.87	–0.23	0.39	4917
a_3	–0.35	0.18	–0.71	–0.36	0.004	4583
a_4	0.58	0.20	0.19	0.58	0.97	5160
a_5	0.47	0.23	0.04	0.47	0.92	3679
a_6	0.10	0.46	–0.77	0.08	1.08	4360
GE_1	–0.15	0.38	–0.88	–0.17	0.66	3778
GE_2	–0.47	0.25	–0.97	–0.46	0.02	3841
GE_3	–0.03	0.12	–0.26	–0.02	0.22	2877
GE_4	–0.07	0.32	–0.67	–0.08	0.59	6056
GE_5	–0.64	0.28	–1.22	–0.63	–0.13	3709
GE_6	0.36	0.81	–0.74	0.16	2.45	3291
PS_1	0.55	0.38	–0.08	0.50	1.38	3004
PS_2	0.16	0.34	–0.54	0.16	0.83	6592
PS_3	0.13	0.18	–0.21	0.13	0.48	3502
PS_4	0.07	0.33	–0.65	0.10	0.69	5464
PS_5	0.13	0.32	–0.56	0.14	0.73	6627
PS_6	0.23	0.36	–0.51	0.22	0.99	8003
σ_a	0.73	0.43	0.25	0.63	1.78	3189
σ_{GE}	0.65	0.56	0.12	0.51	2.07	3254
σ_{PS}	0.42	0.37	0.06	0.33	1.29	2123
σ_y	0.69	0.06	0.59	0.69	0.82	1670

Supplementary Table S5 (cond.): Numerical values of estimated parameter in Model 3 using both pathogen stress and government effectiveness as independent variables. GE and PS represent government effectiveness and pathogen stress, respectively. Subscript numbers under *a*, GE, and PS represent global regions (1: Sub-Saharan Africa, 2 East Asia and Pacific, 3: Europe and Central Asia, 4: Latin America and Caribbean, 5: Middle East and North Africa, and 6: South Asia). *ESS* represents effective sample sizes of MCMC simulations. Rows shaded in gray indicate the effects of slopes in which 95% Bayesian credible intervals of values did not include zero.

			Quantiles			
	Mean	*SD*	2.5%	50%	97.5%	*ESS*
Conformity 4						
μ_a	–0.29	0.47	–1.36	–0.24	0.49	4546
μ_{GE}	0.30	0.51	–0.63	0.29	1.27	6272
μ_{PS}	–0.34	0.52	–1.53	–0.29	0.53	3730
a_1	–0.42	0.47	–1.47	–0.37	0.38	4220
a_2	–0.61	0.48	–1.58	–0.59	0.24	2711
a_3	0.07	0.27	–0.44	0.06	0.62	6340
a_4	0.16	0.31	–0.42	0.15	0.79	5494
a_5	–0.19	0.45	–1.10	–0.18	0.72	4996
a_6	–0.75	0.87	–2.76	–0.58	0.57	2963
GE_1	0.50	0.48	–0.32	0.43	1.64	4225
GE_2	0.29	0.36	–0.45	0.28	1.03	5584
GE_3	0.27	0.20	–0.13	0.27	0.64	6923
GE_4	0.19	0.47	–0.88	0.22	1.09	7413
GE_5	0.21	0.80	–1.70	0.26	1.68	6341
GE_6	0.36	1.05	–1.38	0.30	2.44	4022
PS_1	0.04	0.46	–0.76	0.00	1.07	3515
PS_2	–0.37	0.51	–1.47	–0.34	0.57	3217
PS_3	–0.12	0.29	–0.68	–0.12	0.47	5308
PS_4	–0.15	0.42	–0.99	–0.15	0.70	7933
PS_5	–0.69	0.88	–2.88	–0.49	0.57	2991
PS_6	–0.71	0.77	–2.46	–0.57	0.49	2630
σ_a	0.72	0.61	0.05	0.58	2.27	2350
σ_{GE}	0.57	0.74	0.03	0.36	2.34	1372
σ_{PS}	0.69	0.69	0.04	0.51	2.39	2082
σ_y	0.82	0.10	0.66	0.81	1.03	9234

Supplementary Tables S6: Summary of analysis results using both pathogen stress and government effectiveness as independent variables. Parameters in parentheses indicate the parameters in Model 3. Asterisks in columns of global effects (GE, PS, μ_{GE}, or μ_{PS}) indicate that the effect was significant (i.e., the 95% Bayesian credible interval of the effect did not include zero). Numbers in columns of region–specific effects (GE_j or PS_j) indicate global regions in which significant effects were found (1 = Sub-Saharan Africa, 2 = East Asia and Pacific, 3 = Europe and Central Asia, 4 = Latin America and Caribbean, 5 = Middle East and North Africa, and 6 = South Asia). Rows shaded in gray indicate the best model, in which the WAIC value was smallest in three models.

Dependent variables	Model	Parameters			
		GE (μ_{GE})	PS (μ_{PS})	(GE_j)	(PS_j)
Individualism	Model 1	*	*		
	Model 2	*			
	Model 3	*		3,4,6	
Conformity 1	Model 1	*	*		
	Model 2	*	*		
	Model 3			3	3
Conformity 2	Model 1		*		
	Model 2		*		
	Model 3		*		1, 3, 4
Conformity 3	Model 1	*	*		
	Model 2				
	Model 3			5	
Conformity 4	Model 1		*		
	Model 2				
	Model 3				

Supplementary Table S7: Numerical values of estimated parameters in Model 1 using both pathogen stress and GDP per capita as independent variables. GDP and PS represent GDP per capita and pathogen stress, respectively. *ESS* represents effective sample sizes of MCMC simulations. Rows shaded in gray indicate the effects of slopes in which 95% Bayesian credible intervals of values did not include zero.

			Quantiles			
	Mean	*SD*	2.5%	50%	97.5%	*ESS*
Individualism						
a_0	–0.09	0.07	–0.24	–0.09	0.05	15682
GDP	0.40	0.11	0.18	0.40	0.62	12681
PS	–0.46	0.10	–0.66	–0.46	–0.27	12240
σ_y	0.72	0.05	0.63	0.72	0.83	13678
Conformity 1						
a_0	0.09	0.08	–0.06	0.09	0.25	12862
GDP	–0.45	0.12	–0.69	–0.45	–0.22	13024
PS	0.54	0.09	0.36	0.54	0.72	12445
σ_y	0.66	0.05	0.57	0.66	0.78	13877
Conformity 2						
a_0	0.06	0.10	–0.14	0.06	0.26	13980
GDP	–0.27	0.15	–0.57	–0.27	0.03	11467
PS	0.43	0.12	0.20	0.43	0.67	11973
σ_y	0.84	0.07	0.72	0.84	0.99	13963
Conformity 3						
a_0	0.14	0.10	–0.05	0.14	0.33	14599
GDP	–0.20	0.14	–0.47	–0.20	0.07	12626
PS	0.43	0.12	0.20	0.43	0.65	12442
σ_y	0.87	0.07	0.75	0.86	1.00	15600
Conformity 4						
a_0	–0.12	0.13	–0.38	–0.12	0.14	13741
GDP	0.24	0.20	–0.16	0.23	0.63	10677
PS	–0.38	0.17	–0.71	–0.39	–0.05	10524
σ_y	0.88	0.09	0.72	0.87	1.08	13303

Supplementary Table S8: Numerical values of estimated parameters in Model 2 using both pathogen stress and GDP per capita as independent variables. GDP and PS represent GDP per capita and pathogen stress, respectively. Subscript numbers under *a* represent global regions (1: Sub-Saharan Africa, 2 East Asia and Pacific, 3: Europe and Central Asia, 4: Latin America and Caribbean, 5: Middle East and North Africa, and 6: South Asia). *ESS* represents effective sample sizes of MCMC simulations. Rows shaded in gray indicate the effects of slopes in which 95% Bayesian credible intervals of values did not include zero.

			Quantiles			
	Mean	*SD*	2.5%	50%	97.5%	*ESS*
Individualism						
μ_a	–0.14	0.23	–0.61	–0.14	0.30	7079
GDP	0.39	0.11	0.18	0.39	0.61	1914
PS	–0.28	0.13	–0.53	–0.28	–0.02	9514
a_1	–0.10	0.19	–0.47	–0.09	0.26	16000
a_2	–0.47	0.21	–0.89	–0.47	–0.03	643
a_3	0.26	0.17	–0.06	0.26	0.60	2562
a_4	–0.45	0.17	–0.78	–0.45	–0.10	512
a_5	–0.12	0.17	–0.45	–0.12	0.20	16000
a_6	0.02	0.23	–0.43	0.02	0.49	16000
σ_a	0.45	0.28	0.12	0.39	1.15	1414
σ_y	0.67	0.05	0.58	0.67	0.78	2058
Conformity 1						
μ_a	0.19	0.21	–0.19	0.18	0.63	7054
GDP	–0.43	0.12	–0.66	–0.43	–0.19	11358
PS	0.43	0.13	0.17	0.44	0.68	8317
a_1	0.16	0.19	–0.21	0.16	0.55	11193
a_2	0.03	0.18	–0.34	0.04	0.37	14129
a_3	–0.10	0.15	–0.41	–0.10	0.19	5874
a_4	0.32	0.17	0.004	0.31	0.67	9951
a_5	0.47	0.24	0.05	0.46	0.95	5687
a_6	0.24	0.27	–0.27	0.22	0.82	12130
σ_a	0.37	0.25	0.09	0.32	0.99	4106
σ_y	0.63	0.05	0.54	0.63	0.75	8258

Supplementary Table S8 (cond.): Numerical values of estimated parameters in Model 2 using both pathogen stress and GDP per capita as independent variables. GDP and PS represent GDP per capita and pathogen stress, respectively. Subscript numbers under *a* represent global regions (1: Sub-Saharan Africa, 2 East Asia and Pacific, 3: Europe and Central Asia, 4: Latin America and Caribbean, 5: Middle East and North Africa, 6: South Asia). *ESS* represents effective sample sizes of MCMC simulations. Rows shaded in gray indicate the effects of slopes in which 95% Bayesian credible intervals of values did not include zero.

			Quantiles			
	Mean	*SD*	2.5%	50%	97.5%	*ESS*
Conformity 2						
μ_a	0.03	0.32	–0.62	0.04	0.66	10060
GDP	–0.24	0.15	–0.53	–0.24	0.05	13007
PS	0.60	0.17	0.28	0.59	0.92	8913
a_1	–0.20	0.26	–0.73	–0.19	0.31	11423
a_2	0.01	0.23	–0.46	0.02	0.47	16000
a_3	0.28	0.19	–0.08	0.28	0.65	9830
a_4	–0.42	0.24	–0.90	–0.42	0.05	8519
a_5	0.67	0.33	0.06	0.67	1.32	8361
a_6	–0.13	0.37	–0.89	–0.11	0.57	16000
σ_a	0.63	0.38	0.16	0.54	1.60	5735
σ_y	0.78	0.07	0.67	0.78	0.93	13591
Conformity 3						
μ_a	0.23	0.45	–0.66	0.22	1.13	6600
GDP	–0.13	0.12	–0.37	–0.13	0.11	16000
PS	0.15	0.15	–0.13	0.15	0.45	9677
a_1	1.12	0.26	0.61	1.12	1.64	11825
a_2	–0.49	0.24	–0.95	–0.48	–0.03	16000
a_3	–0.29	0.17	–0.63	–0.29	0.04	11100
a_4	0.55	0.21	0.14	0.55	0.95	16000
a_5	0.54	0.24	0.08	0.54	1.01	16000
a_6	–0.05	0.38	–0.81	–0.05	0.70	16000
σ_a	0.92	0.51	0.38	0.80	2.23	6105
σ_y	0.73	0.06	0.63	0.73	0.86	16000

Supplementary Table S8 (cond.): Numerical values of estimated parameters in Model 2 using both pathogen stress and GDP per capita as independent variables. GDP and PS represent GDP per capita and pathogen stress, respectively. Subscript numbers under *a* represent global regions (1: Sub-Saharan Africa, 2 East Asia and Pacific, 3: Europe and Central Asia, 4: Latin America and Caribbean, 5: Middle East and North Africa, 6: South Asia). *ESS* represents effective sample sizes of MCMC simulations. Rows shaded in gray indicate the effects of slopes in which 95% Bayesian credible intervals of values did not include zero.

			Quantiles			
	Mean	*SD*	2.5%	50%	97.5%	*ESS*
Conformity 4						
μ_a	–0.29	0.42	–1.24	–0.25	0.41	3428
GDP	0.30	0.20	–0.09	0.30	0.68	311
PS	–0.19	0.24	–0.65	–0.19	0.30	293
a_1	–0.28	0.32	–0.96	–0.26	0.32	5074
a_2	–0.61	0.35	–1.31	–0.60	0.02	797
a_3	0.07	0.25	–0.37	0.06	0.58	730
a_4	0.25	0.31	–0.30	0.24	0.86	344
a_5	–0.22	0.37	–1.00	–0.21	0.52	12982
a_6	–0.89	0.73	–2.56	–0.76	0.19	1674
σ_a	0.71	0.56	0.07	0.59	2.11	558
σ_y	0.82	0.09	0.66	0.81	1.03	2414

Supplementary Table S9: Numerical values of estimated parameters in Model 3 using both pathogen stress and GDP per capita as independent variables. GDP and PS represent GDP per capita and pathogen stress, respectively. Subscript numbers under *a*, GDP, and PS represent global regions (1: Sub-Saharan Africa, 2 East Asia and Pacific, 3: Europe and Central Asia, 4: Latin America and Caribbean, 5: Middle East and North Africa, and 6: South Asia). *ESS* represents effective sample sizes of MCMC simulations. Rows shaded in gray indicate the effects of slopes when 95% Bayesian credible intervals of values did not include zero.

			Quantiles			
	Mean	*SD*	2.5%	50%	97.5%	*ESS*
Individualism						
μ_a	–0.26	0.23	–0.72	–0.26	0.22	5700
μ_{GDP}	0.32	0.28	–0.26	0.32	0.86	6195
μ_{PS}	–0.10	0.30	–0.64	–0.11	0.55	6511
a_1	–0.08	0.31	–0.61	–0.10	0.60	5328
a_2	–0.58	0.24	–1.06	–0.57	–0.15	5118
a_3	–0.01	0.20	–0.38	–0.01	0.38	4976
a_4	–0.49	0.19	–0.86	–0.50	–0.13	5076
a_5	–0.15	0.16	–0.46	–0.15	0.18	12622
a_6	–0.22	0.32	–0.83	–0.22	0.44	4866
GDP_1	0.32	0.45	–0.61	0.32	1.26	6667
GDP_2	0.32	0.21	–0.12	0.33	0.74	12025
GDP_3	0.43	0.12	0.20	0.43	0.67	7035
GDP_4	0.46	0.39	–0.24	0.42	1.40	6822
GDP_5	0.07	0.27	–0.50	0.08	0.52	3223
GDP_6	0.31	0.44	–0.61	0.32	1.21	6522
PS_1	–0.37	0.25	–0.90	–0.36	0.09	6937
PS_2	–0.04	0.33	–0.65	–0.06	0.63	4154
PS_3	–0.50	0.17	–0.83	–0.50	–0.16	4310
PS_4	0.06	0.31	–0.49	0.05	0.70	6581
PS_5	0.05	0.33	–0.56	0.03	0.73	3267
PS_6	0.24	0.42	–0.47	0.21	1.13	5172
σ_a	0.42	0.28	0.07	0.36	1.12	4165
σ_{GDP}	0.40	0.37	0.03	0.31	1.36	2344
σ_{PS}	0.53	0.37	0.07	0.45	1.41	2182
σ_y	0.63	0.05	0.54	0.63	0.74	6259

Supplementary Table S9 (cond.): Numerical values of estimated parameters in Model 3 using both pathogen stress and GDP per capita as independent variables. GDP and PS represent GDP per capita and pathogen stress, respectively. Subscript numbers under *a*, GDP, and PS represent global regions (1: Sub-Saharan Africa, 2 East Asia and Pacific, 3: Europe and Central Asia, 4: Latin America and Caribbean, 5: Middle East and North Africa, 6: South Asia). *ESS* represents effective sample sizes of MCMC simulations. Rows shaded in gray indicate the effects of slopes when 95% Bayesian credible intervals of values did not include zero.

			Quantiles			
	Mean	*SD*	2.5%	50%	97.5%	*ESS*
Conformity 1						
μ_a	0.28	0.22	–0.13	0.27	0.73	4268
μ_{GDP}	–0.47	0.30	–1.11	–0.46	0.11	4620
μ_{PS}	0.30	0.26	–0.27	0.33	0.75	4055
a_1	0.29	0.29	–0.24	0.27	0.91	3831
a_2	0.17	0.22	–0.26	0.17	0.60	4998
a_3	0.04	0.19	–0.34	0.04	0.40	2316
a_4	0.36	0.19	0.001	0.35	0.75	4749
a_5	0.49	0.27	0.03	0.47	1.08	2803
a_6	0.33	0.32	–0.26	0.31	1.03	5112
GDP_1	–0.45	0.44	–1.39	–0.44	0.47	5515
GDP_2	–0.58	0.24	–1.07	–0.56	–0.16	2545
GDP_3	–0.36	0.13	–0.62	–0.35	–0.09	5804
GDP_4	–0.42	0.39	–1.20	–0.43	0.41	3867
GDP_5	–0.53	0.44	–1.51	–0.49	0.34	4718
GDP_6	–0.51	0.45	–1.50	–0.47	0.37	4194
PS_1	0.29	0.25	–0.23	0.30	0.73	4438
PS_2	0.16	0.33	–0.58	0.20	0.69	2562
PS_3	0.59	0.18	0.25	0.58	0.94	2377
PS_4	0.29	0.27	–0.29	0.31	0.79	6891
PS_5	0.28	0.35	–0.51	0.32	0.92	6164
PS_6	0.20	0.35	–0.61	0.26	0.79	3457
σ_a	0.34	0.28	0.02	0.28	1.02	2530
σ_{GDP}	0.37	0.35	0.03	0.28	1.30	1847
σ_{PS}	0.37	0.30	0.03	0.30	1.11	1764
σ_y	0.63	0.05	0.53	0.63	0.75	11556

Supplementary Table S9 (cond.): Numerical values of estimated parameters in Model 3 using both pathogen stress and GDP per capita as independent variables. GDP and PS represent GDP per capita and pathogen stress, respectively. Subscript numbers under *a*, GDP, and PS represent global regions (1: Sub-Saharan Africa, 2 East Asia and Pacific, 3: Europe and Central Asia, 4: Latin America and Caribbean, 5: Middle East and North Africa, and 6: South Asia). *ESS* represents effective sample sizes of MCMC simulations. Rows shaded in gray indicate the effects of slopes when 95% Bayesian credible intervals of values did not include zero.

			Quantiles			
	Mean	*SD*	2.5%	50%	97.5%	*ESS*
Conformity 2						
μ_a	0.04	0.35	–0.67	0.05	0.73	4806
μ_{GDP}	–0.21	0.40	–0.98	–0.23	0.66	667
μ_{PS}	0.60	0.27	0.08	0.58	1.14	1533
a_1	–0.27	0.44	–1.25	–0.27	0.52	3549
a_2	0.05	0.27	–0.48	0.03	0.60	2356
a_3	0.25	0.21	–0.15	0.24	0.69	283
a_4	–0.39	0.28	–0.94	–0.38	0.14	279
a_5	0.62	0.37	–0.06	0.59	1.39	1203
a_6	–0.08	0.46	–1.04	–0.06	0.86	4291
GDP_1	–0.14	0.61	–1.37	–0.18	1.24	3815
GDP_2	–0.13	0.28	–0.66	–0.16	0.46	2177
GDP_3	–0.28	0.16	–0.59	–0.28	0.05	1805
GDP_4	–0.06	0.53	–1.06	–0.12	1.27	3637
GDP_5	–0.45	0.61	–1.86	–0.36	0.62	159
GDP_6	–0.12	0.61	–1.31	–0.14	1.28	4112
PS_1	0.71	0.31	0.20	0.66	1.41	633
PS_2	0.49	0.34	–0.27	0.50	1.11	2107
PS_3	0.56	0.19	0.18	0.55	0.97	1603
PS_4	0.69	0.31	0.15	0.65	1.38	1173
PS_5	0.58	0.38	–0.22	0.56	1.36	2393
PS_6	0.58	0.35	–0.17	0.57	1.30	2213
σ_a	0.63	0.44	0.11	0.54	1.73	181
σ_{GDP}	0.58	0.55	0.07	0.40	1.86	79
σ_{PS}	0.31	0.33	0.03	0.22	1.11	275
σ_y	0.79	0.07	0.67	0.79	0.93	1738

Supplementary Table S9 (cond.): Numerical values of estimated parameters in Model 3 using both pathogen stress and GDP per capita as independent variables. GDP and PS represent GDP per capita and pathogen stress, respectively. Subscript numbers under *a*, GDP, and PS represent global regions (1: Sub-Saharan Africa, 2 East Asia and Pacific, 3: Europe and Central Asia, 4: Latin America and Caribbean, 5: Middle East and North Africa, and 6: South Asia). *ESS* represents effective sample sizes of MCMC simulations. Rows shaded in gray indicate the effects of slopes when 95% Bayesian credible intervals of values did not include zero.

			Quantiles			
	Mean	*SD*	2.5%	50%	97.5%	*ESS*
Conformity 3						
μ_a	0.02	0.39	–0.73	0.01	0.79	5471
μ_{GDP}	–0.56	0.55	–1.73	–0.53	0.40	6642
μ_{PS}	0.20	0.26	–0.29	0.19	0.72	6302
a_1	0.36	0.51	–0.63	0.35	1.38	5830
a_2	–0.44	0.27	–0.98	–0.44	0.08	9198
a_3	–0.38	0.18	–0.73	–0.37	–0.04	9299
a_4	0.47	0.26	–0.05	0.47	0.98	7928
a_5	0.31	0.26	–0.20	0.30	0.82	9293
a_6	–0.19	0.52	–1.26	–0.17	0.82	7889
GDP_1	–1.00	0.91	–3.05	–0.89	0.52	4704
GDP_2	–0.69	0.29	–1.27	–0.69	–0.13	9585
GDP_3	–0.04	0.13	–0.28	–0.04	0.21	11987
GDP_4	–0.31	0.61	–1.53	–0.32	0.95	7879
GDP_5	–1.01	0.57	–2.20	–0.98	–0.01	7017
GDP_6	–0.32	0.81	–1.96	–0.33	1.40	7502
PS_1	0.43	0.35	–0.12	0.38	1.22	3301
PS_2	0.11	0.33	–0.62	0.12	0.76	7805
PS_3	0.12	0.16	–0.20	0.12	0.43	8233
PS_4	0.09	0.28	–0.54	0.10	0.62	10325
PS_5	0.20	0.30	–0.39	0.19	0.84	9474
PS_6	0.21	0.35	–0.49	0.19	0.95	9390
σ_a	0.71	0.44	0.21	0.61	1.80	4464
σ_{GDP}	0.88	0.66	0.16	0.72	2.54	4001
σ_{PS}	0.35	0.31	0.02	0.27	1.15	2286
σ_y	0.70	0.06	0.60	0.69	0.82	16000

Supplementary Table S9 (cond.): Numerical values of estimated parameters in Model 3 using both pathogen stress and GDP per capita as independent variables. GDP and PS represent GDP per capita and pathogen stress, respectively. Subscript numbers under *a*, GDP, and PS represent global regions (1: Sub-Saharan Africa, 2 East Asia and Pacific, 3: Europe and Central Asia, 4: Latin America and Caribbean, 5: Middle East and North Africa, and 6: South Asia). *ESS* represents effective sample sizes of MCMC simulations. Rows shaded in gray indicate the effects of slopes when 95% Bayesian credible intervals of values did not include zero.

			Quantiles			
	Mean	*SD*	2.5%	50%	97.5%	*ESS*
Conformity 4						
μ_a	–0.16	0.57	–1.20	–0.11	0.67	2631
μ_{GDP}	0.41	0.68	–0.86	0.37	1.89	4987
μ_{PS}	–0.32	0.45	–1.32	–0.29	0.45	3228
a_1	–0.26	0.53	–1.38	–0.23	0.74	2023
a_2	–0.50	0.41	–1.33	–0.47	0.24	2997
a_3	0.14	0.27	–0.37	0.14	0.67	2761
a_4	0.23	0.37	–0.42	0.23	0.98	422
a_5	–0.06	0.55	–1.12	–0.07	1.06	2235
a_6	–0.52	0.85	–2.51	–0.36	0.79	2019
GDP_1	0.55	0.93	–1.05	0.42	2.84	4174
GDP_2	0.46	0.36	–0.23	0.44	1.22	4933
GDP_3	0.28	0.21	–0.13	0.28	0.70	5579
GDP_4	0.04	1.06	–2.69	0.21	1.82	168
GDP_5	0.28	0.99	–1.95	0.32	2.21	227
GDP_6	0.75	1.19	–1.03	0.52	3.81	3812
PS_1	–0.05	0.43	–0.83	–0.09	0.92	1860
PS_2	–0.32	0.46	–1.30	–0.31	0.58	3443
PS_3	–0.13	0.27	–0.65	–0.14	0.41	3215
PS_4	–0.20	0.38	–0.95	–0.21	0.55	5786
PS_5	–0.60	0.80	–2.67	–0.40	0.53	3223
PS_6	–0.58	0.71	–2.29	–0.42	0.52	2831
σ_a	0.72	0.69	0.05	0.59	2.21	1952
σ_{GDP}	0.83	0.90	0.04	0.55	3.15	603
σ_{PS}	0.56	0.56	0.01	0.41	2.03	857
σ_y	0.82	0.09	0.66	0.81	1.02	6635

Supplementary Tables S10: Summary of analysis results using both pathogen stress and GDP as independent variables. Parameters in parentheses indicate the parameters in Model 3. Asterisks in columns of global effects (GDP, PS, μ_{GE}, or μ_{PS}) indicate that the effect was significant (i.e., the 95% Bayesian credible interval of the effect did not include zero). Numbers in columns of region–specific effects (GDP_j or PS_j) indicate global regions in which significant effects were found (1 = Sub-Saharan Africa, 2 = East Asia and Pacific, 3 = Europe and Central Asia, 4 = Latin America and Caribbean, 5 = Middle East and North Africa, and 6 = South Asia). Rows shaded in gray indicate the best model, in which the WAIC value was smallest in three models.

Dependent variables	Model	Parameters			
		GDP (μ_{GDP})	PS (μ_{PS})	(GDP_j)	(PS_j)
Individualism	Model 1	*	*		
	Model 2	*	*		
	Model 3			3	3
Conformity 1	Model 1	*	*		
	Model 2	*	*		
	Model 3			2, 3	3
Conformity 2	Model 1		*		
	Model 2		*		
	Model 3		*		1, 3, 4
Conformity 3	Model 1		*		
	Model 2				
	Model 3			2, 5	
Conformity 4	Model 1		*		
	Model 2				
	Model 3				

Supplementary Method

Bayesian estimation of zero-order correlation coefficients

Here, we explain the method of Bayesian estimation of zero-order correlation coefficients between y_a and y_b. $\vec{y}$ represents the two–dimensional vector of y, and $\vec{y}$ obeys the following statistical model:

$$\vec{\mu} = \begin{bmatrix} \mu_a \\ \mu_b \end{bmatrix},$$

$$\Sigma = \begin{pmatrix} \sigma_a^2 & \sigma_a\sigma_b\rho \\ \sigma_a\sigma_b\rho & \sigma_b^2 \end{pmatrix}, \qquad \text{(S1)}$$

$$\vec{y} \sim \text{Normal}_2(\vec{\mu}, \Sigma).$$

$\vec{\mu}$ represents the mean vector of y. μ_a and μ_b represent the mean values of y_a and y_b, respectively. Σ represents the covariance matrix of y_a and y_b. σ_a and σ_b represent the standard deviation of y_a and y_b, respectively. σ_{ab} represents the covariance of y_a and y_b. ρ represents the correlation coefficients between y_a and y_b. $\vec{y}$ obeys two variants normal distribution with two–dimensional mean vector $\vec{\mu}$ and 2 x 2 covariance matrix Σ.

We estimated the parameter values of $\vec{\mu}$, Σ, and ρ from y_a and y_b by conducting MCMC simulations. We used the uniformed distribution [–1, 1] for a prior of ρ. We calculated the 95% Bayesian credible intervals of ρ.
