# Supplementary material for: Cultural Differences in Strength of Conformity Explained Through Pathogen Stress: A Statistical Test Using Hierarchical Bayesian Estimation
Source: Front Psychol. 2018 Oct 11;9:1921. doi: 10.3389/fpsyg.2018.01921 (PMC6193438; doi:10.3389/fpsyg.2018.01921)
Supplement: Supplementary file 2 [file Data_Sheet_2.PDF]

Supplementary material for "Cultural differences in strength of conformity explained through pathogen stress: A statistical test using hierarchical Bayesian estimation"

Yutaka Horita & Masanori Takezawa

Supplementary Analysis

Other measures representing individualistic/collectivistic values

Summary of Supplementary Analysis

Supplementary Figures

Supplementary Figure S16–S24: Scatter plots displaying the correlations between regional level of government effectiveness (pathogen stress or GDP per capita) and each index of individualism/collectivism by each global region.

Supplementary Figure S25: Posterior distributions of the estimated values of slopes in Model 1 using each of three individualism/collectivism scores as a dependent variable.

Supplementary Figure S26: Posterior distributions of the estimated values of slopes in Model 2 using each of three individualism/collectivism scores as a dependent variable.

Supplementary Figure S27: Posterior distributions of estimated values of slopes in Model 3 using each of three individualism/collectivism scores as a dependent variable.

Supplementary Tables

Supplementary Table S11: The number of countries or regions in each global region used for supplementary analysis.

Supplementary Table S12: Posterior distribution of zero-order correlation coefficients between each index of individualism/collectivism and other variables.

Supplementary Tables 13: WAIC values of each model using each of three individualism/collectivism scores as a dependent variable.

Supplementary Table S14: Numerical values of estimated parameter in Model 1 using both pathogen stress and government effectiveness as independent variables and each of three individualism/collectivism scores as a dependent variable.

Supplementary Table S15: Numerical values of estimated parameter in Model 2 using both pathogen stress and government effectiveness as independent variables and each of three individualism/collectivism scores as a dependent variable.

Supplementary Table 16: Numerical values of estimated parameter in Model 3 using both pathogen stress and government effectiveness as independent variables and each of three individualism/collectivism scores as a dependent variable.

Supplementary Tables S17: Summary of analysis results using both pathogen stress and government effectiveness as independent variables and each of three individualism/collectivism scores as a dependent variable.

Supplementary Table S18: Numerical values of estimated parameters in Model 1 using both pathogen stress and GDP per capita as an independent variable and each of three individualism/collectivism scores as a dependent variable.

Supplementary Table S19: Numerical values of estimated parameters in Model 2 using both pathogen stress and GDP per capita as an independent variable and each of three individualism/collectivism scores as a dependent variable.

Supplementary Table S20: Numerical values of estimated parameters in Model 3 using both pathogen stress and GDP per capita as an independent variable and each of three individualism/collectivism scores as a dependent variable.

Supplementary Tables S21: Summary of analysis results using both pathogen stress and GDP per capita as independent variables and each of three individualism/collectivism scores as a dependent variable.

Supplementary References

Supplementary Analysis

Other measures representing individualistic/collectivistic values

In this paper, we reported the results of an analysis using indices of individualism and collectivism as dependent variables in place of Hofstede's (2001) index of individualism. In addition to Hofstede's (2001) index, we used two representative indices of individualism and collectivism used in cross-cultural psychology, namely, the indices of Suh, Diner, Oishi, and Triandis (1998) and of Gelfand, Bhawuk, Nishii, and Bechtold (2004). Previous studies that have investigated the relationship between the historical prevalence of pathogens and the regional strength of collectivism (Fincher, Thornhill, Murray and Schaller, 2008; Thornhill, Fincher, Murray and Schaller, 2010; Murray and Schaller, 2010) have also used these indices. Although we have already reported the results using Hofstede's individualism score in our main text, we show them again here for comparison.

The index used by Suh et al. (1998) represents a regional level of individualism for each nation or region. As with Hofstede's index, higher scores mean greater individualism. Gelfand et al.'s (2004) index represents regional levels of collectivism. Higher scores mean greater collectivism. We used these scores for our analysis as was done in Fincher et al.'s (2008) coding.

Other studies (Fincher et al., 2008; Thornhill et al., 2010; Murray and Shaller, 2010) have reported results using an index of collectivism computed by Kashima and Kashima (1998). Kashima and Kashima (1998) argued that the pronoun-drop effect, a linguistic phenomenon, reflects collectivistic value, and they the reported presence or absence of the pronoun-drop effect for each country. Using this index, we tried to estimate parameter values under the assumption that a dependent variable obeys Bernoulli distribution because the variable was coded as a binary variable (presence of pronoun drop = 1, or absence = 0). However, we could not confirm the convergence of MCMC simulations, even when estimating parameter values in Model 2. For this reason, we reported results using three indices (Suh et al., 1998; Hofstede, 2001; Gelfand et al., 2004).

Summary of Supplementary Analysis

We did not find any major differences among the results using Hofstede's (2001) score as the dependent variable and those using other two indices. The summary of the analysis is as follows:

1. Whereas the individualism score of Hofstede (2001) was insignificantly correlated with the score of *Conformity 4*, both the individualism score of Suh et al. (1998) and the collectivism score of Gelfand et al. (2004) were significantly correlated with it. For other variables, similar tendencies for the correlation coefficients were found, regardless of the type of individualism and collectivism index (Supplementary Table S12).
2. When we used Suh et al.'s (1998) individualism score as a dependent variable and both pathogen stress and government effectiveness as independent variables, the WAIC value for Model 2 was the least of all three models. For its part, Model 3 was always the best model, regardless of the type of individualism and collectivism index (Supplementary Table S13).

3. When we used government effectiveness as an independent variable and Hofstede's (2001) index as a dependent variable, we found a significant global effect of government effectiveness in Model 3 (μ_{GE}). However, we could not find this significant effect when for Suh et al.'s (1998) score or Gelfand et al.'s (2004) (Supplementary Figure S27A, and Supplementary Table S16). For other parameters, similar results were found, regardless of the individualism and collectivism index used (See Supplementary Table S17).
4. When we used GDP per capita as an independent variable and Suh et al.'s (1998) score as a dependent variable, we could not find a significant global effect of pathogen stress in Model 2 (PS). Otherwise, results of global effects (i.e., significance of the parameter values of GDP, PS, μ_{GDP}, and μ_{GDP}) were similar, regardless of the type of either models or the individualism and collectivism index (Supplementary Table S21).

Thus, the results were almost consistent, regardless of the individualism and collectivism index.

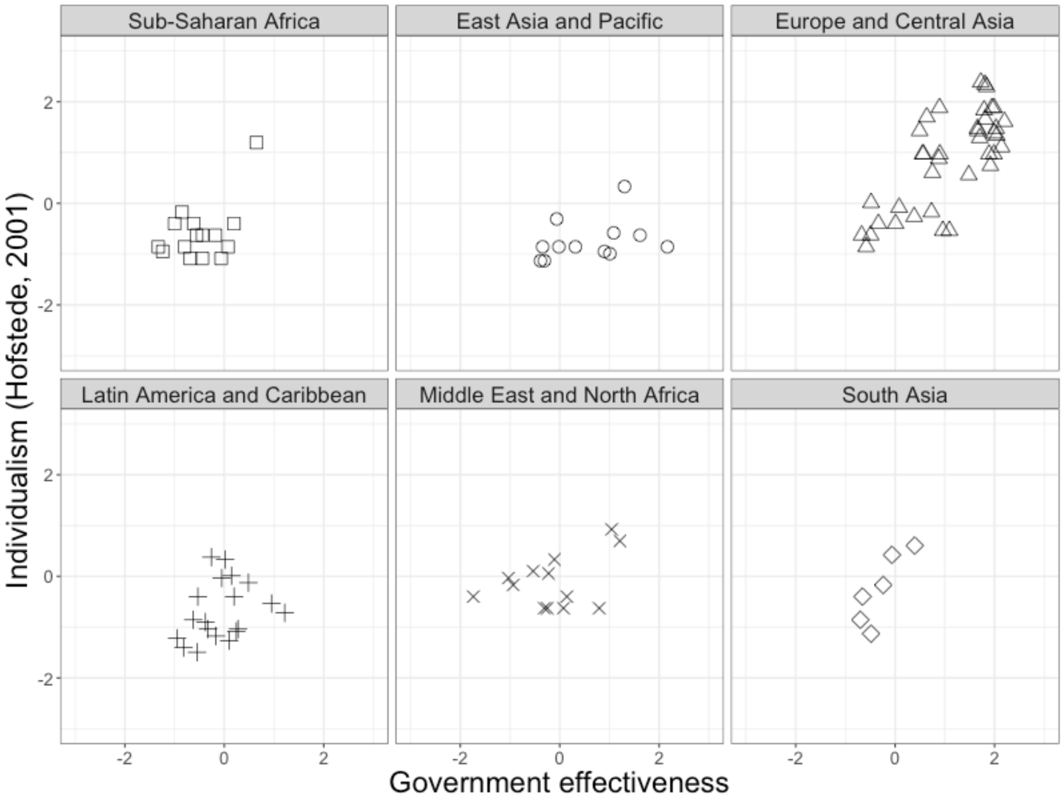

Supplementary Figure S16: Scatter plots displaying the correlation between regional level of government effectiveness and the indexes of *Individualism* (Hofstede, 2001) by global region. Each point represents a country or region.

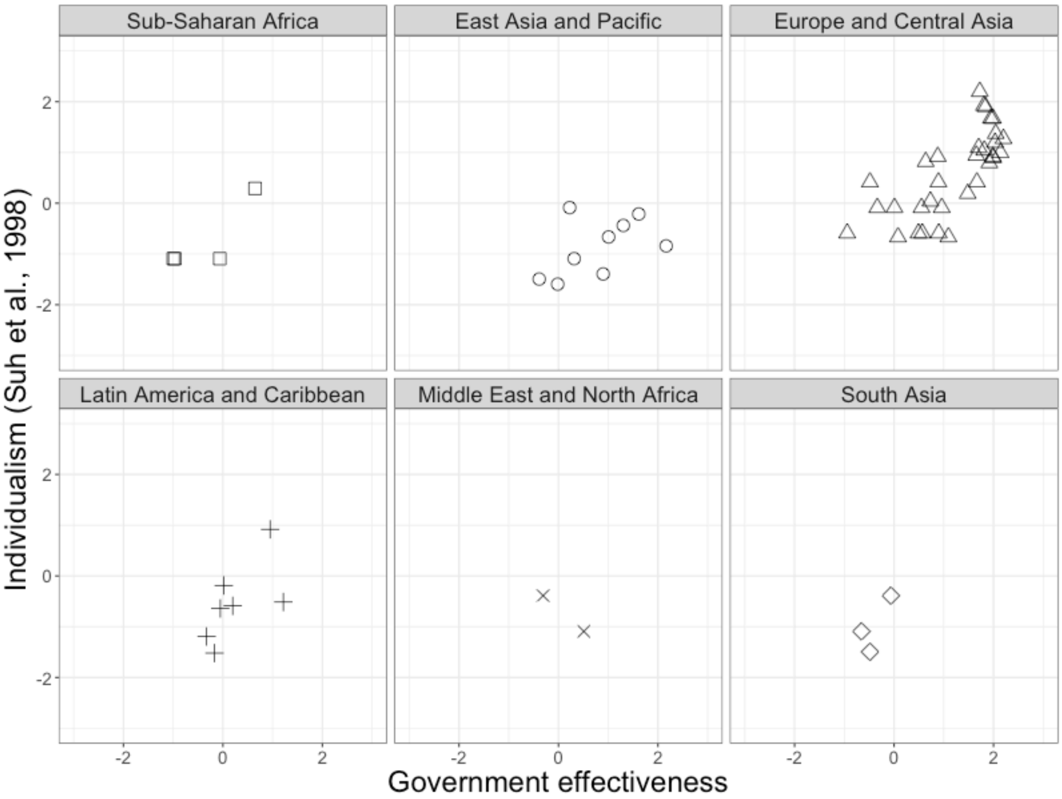

Supplementary Figure S17: Scatter plots displaying the correlation between regional level of government effectiveness and the indexes of *Individualism* (Suh et al., 1998) by global region. Each point represents a country or region.

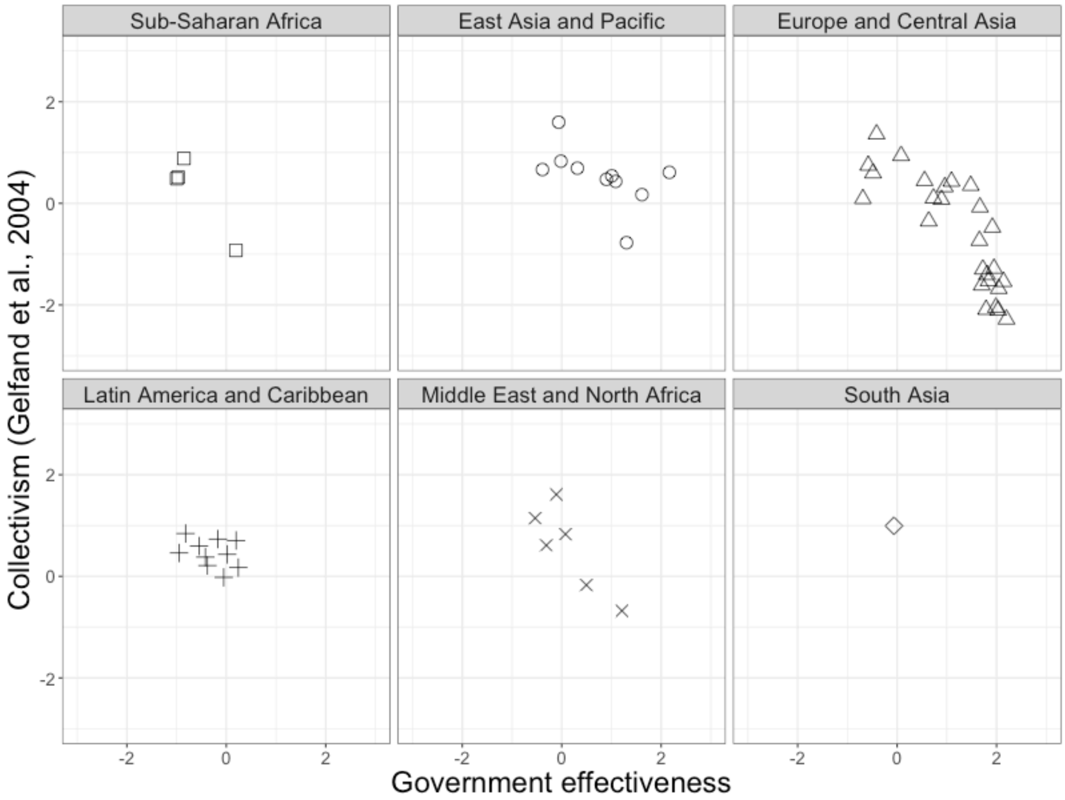

Supplementary Figure S18: Scatter plots displaying the correlation between regional level of government effectiveness and the indexes of *Collectivism* (Gelfand et al., 2004) by global region. Each point represents a country or region.

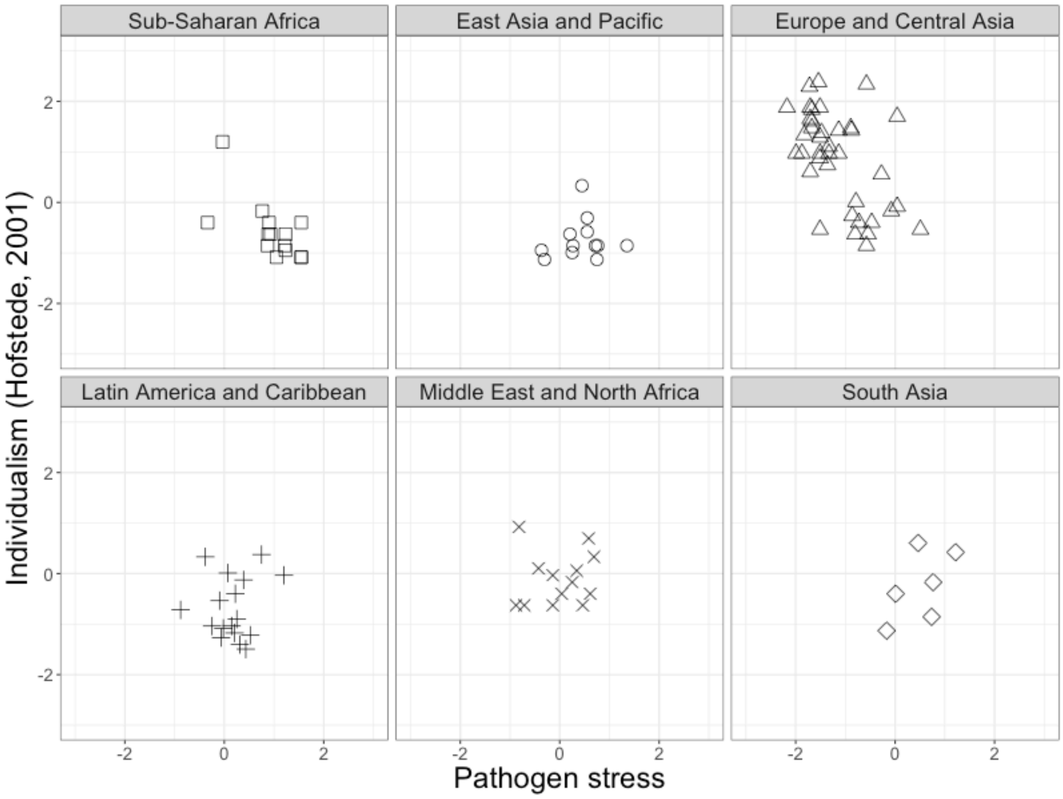

Supplementary Figure S19: Scatter plots displaying the correlation between regional level of pathogen stress and the index of *Individualism* (Hofstede, 2001) by each global region. Each point represents a country or region.

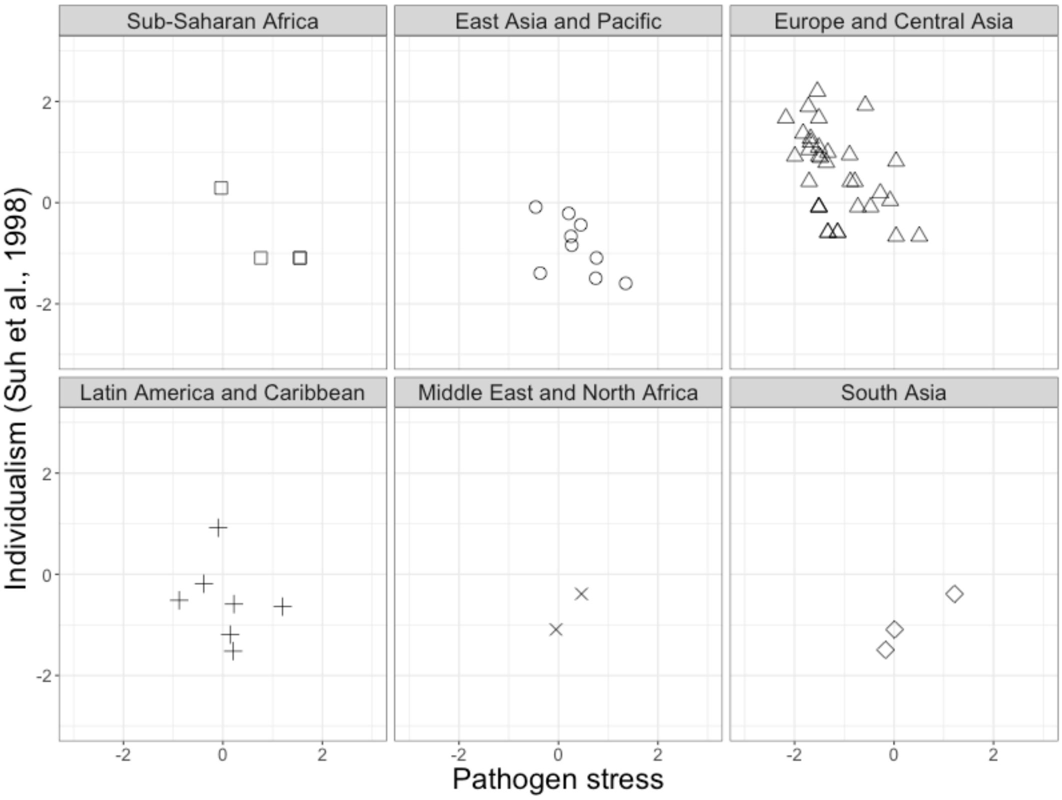

Supplementary Figure S20: Scatter plots displaying the correlation between regional level of pathogen stress and the index of *Individualism* (Suh et al., 1998) by each global region. Each point represents a country or region.

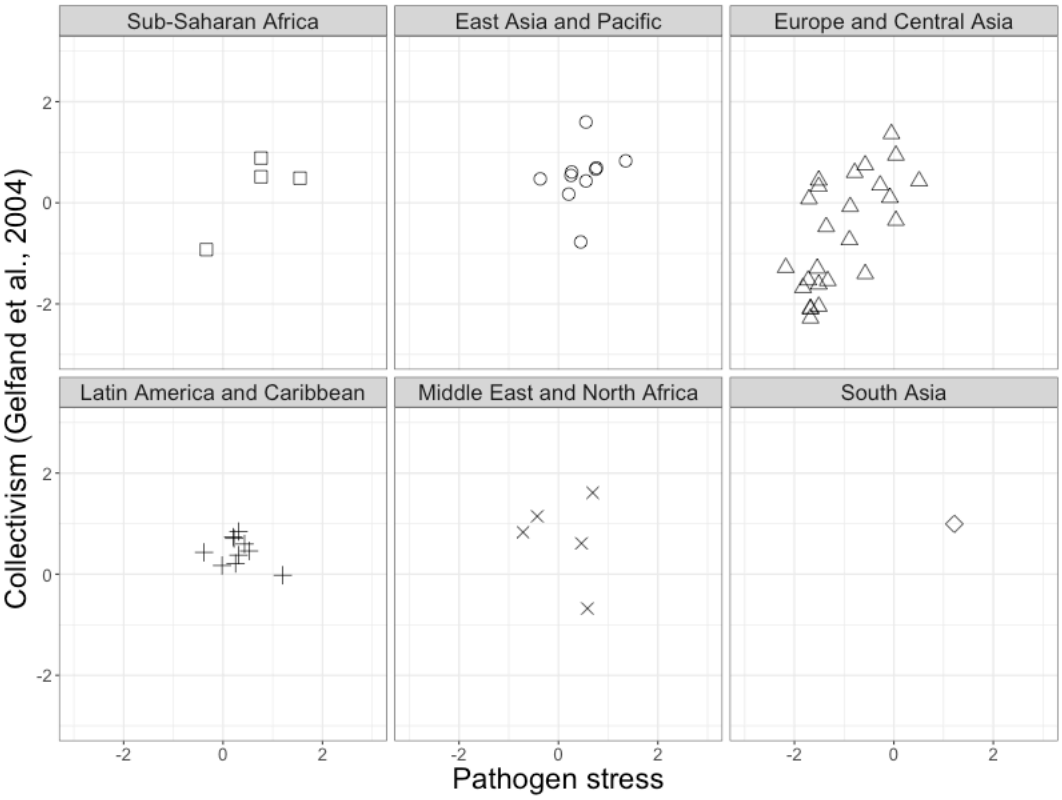

Supplementary Figure S21: Scatter plots displaying the correlation between regional level of pathogen stress and the index of *Collectivism* (Gelfand et al., 2004) by each global region. Each point represents a country or region.

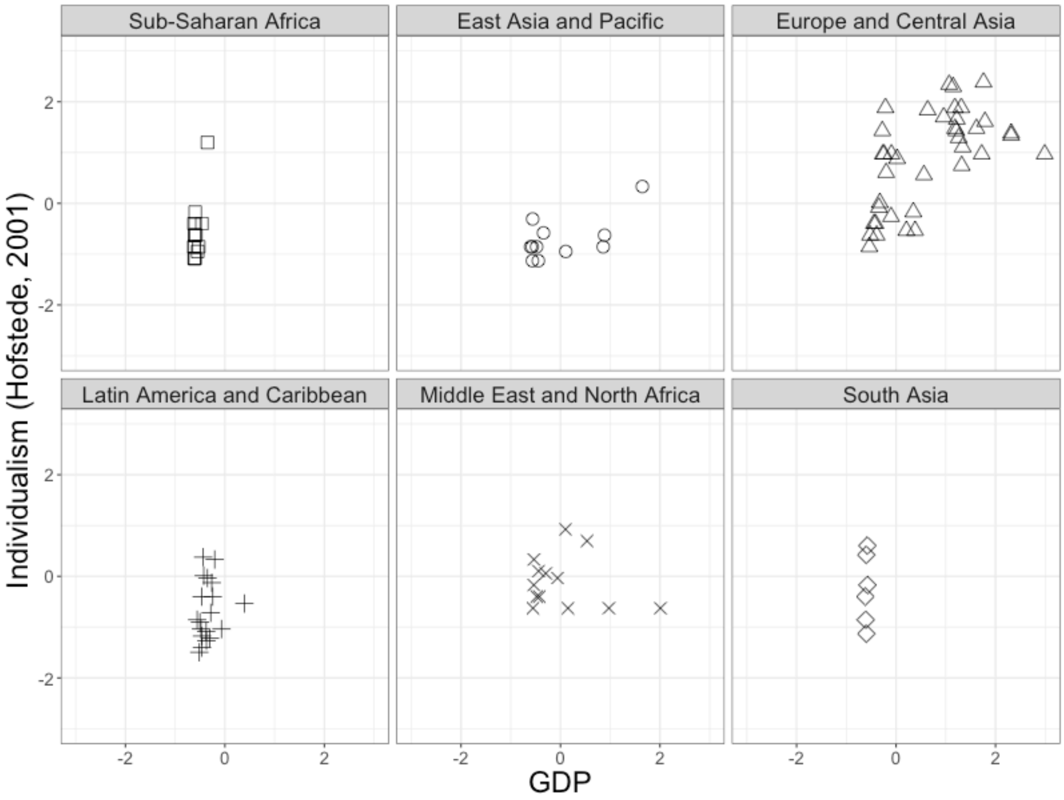

Supplementary Figure S22: Scatter plots displaying the correlation between regional level of GDP per capita and the index of *Individualism* (Hofstede, 2001) by global region. Each point represents a country or region.

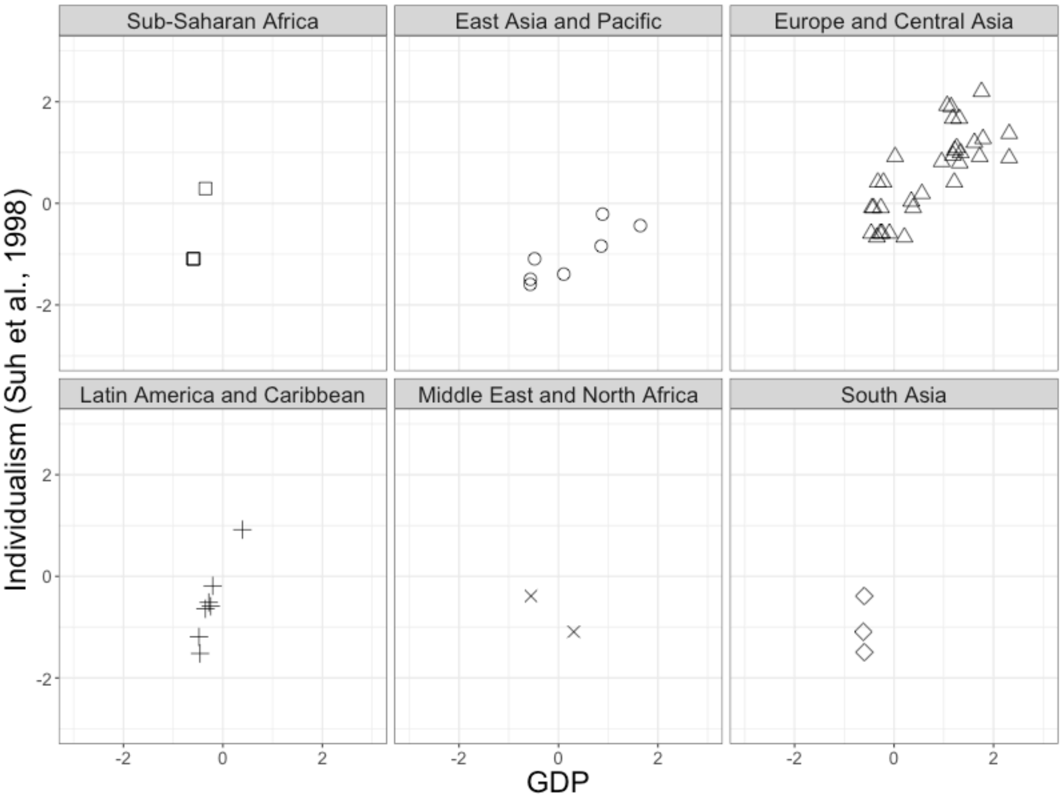

Supplementary Figure S23: Scatter plots displaying the correlation between regional level of GDP per capita and the index of *Individualism* (Suh et al., 1998) by global region. Each point represents a country or region.

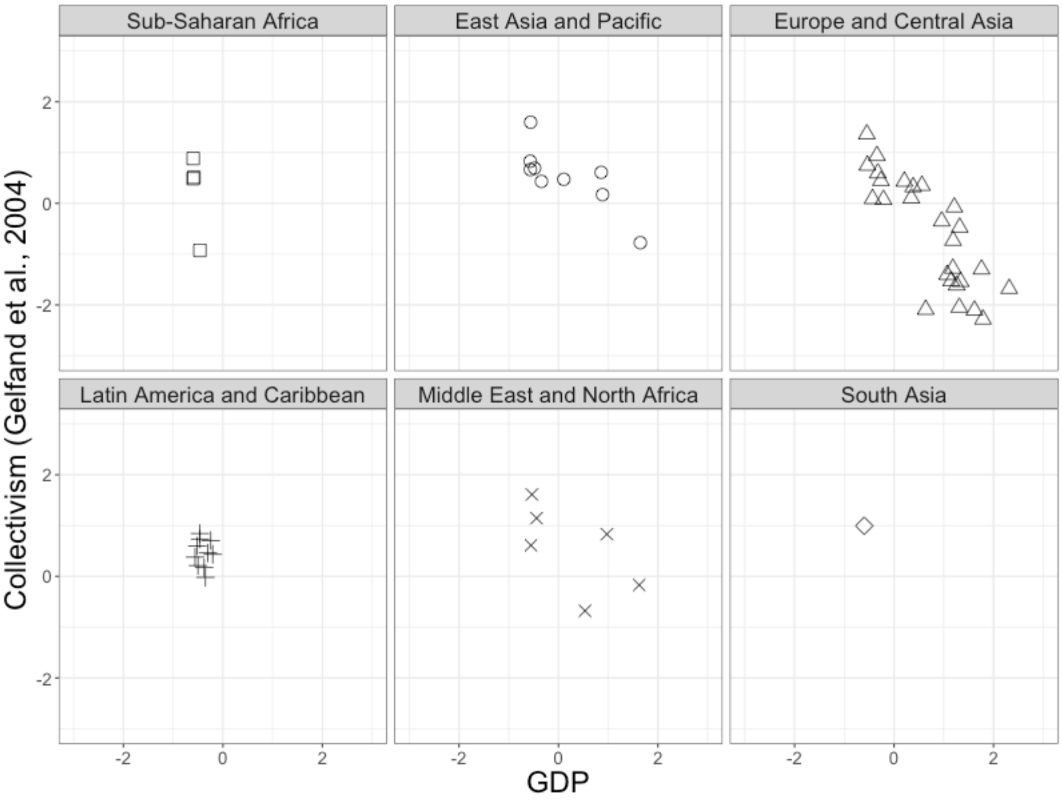

Supplementary Figure S24: Scatter plots displaying the correlation between regional level of GDP per capita and the index of *Collectivism* (Gelfand, 2004) by global region. Each point represents a country or region.

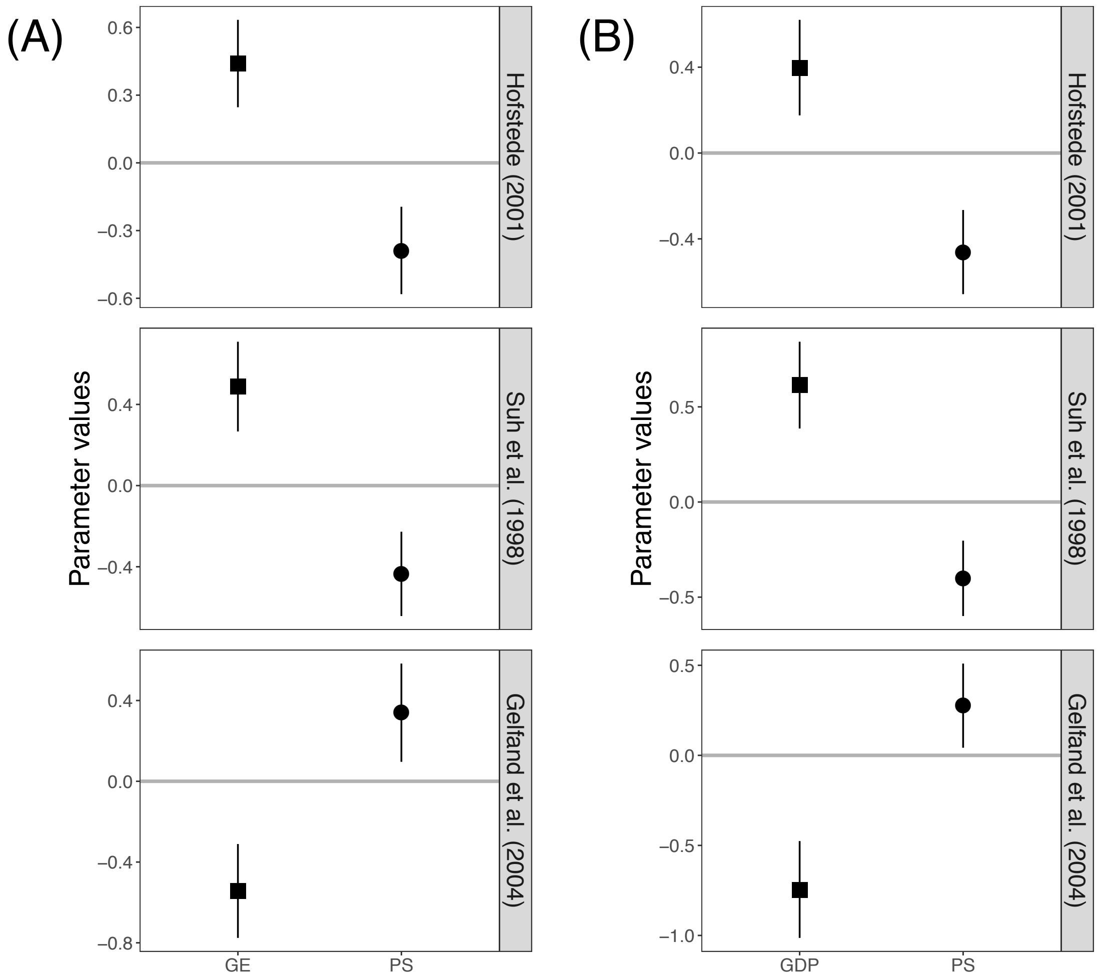

Figure S25: Posterior distributions of the estimated values of slopes in Model 1 using each of three individualism/collectivism scores as a dependent variable. (A) Results using government effectiveness as an independent variable. (B) Results using GDP as an independent variable instead of government effectiveness. Squares and circles represent posterior mean of slope affecting government effectiveness (or GDP per capita) and pathogen stress, respectively. Each error bar represents a 95% Bayesian credible interval.

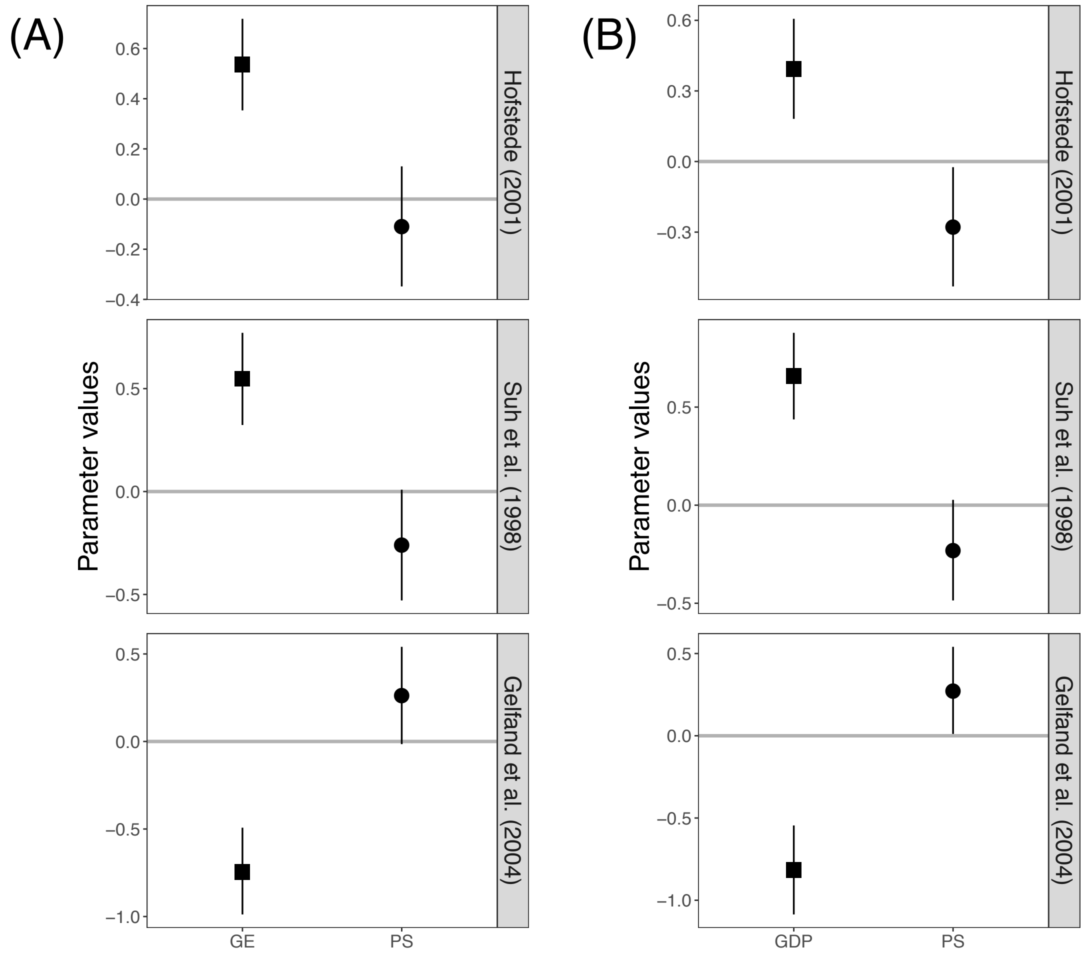

Supplementary Figure S26: Posterior distributions of the estimated values of slopes in Model 2 using each of three individualism/collectivism scores as a dependent variable. (A) Results using government effectiveness as an independent variable. (B) Results using GDP as an independent variable instead of government effectiveness. Squares and circles represent posterior means of slopes affecting government effectiveness (or GDP per capita) and pathogen stress, respectively. Each error bar represents a 95% Bayesian credible interval.

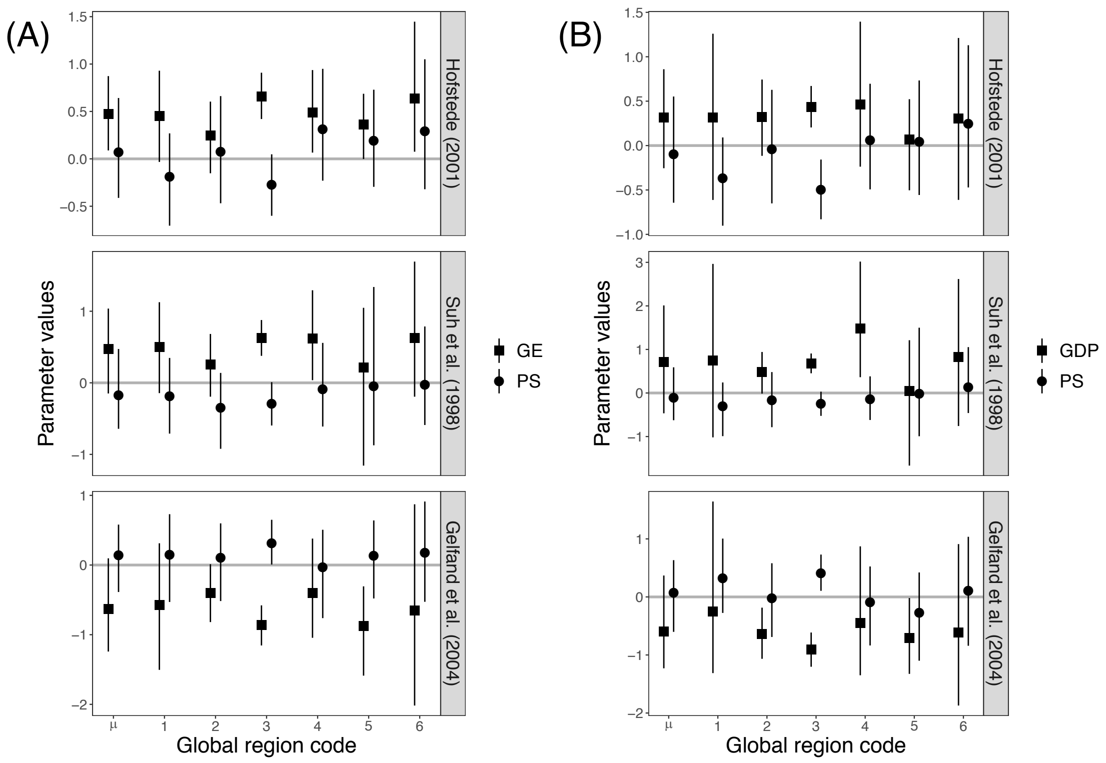

Supplementary Figure S27: Posterior distributions of estimated values of slopes in Model 3 using each of three individualism/collectivism scores as a dependent variable. (A) Results using government effectiveness as an independent variable. (B) Results using GDP as an independent variable instead of government effectiveness. Squares and circles represent posterior means of slopes affecting government effectiveness (or GDP per capita) and pathogen stress, respectively. Each number of horizontal axis means global regions (1 = Sub-Saharan Africa, 2 = East Asia and Pacific, 3 = Europe and Central Asia, 4 = Latin America and Caribbean, 5 = Middle East and North Africa, and 6 = South Asia). μ on the horizontal axis represents global slopes across global regions.

Supplementary Table S11: The number of countries or regions in each global region used for Supplementary Analysis (GE: government effectiveness, PS: pathogen stress).

	Dependent variables		
GE and PS were used as independent variables			
	Hofstede (2001)	Suh et al. (1998)	Gelfand et al. (2004)
Sub-Saharan Africa	14	4	4
East Asia and Pacific	12	9	10
Europe and Central Asia	37	32	25
Latin America and Caribbean	16	7	10
Middle East and North Africa	13	2	5
South Asia	6	3	1
Total	98	57	55
GDP per capita and PS were used for independent variables			
Sub-Saharan Africa	14	4	4
East Asia and Pacific	11	7	9
Europe and Central Asia	37	32	25
Latin America and Caribbean	16	7	10
Middle East and North Africa	13	2	5
South Asia	6	3	1
Total	97	55	54

Supplementary Table S12: Posterior distribution of zero-order correlation coefficients between each index of individualism/collectivism and other variables (PS: pathogen stress, GE: government effectiveness, GDP: GDP per capita). *ESS* represents effective sample sizes of MCMC simulations.

			Quantiles			Sample	
	Mean	*SD*	2.5%	50%	97.5%	Size	*ESS*
Correlation coefficients between *individualism* (Hofstede, 2001) with							
PS	–0.66	0.06	–0.76	–0.67	–0.54	100	11656
GE	0.68	0.05	0.57	0.69	0.78	103	10671
GDP	0.63	0.06	0.50	0.63	0.74	102	12100
Conformity 1	–0.63	0.07	–0.76	–0.64	–0.47	68	11501
Conformity 2	–0.40	0.10	–0.59	–0.41	–0.18	66	16000
Conformity 3	–0.40	0.10	–0.57	–0.40	–0.20	78	16000
Conformity 4	0.26	0.14	–0.04	0.26	0.51	44	16000
Correlation coefficients between *individualism* (Suh et al., 1998) with							
PS	–0.69	0.07	–0.81	–0.69	–0.53	58	10527
GE	0.71	0.07	0.56	0.71	0.82	57	8964
GDP	0.75	0.06	0.61	0.75	0.85	55	9495
Conformity 1	–0.47	0.12	–0.67	–0.48	–0.22	47	16000
Conformity 2	–0.49	0.11	–0.68	–0.50	–0.24	47	14562
Conformity 3	–0.31	0.12	–0.53	–0.31	–0.05	55	16000
Conformity 4	0.33	0.16	0.008	0.34	0.60	34	16000
Correlation coefficients between *collectivism* (Gelfand et al., 2004) with							
PS	0.67	0.08	0.50	0.67	0.80	55	10707
GE	–0.73	0.06	–0.84	–0.74	–0.59	57	9240
GDP	–0.77	0.06	–0.86	–0.77	–0.64	56	9816
Conformity 1	0.74	0.07	0.58	0.75	0.86	44	9524
Conformity 2	0.65	0.09	0.44	0.66	0.80	43	11487
Conformity 3	0.45	0.12	0.20	0.45	0.65	50	16000
Conformity 4	–0.46	0.15	–0.71	–0.47	–0.13	31	12865

Supplementary Tables S13: WAIC values of each model using each of three individualism/collectivism scores as a dependent variable.

Results in which government effectiveness was used as an independent variable

	Individualism (Hofstede, 2001)	*Individualism* (Suh et al., 1998)	*Collectivism* (Gelfand et al., 2004)
Model 1	212.53	111.71	110.22
Model 2	189.86	107.01	100.21
Model 3	181.86	107.51	96.86

Results in which GDP per capita was used as an independent variable

	Individualism (Hofstede, 2001)	*Individualism* (Suh et al., 1998)	*Collectivism* (Gelfand et al., 2004)
Model 1	218.11	101.85	102.57
Model 2	208.89	96.97	101.07
Model 3	202.40	94.02	97.50

Supplementary Table S14: Numerical values of estimated parameter in Model 1 using both pathogen stress and government effectiveness as independent variables and each of three individualism/collectivism scores as a dependent variable. GE and PS represent government effectiveness and pathogen stress, respectively. *ESS* represents effective sample sizes of MCMC simulations. Rows shaded in gray indicate the effects of slopes in which 95% Bayesian credible intervals of values did not include zero.

			Quantiles			
	Mean	*SD*	2.5%	50%	97.5%	*ESS*
Individualism (Hofstede, 2001)						
a_0	–0.22	0.07	–0.37	–0.22	–0.07	12987
GE	0.43	0.10	0.24	0.43	0.63	10274
PS	–0.39	0.10	–0.59	–0.39	–0.20	10553
σ_y	0.69	0.05	0.60	0.68	0.80	14460
Individualism (Suh et al., 1998)						
a_0	–0.59	0.11	–0.81	–0.59	–0.38	11334
GE	0.49	0.11	0.27	0.49	0.71	7817
PS	–0.44	0.11	–0.64	–0.44	–0.23	9003
σ_y	0.63	0.06	0.53	0.63	0.77	11947
Collectivism (Gelfand et al., 2004)						
a_0	0.43	0.1	0.22	0.43	0.64	11267
GE	–0.54	0.12	–0.78	–0.54	–0.31	8797
PS	0.34	0.12	0.10	0.34	0.58	10025
σ_y	0.65	0.07	0.53	0.64	0.79	13362

Supplementary Table S15: Numerical values of estimated parameter in Model 2 using both pathogen stress and government effectiveness as independent variables and each of three individualism/collectivism scores as a dependent variable. GE and PS represent government effectiveness and pathogen stress, respectively. Subscript numbers under *a* represent global regions (1: Sub-Saharan Africa, 2 East Asia and Pacific, 3: Europe and Central Asia, 4: Latin America and Caribbean, 5: Middle East and North Africa, and 6: South Asia). *ESS* represents effective sample sizes of MCMC simulations. Rows shaded in gray indicate the effects of slopes in which 95% Bayesian credible intervals of values did not include zero.

			Quantiles			
	Mean	*SD*	2.5%	50%	97.5%	*ESS*
Individualism (Hofstede, 2001)						
μ_a	–0.29	0.30	–0.90	–0.29	0.30	6659
GE	0.52	0.10	0.33	0.52	0.70	13209
PS	–0.12	0.12	–0.36	–0.12	0.12	10080
a_1	–0.20	0.18	–0.56	–0.20	0.14	16000
a_2	–0.92	0.20	–1.33	–0.92	–0.52	11487
a_3	0.15	0.16	–0.16	0.15	0.46	13463
a_4	–0.63	0.15	–0.92	–0.62	–0.33	16000
a_5	–0.07	0.16	–0.39	–0.07	0.24	16000
a_6	–0.11	0.23	–0.56	–0.11	0.35	16000
σ_a	0.61	0.36	0.24	0.52	1.50	5926
σ_y	0.60	0.05	0.52	0.60	0.70	16000
Individualism (Suh et al., 1998)						
μ_a	–0.65	0.24	–1.15	–0.65	–0.18	9374
GE	0.55	0.11	0.32	0.55	0.77	9101
PS	–0.26	0.14	–0.53	–0.26	0.01	6821
a_1	–0.45	0.25	–0.93	–0.46	0.07	16000
a_2	–1.07	0.24	–1.54	–1.07	–0.59	5885
a_3	–0.38	0.18	–0.72	–0.38	–0.02	5168
a_4	–0.66	0.20	–1.05	–0.66	–0.27	16000
a_5	–0.69	0.30	–1.32	–0.69	–0.11	16000
a_6	–0.66	0.27	–1.21	–0.66	–0.13	16000
σ_a	0.45	0.28	0.11	0.39	1.17	5109
σ_y	0.59	0.06	0.48	0.58	0.72	16000

Supplementary Table S15 (cond.): Numerical values of estimated parameters in Model 2 using both pathogen stress and government effectiveness as independent variables and each of three individualism/collectivism scores as a dependent variable. GE and PS represent government effectiveness and pathogen stress, respectively. Subscript numbers under *a* represent global regions (1: Sub-Saharan Africa, 2 East Asia and Pacific, 3: Europe and Central Asia, 4: Latin America and Caribbean, 5: Middle East and North Africa, and 6: South Asia). *ESS* represents effective sample sizes of MCMC simulations. Rows shaded in gray indicate the effects of slopes in which 95% Bayesian credible intervals of values did not include zero.

			Quantiles			
	Mean	*SD*	2.5%	50%	97.5%	*ESS*
Collectivism (Gelfand et al., 2004)						
μ_a	0.45	0.33	–0.21	0.45	1.12	9246
GE	–0.74	0.13	–0.99	–0.75	–0.49	9531
PS	0.26	0.14	–0.02	0.26	0.54	11357
a_1	–0.22	0.32	–0.84	–0.22	0.40	9579
a_2	0.92	0.23	0.47	0.92	1.38	8788
a_3	0.60	0.18	0.24	0.60	0.97	13149
a_4	0.19	0.18	–0.16	0.19	0.55	16000
a_5	0.66	0.24	0.21	0.66	1.13	16000
a_6	0.54	0.43	–0.31	0.53	1.42	16000
σ_a	0.65	0.39	0.19	0.56	1.62	6372
σ_y	0.57	0.06	0.47	0.56	0.70	16000

Supplementary Table S16: Numerical values of estimated parameters in Model 3 using both pathogen stress and government effectiveness as independent variables and each of three individualism/collectivism scores as a dependent variable. GE and PS represent government effectiveness and pathogen stress, respectively. Subscript numbers under *a*, GE, and PS represent global regions (1: Sub-Saharan Africa, 2 East Asia and Pacific, 3: Europe and Central Asia, 4: Latin America and Caribbean, 5: Middle East and North Africa, and 6: South Asia). *ESS* represents effective sample sizes of MCMC simulations. Rows shaded in gray indicate the effects of slopes in which 95% Bayesian credible intervals of values did not include zero.

			Quantiles			
	Mean	*SD*	2.5%	50%	97.5%	*ESS*
Individualism (Hofstede, 2001)						
μ_a	–0.38	0.26	–0.91	–0.37	0.15	3862
μ_{GE}	0.48	0.21	0.09	0.47	0.91	6506
μ_{PS}	0.07	0.27	–0.42	0.05	0.64	6491
a_1	–0.19	0.24	–0.64	–0.20	0.31	8929
a_2	–0.84	0.25	–1.32	–0.83	–0.37	5649
a_3	–0.16	0.18	–0.52	–0.16	0.20	7139
a_4	–0.69	0.15	–0.99	–0.69	–0.40	16000
a_5	–0.11	0.15	–0.41	–0.11	0.20	16000
a_6	–0.26	0.27	–0.79	–0.27	0.27	8660
GE_1	0.45	0.24	–0.05	0.45	0.93	9809
GE_2	0.24	0.19	–0.16	0.25	0.58	5604
GE_3	0.64	0.13	0.39	0.64	0.90	3670
GE_4	0.52	0.24	0.07	0.51	1.02	10540
GE_5	0.36	0.17	–0.001	0.36	0.68	8706
GE_6	0.64	0.34	0.07	0.59	1.45	7417
PS_1	–0.19	0.25	–0.70	–0.17	0.28	8103
PS_2	0.07	0.28	–0.47	0.06	0.66	8904
PS_3	–0.28	0.16	–0.60	–0.28	0.04	7035
PS_4	0.32	0.31	–0.23	0.30	0.95	6186
PS_5	0.19	0.26	–0.29	0.18	0.72	9044
PS_6	0.29	0.35	–0.32	0.25	1.05	7073
σ_a	0.51	0.32	0.15	0.43	1.28	5637
σ_{GE}	0.34	0.26	0.05	0.28	0.97	3790
σ_{PS}	0.47	0.34	0.09	0.39	1.29	3960
σ_y	0.56	0.04	0.48	0.56	0.66	16000

Supplementary Table S16 (cond.): Numerical values of estimated parameters in Model 3 using both pathogen stress and government effectiveness as independent variables and each of three individualism/collectivism scores as a dependent variable. GE and PS represent government effectiveness and pathogen stress, respectively. Subscript numbers under *a*, GE, and PS represent global regions (1: Sub-Saharan Africa, 2 East Asia and Pacific, 3: Europe and Central Asia, 4: Latin America and Caribbean, 5: Middle East and North Africa, and 6: South Asia). *ESS* represents effective sample sizes of MCMC simulations. Rows shaded in gray indicate the effects of slopes in which 95% Bayesian credible intervals of values did not include zero.

			Quantiles			
	Mean	*SD*	2.5%	50%	97.5%	*ESS*
Individualism (Suh et al., 1998)						
μ_a	–0.65	0.20	–1.04	–0.65	–0.27	4927
μ_{GE}	0.47	0.29	–0.15	0.48	1.04	4734
μ_{PS}	–0.17	0.29	–0.64	–0.19	0.47	1109
a_1	–0.53	0.26	–1.00	–0.55	0.05	3374
a_2	–0.83	0.25	–1.37	–0.80	–0.41	2869
a_3	–0.51	0.18	–0.85	–0.52	–0.14	2978
a_4	–0.67	0.19	–1.04	–0.67	–0.29	3751
a_5	–0.68	0.26	–1.24	–0.67	–0.18	6903
a_6	–0.68	0.26	–1.23	–0.67	–0.17	4748
GE_1	0.50	0.31	–0.14	0.50	1.13	6673
GE_2	0.26	0.23	–0.19	0.26	0.68	2335
GE_3	0.62	0.13	0.38	0.62	0.88	3386
GE_4	0.62	0.31	0.04	0.60	1.29	5616
GE_5	0.21	0.55	–1.16	0.33	1.05	2762
GE_6	0.62	0.45	–0.19	0.58	1.69	5779
PS_1	–0.19	0.26	–0.71	–0.20	0.35	5397
PS_2	–0.35	0.26	–0.92	–0.33	0.14	1530
PS_3	–0.30	0.15	–0.60	–0.29	0.01	3024
PS_4	–0.09	0.30	–0.61	–0.12	0.56	1591
PS_5	–0.05	0.53	–0.87	–0.14	1.34	563
PS_6	–0.03	0.35	–0.59	–0.08	0.79	1679
σ_a	0.29	0.25	0.03	0.24	0.91	2314
σ_{GE}	0.46	0.39	0.05	0.35	1.50	2013
σ_{PS}	0.37	0.38	0.04	0.27	1.24	816
σ_y	0.58	0.06	0.47	0.57	0.71	7244

Supplementary Table S16 (cond.): Numerical values of estimated parameter in Model 3 using both pathogen stress and government effectiveness as independent variables and each of three individualism/collectivism scores as a dependent variable. GE and PS represent government effectiveness and pathogen stress, respectively. Subscript numbers under *a*, GE, and PS represent global regions (1: Sub-Saharan Africa, 2 East Asia and Pacific, 3: Europe and Central Asia, 4: Latin America and Caribbean, 5: Middle East and North Africa, and 6: South Asia). *ESS* represents effective sample sizes of MCMC simulations. Rows shaded in gray indicate the effects of slopes in which 95% Bayesian credible intervals of values did not include zero.

			Quantiles			
	Mean	*SD*	2.5%	50%	97.5%	*ESS*
Collectivism (Gelfand et al., 2004)						
μ_a	0.54	0.30	–0.07	0.53	1.15	3108
μ_{GE}	–0.63	0.35	–1.24	–0.66	0.10	2515
μ_{PS}	0.14	0.24	–0.39	0.16	0.58	3037
a_1	–0.004	0.41	–0.82	0.01	0.68	180
a_2	0.75	0.28	0.27	0.73	1.35	481
a_3	0.78	0.20	0.42	0.78	1.18	190
a_4	0.39	0.20	–0.02	0.39	0.75	1333
a_5	0.68	0.21	0.29	0.67	1.12	486
a_6	0.63	0.45	–0.25	0.59	1.60	6398
GE_1	–0.57	0.46	–1.50	–0.60	0.31	112
GE_2	–0.40	0.21	–0.82	–0.39	0.01	3801
GE_3	–0.86	0.15	–1.15	–0.86	–0.58	402
GE_4	–0.40	0.36	–1.04	–0.41	0.38	2354
GE_5	–0.88	0.32	–1.59	–0.84	–0.31	2335
GE_6	–0.65	0.72	–2.02	–0.67	0.87	522
PS_1	0.15	0.31	–0.53	0.17	0.73	258
PS_2	0.10	0.28	–0.52	0.13	0.60	697
PS_3	0.31	0.16	0.01	0.30	0.65	4911
PS_4	–0.03	0.32	–0.76	0.01	0.51	2833
PS_5	0.13	0.28	–0.48	0.16	0.64	241
PS_6	0.18	0.34	–0.53	0.17	0.91	3500
σ_a	0.53	0.40	0.03	0.45	1.49	199
σ_{GE}	0.59	0.51	0.09	0.44	1.75	86
σ_{PS}	0.34	0.29	0.04	0.27	1.09	1592
σ_y	0.54	0.06	0.43	0.53	0.67	272

Tables S17: Summary of analysis results using both pathogen stress and government effectiveness as independent variables and each of three individualism/collectivism scores as a dependent variable. Parameters in parentheses indicate the parameters in Model 3. Asterisks in columns of global effects (GE, PS, μ_{GE}, or μ_{PS}) indicate that the effect was significant (i.e., the 95% Bayesian credible interval of the effect did not include zero). Numbers in columns of region–specific effects (GE_j or PS_j) indicate global regions in which significant effects were found (1 = Sub-Saharan Africa, 2 = East Asia and Pacific, 3 = Europe and Central Asia, 4 = Latin America and Caribbean, 5 = Middle East and North Africa, and 6 = South Asia). Rows shaded in gray indicate the best model, in which the WAIC value was smallest in three models.

Dependent variables	Model	Parameters			
		GE (μ_{GE})	PS (μ_{PS})	(GE_j)	(PS_j)
Individualism	Model 1	*	*		
(Hofstede, 2001)	Model 2	*			
	Model 3	*		3,4,6	
Individualism	Model 1	*	*		
(Suh et al., 1998)	Model 2	*			
	Model 3			3, 4	
Collectivism	Model 1	*	*		
(Gelfand et al., 2004)	Model 2	*			
	Model 3			3, 5	3

Supplementary Table S18: Numerical values of estimated parameters in Model 1 using both pathogen stress and GDP per capita as an independent variable and each of three individualism/collectivism scores as a dependent variable. GDP and PS represent GDP per capita and pathogen stress, respectively. *ESS* represents effective sample sizes of MCMC simulations. Rows shaded in gray indicate the effects of slopes in which 95% Bayesian credible intervals of values did not include zero.

			Quantiles			
	Mean	*SD*	2.5%	50%	97.5%	*ESS*
Individualism (Hofstede, 2001)						
a_0	–0.09	0.07	–0.24	–0.09	0.05	16000
GDP	0.38	0.12	0.16	0.38	0.61	12243
PS	–0.47	0.10	–0.66	–0.47	–0.27	12264
σ_y	0.71	0.05	0.62	0.71	0.83	14848
Individualism (Suh et al., 1998)						
a_0	–0.40	0.09	–0.58	–0.40	–0.22	13993
GDP	0.62	0.12	0.39	0.62	0.84	11583
PS	–0.40	0.10	–0.60	–0.40	–0.20	11148
σ_y	0.60	0.06	0.49	0.59	0.73	14015
Collectivism (Gelfand et al., 2004)						
a_0	0.25	0.09	0.08	0.25	0.42	14188
GDP	–0.75	0.14	–1.01	–0.75	–0.48	10886
PS	0.28	0.12	0.04	0.28	0.51	10875
σ_y	0.61	0.06	0.50	0.61	0.75	13595

Supplementary Table S19: Numerical values of estimated parameters in Model 2 using both pathogen stress and GDP per capita as an independent variable and each of three individualism/collectivism scores as a dependent variable. GDP and PS represent GDP per capita and pathogen stress, respectively. Subscript numbers under *a* represent global regions (1: Sub-Saharan Africa, 2 East Asia and Pacific, 3: Europe and Central Asia, 4: Latin America and Caribbean, 5: Middle East and North Africa, and 6: South Asia). *ESS* represents effective sample sizes of MCMC simulations. Rows shaded in gray indicate the effects of slopes in which 95% Bayesian credible intervals of values did not include zero.

			Quantiles			
	Mean	*SD*	2.5%	50%	97.5%	*ESS*
Individualism (Hofstede, 2001)						
μ_a	–0.15	0.23	–0.63	–0.15	0.31	9107
GDP	0.37	0.11	0.15	0.37	0.58	14978
PS	–0.29	0.13	–0.54	–0.29	–0.03	7169
a_1	–0.11	0.18	–0.48	–0.11	0.24	16000
a_2	–0.49	0.21	–0.90	–0.48	–0.10	8891
a_3	0.28	0.17	–0.06	0.28	0.62	7348
a_4	–0.45	0.17	–0.79	–0.45	–0.13	9746
a_5	–0.14	0.17	–0.47	–0.13	0.19	16000
a_6	0.01	0.24	–0.44	0.01	0.50	16000
σ_a	0.47	0.28	0.14	0.40	1.19	6419
σ_y	0.66	0.05	0.58	0.66	0.77	10714
Individualism (Suh et al., 1998)						
μ_a	–0.46	0.24	–0.96	–0.46	0.01	10591
GDP	0.66	0.11	0.44	0.66	0.88	16000
PS	–0.23	0.13	–0.49	–0.23	0.03	10005
a_1	–0.28	0.25	–0.75	–0.28	0.22	16000
a_2	–0.92	0.24	–1.38	–0.92	–0.47	8192
a_3	–0.17	0.16	–0.48	–0.17	0.15	10532
a_4	–0.39	0.18	–0.75	–0.39	–0.02	16000
a_5	–0.54	0.30	–1.15	–0.53	0.03	16000
a_6	–0.49	0.26	–1.02	–0.49	0.01	16000
σ_a	0.46	0.28	0.13	0.40	1.19	6401
σ_y	0.55	0.06	0.45	0.55	0.68	16000

Supplementary Table S19 (cond.): Numerical values of estimated parameters in Model 2 using both pathogen stress and GDP per capita as an independent variable and each of three individualism/collectivism scores as a dependent variable. GDP and PS represent GDP per capita and pathogen stress, respectively. Subscript numbers under *a* represent global regions (1: Sub-Saharan Africa, 2 East Asia and Pacific, 3: Europe and Central Asia, 4: Latin America and Caribbean, 5: Middle East and North Africa, 6: South Asia). *ESS* represents effective sample sizes of MCMC simulations. Rows shaded in gray indicate the effects of slopes in which 95% Bayesian credible intervals of values did not include zero.

			Quantiles			
	Mean	*SD*	2.5%	50%	97.5%	*ESS*
Collectivism (Gelfand et al., 2004)						
μ_a	0.23	0.24	–0.26	0.24	0.67	6679
GDP	–0.82	0.14	–1.09	–0.82	–0.55	5520
PS	0.27	0.13	0.01	0.27	0.54	7167
a_1	–0.12	0.30	–0.74	–0.10	0.37	2097
a_2	0.40	0.19	0.06	0.39	0.78	7312
a_3	0.31	0.16	0.01	0.30	0.63	10905
a_4	0.10	0.18	–0.26	0.11	0.43	5507
a_5	0.49	0.24	0.07	0.47	0.98	3577
a_6	0.21	0.35	–0.54	0.22	0.91	11252
σ_a	0.41	0.32	0.06	0.35	1.18	2432
σ_y	0.58	0.06	0.48	0.58	0.72	3910

Supplementary Table S20: Numerical values of estimated parameters in Model 3 using both pathogen stress and GDP per capita as an independent variable and each of three individualism/collectivism scores as a dependent variable. GDP and PS represent GDP per capita and pathogen stress, respectively. Subscript numbers under *a*, GDP, and PS represent global regions (1: Sub-Saharan Africa, 2 East Asia and Pacific, 3: Europe and Central Asia, 4: Latin America and Caribbean, 5: Middle East and North Africa, and 6: South Asia). *ESS* represents effective sample sizes of MCMC simulations. Rows shaded in gray indicate the effects of slopes when 95% Bayesian credible intervals of values did not include zero.

			Quantiles			
	Mean	*SD*	2.5%	50%	97.5%	*ESS*
Individualism (Hofstede, 2001)						
μ_a	–0.25	0.24	–0.73	–0.25	0.24	3584
μ_{GDP}	0.33	0.33	–0.28	0.32	1.03	4622
μ_{PS}	–0.09	0.30	–0.62	–0.11	0.57	4557
a_1	–0.06	0.32	–0.62	–0.09	0.64	3938
a_2	–0.59	0.23	–1.07	–0.58	–0.15	6102
a_3	0.02	0.21	–0.39	0.01	0.42	1192
a_4	–0.46	0.23	–0.90	–0.47	0.02	5781
a_5	–0.15	0.16	–0.46	–0.15	0.17	8446
a_6	–0.22	0.34	–0.86	–0.22	0.50	4072
GDP_1	0.33	0.51	–0.64	0.32	1.45	6547
GDP_2	0.32	0.21	–0.10	0.32	0.75	16000
GDP_3	0.41	0.12	0.17	0.41	0.65	3669
GDP_4	0.57	0.55	–0.28	0.47	1.96	4800
GDP_5	0.04	0.27	–0.53	0.06	0.52	2679
GDP_6	0.31	0.50	–0.68	0.31	1.40	7401
PS_1	–0.38	0.25	–0.90	–0.37	0.10	6394
PS_2	–0.03	0.33	–0.64	–0.06	0.66	2641
PS_3	–0.50	0.17	–0.83	–0.50	–0.16	2024
PS_4	0.07	0.31	–0.49	0.06	0.72	2595
PS_5	0.03	0.33	–0.58	0.02	0.71	4825
PS_6	0.24	0.42	–0.47	0.21	1.14	2729
σ_a	0.44	0.29	0.09	0.38	1.16	3917
σ_{GDP}	0.47	0.44	0.05	0.35	1.62	2014
σ_{PS}	0.54	0.41	0.09	0.45	1.56	413
σ_y	0.63	0.05	0.54	0.62	0.73	5305

Supplementary Table S20 (cond.): Numerical values of estimated parameters in Model 3 using both pathogen stress and GDP per capita as an independent variable and each of three individualism/collectivism scores as a dependent variable. GDP and PS represent GDP per capita and pathogen stress, respectively. Subscript numbers under *a*, GDP, and PS represent global regions (1: Sub-Saharan Africa, 2 East Asia and Pacific, 3: Europe and Central Asia, 4: Latin America and Caribbean, 5: Middle East and North Africa, 6: South Asia). *ESS* represents effective sample sizes of MCMC simulations. Rows shaded in gray indicate the effects of slopes when 95% Bayesian credible intervals of values did not include zero.

			Quantiles			
	Mean	*SD*	2.5%	50%	97.5%	*ESS*
Individualism (Suh et al., 1998)						
μ_a	–0.45	0.33	–1.10	–0.46	0.22	4780
μ_{GDP}	0.71	0.61	–0.47	0.68	2.01	3070
μ_{PS}	–0.11	0.35	–0.63	–0.15	0.59	1492
a_1	–0.14	0.49	–0.93	–0.21	1.01	1349
a_2	–0.94	0.28	–1.49	–0.94	–0.41	2114
a_3	–0.19	0.17	–0.53	–0.19	0.14	1923
a_4	–0.22	0.25	–0.73	–0.22	0.27	553
a_5	–0.63	0.33	–1.31	–0.63	–0.01	2383
a_6	–0.51	0.51	–1.49	–0.52	0.53	2453
GDP_1	0.75	0.96	–1.02	0.67	2.96	4363
GDP_2	0.48	0.24	–0.01	0.49	0.94	3739
GDP_3	0.68	0.12	0.45	0.68	0.91	1275
GDP_4	1.48	0.74	0.36	1.41	3.02	603
GDP_5	0.04	0.75	–1.67	0.17	1.21	2579
GDP_6	0.83	0.85	–0.76	0.76	2.62	2190
PS_1	–0.30	0.30	–0.99	–0.27	0.24	6140
PS_2	–0.17	0.31	–0.79	–0.18	0.48	2164
PS_3	–0.25	0.14	–0.53	–0.24	0.03	4672
PS_4	–0.15	0.25	–0.62	–0.16	0.38	4930
PS_5	–0.01	0.62	–0.99	–0.10	1.50	3813
PS_6	0.13	0.40	–0.46	0.05	1.05	1105
σ_a	0.61	0.43	0.13	0.50	1.71	1873
σ_{GDP}	0.96	0.86	0.08	0.75	3.13	839
σ_{PS}	0.42	0.47	0.05	0.29	1.46	1282
σ_y	0.52	0.06	0.41	0.52	0.64	1266

Supplementary Table S20 (cond.): Numerical values of estimated parameters in Model 3 using both pathogen stress and GDP per capita as an independent variable and each of three individualism/collectivism scores as a dependent variable. GDP and PS represent GDP per capita and pathogen stress, respectively. Subscript numbers under *a*, GDP, and PS represent global regions (1: Sub-Saharan Africa, 2 East Asia and Pacific, 3: Europe and Central Asia, 4: Latin America and Caribbean, 5: Middle East and North Africa, and 6: South Asia). *ESS* represents effective sample sizes of MCMC simulations. Rows shaded in gray indicate the effects of slopes when 95% Bayesian credible intervals of values did not include zero.

			Quantiles			
	Mean	*SD*	2.5%	50%	97.5%	*ESS*
Collectivism (Gelfand et al., 2004)						
μ_a	0.43	0.28	–0.15	0.43	0.93	2648
μ_{GDP}	–0.59	0.41	–1.23	–0.66	0.37	1391
μ_{PS}	0.07	0.31	–0.60	0.08	0.63	3527
a_1	0.04	0.47	–0.88	0.12	0.78	153
a_2	0.57	0.22	0.15	0.56	1.00	311
a_3	0.51	0.18	0.20	0.52	0.87	263
a_4	0.33	0.25	–0.17	0.33	0.82	396
a_5	0.62	0.21	0.22	0.61	1.06	3855
a_6	0.44	0.46	–0.56	0.45	1.44	1567
GDP_1	–0.25	0.77	–1.31	–0.44	1.64	1183
GDP_2	–0.64	0.22	–1.07	–0.65	–0.19	1766
GDP_3	–0.90	0.15	–1.20	–0.90	–0.61	3298
GDP_4	–0.45	0.57	–1.35	–0.56	0.87	1566
GDP_5	–0.70	0.32	–1.33	–0.73	–0.02	3001
GDP_6	–0.62	0.68	–1.87	–0.71	0.91	2782
PS_1	0.32	0.32	–0.28	0.29	1.01	4551
PS_2	–0.02	0.32	–0.69	–0.01	0.58	455
PS_3	0.41	0.16	0.11	0.41	0.73	730
PS_4	–0.09	0.35	–0.84	–0.08	0.52	4572
PS_5	–0.27	0.40	–1.10	–0.26	0.42	3412
PS_6	0.10	0.46	–0.84	0.11	1.04	1542
σ_a	0.41	0.37	0.05	0.33	1.31	1729
σ_{GDP}	0.56	0.55	0.03	0.41	1.95	431
σ_{PS}	0.51	0.39	0.08	0.42	1.54	893
σ_y	0.53	0.06	0.43	0.53	0.67	279

Supplementary Tables S21: Summary of analysis results using both pathogen stress and GDP per capita as independent variables and each of three individualism/collectivism scores as a dependent variable. Parameters in parentheses indicate the parameters in Model 3. Asterisks in columns of global effects (GDP, PS, μ_{GE}, or μ_{PS}) indicate that the effect was significant (i.e., the 95% Bayesian credible interval of the effect did not include zero). Numbers in columns of region–specific effects (GDP_j or PS_j) indicate global regions in which significant effects were found (1 = Sub-Saharan Africa, 2 = East Asia and Pacific, 3 = Europe and Central Asia, 4 = Latin America and Caribbean, 5 = Middle East and North Africa, and 6 = South Asia). Rows shaded in gray indicate the best model, in which the WAIC value was smallest in three models.

Dependent variables	Model	Parameters			
		GDP (μ_{GDP})	PS (μ_{PS})	(GDP_j)	(PS_j)
Individualism	Model 1	*	*		
(Hofstede, 2001)	Model 2	*	*		
	Model 3			3	3
Individualism	Model 1	*	*		
(Suh et al., 1998)	Model 2	*			
	Model 3			3, 4	
Collectivism	Model 1	*	*		
(Gelfand et al., 2004)	Model 2	*	*		
	Model 3			2, 3, 5	3

Supplementary References

Fincher, C., Thornhill, R., Murray, D. R., and Schaller, M. (2008). Pathogen prevalence predicts human cross-cultural variability in individualism/collectivism. *Proc. R. Soc. Lond., B, Biol. Sci.* 275, 1279–1285. doi: 10.1098/rspb.2008.0094

Gelfand, M. J., Bhawuk, D. P. S., Nishii, L. H. and Bechtold, D. J. (2004). Individualism and collectivism. In *Culture, leadership, and organizations: the GLOBE study of 62 societies* (eds R. J. House, P. J. Hanges, M. Javidan, P. W. Dorfman & V. Gupta), pp. 437–512. Thousand Oaks, CA: Sage Publications.

Hofstede, G. H. (2001). *Culture's Consequences: Comparing Values, Behaviors, Institutions, and Organizations across Nations*. Thousand Oaks, CA: Sage Publications.

Kashima, E. S. and Kashima, Y. (1998). Culture and language: the case of cultural dimensions and personal pronoun use. *J. Cross Cult. Psychol.* 29, 461–486. doi:10.1177/ 0022022198293005

Murray, D. R., and Schaller, M. (2010). Historical prevalence of infectious diseases within 230 geopolitical regions: A tool for investigating origins of culture. *J. Cross Cult. Psychol.* 41, 99–108. doi: 10.1177/0022022109349510

Suh, E., Diener, E., Oishi, S. and Triandis, H. C. (1998). The shifting basis of life satisfaction judgments across cultures: emotions versus norms. *J. Pers. Soc. Psychol.* 74, 482–493. doi:10.1037/0022-3514.74.2.482

Thornhill, R., Fincher, C. L., Murray, D. R., and Schaller, M. (2010). Zoonotic and non-zoonotic diseases in relation to human personality and societal values: Support for the parasite-stress model. *Evol. Psychol.* 8, 151–169.
