# Supplementary material for: Cultural Differences in Strength of Conformity Explained Through Pathogen Stress: A Statistical Test Using Hierarchical Bayesian Estimation
Source: Front Psychol. 2018 Oct 11;9:1921. doi: 10.3389/fpsyg.2018.01921 (PMC6193438; doi:10.3389/fpsyg.2018.01921)

Trace plots

Trace plots

Here, we report trace plots of parameter values by each model and dependent variable. Each graph shows transitions of parameter values by iterations of the MCMC simulation. The transitions were shown by each of four MCMC chains. Horizontal and vertical axis represent iterations and parameter values, respectively. Labels in the top of each graph indicate each parameter. Burn-in steps (i.e., first 1,000 iterations, which were discarded when estimating posterior distributions of each parameter) were shaded in gray.

Results of each model when government effectiveness was used as an independent variable

Model 1

Labels in the top of each graph represent types of parmeter: a_0 = a_0, GE = GE, PS = PS, and sigma_y = σ_y.

Individualism

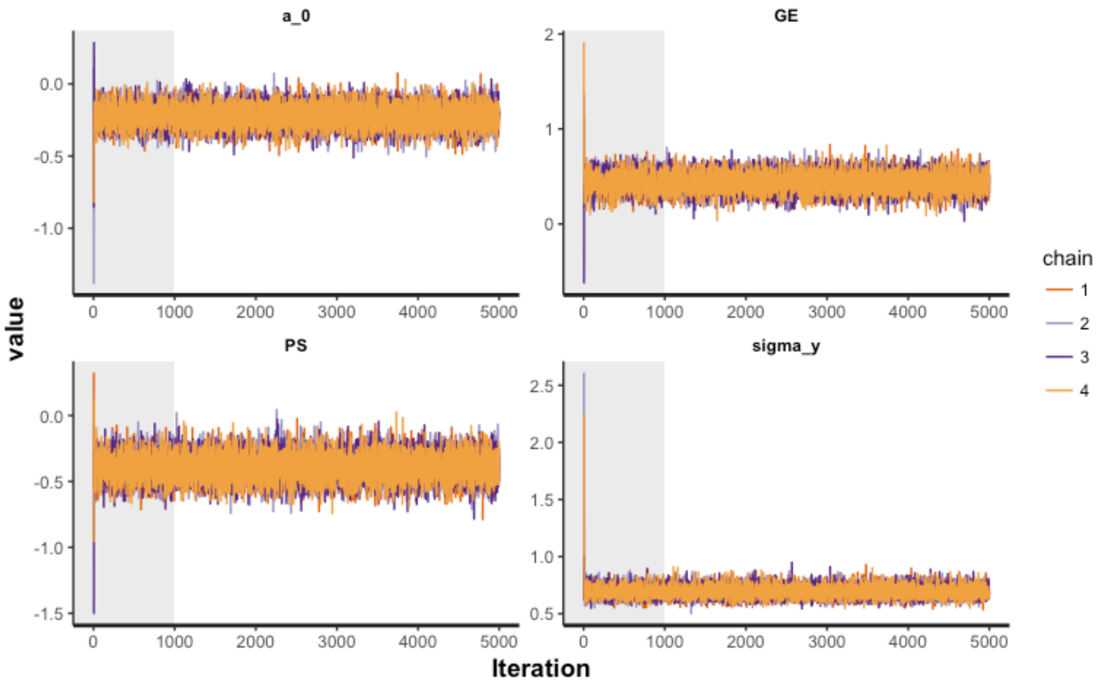

Conformity 1

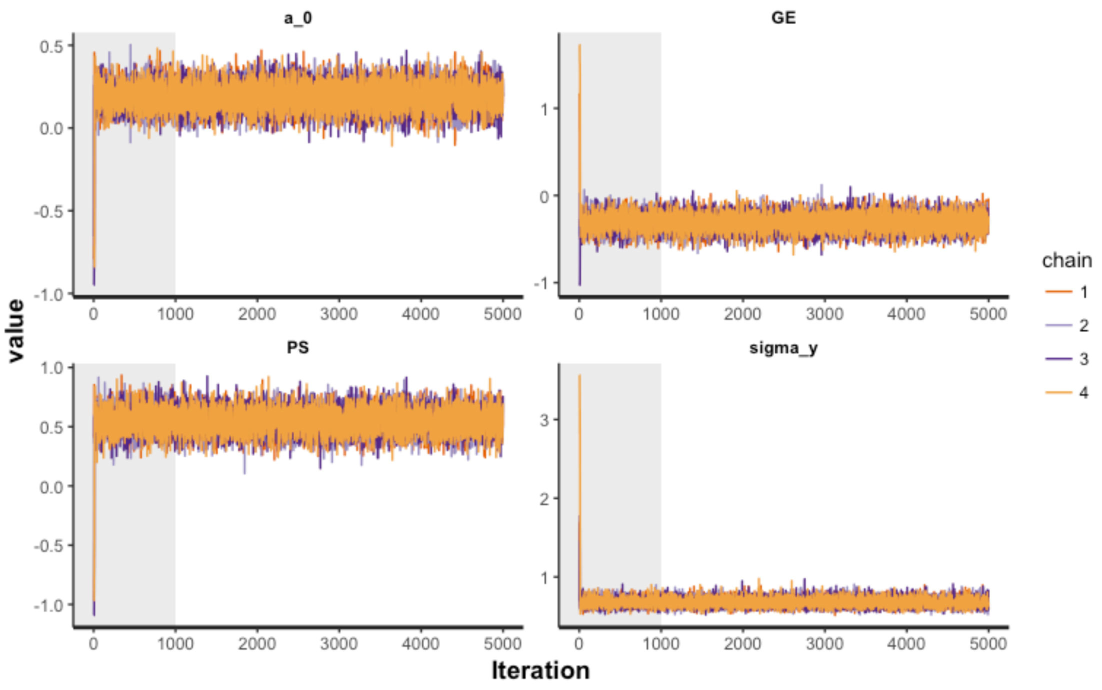

Conformity 2

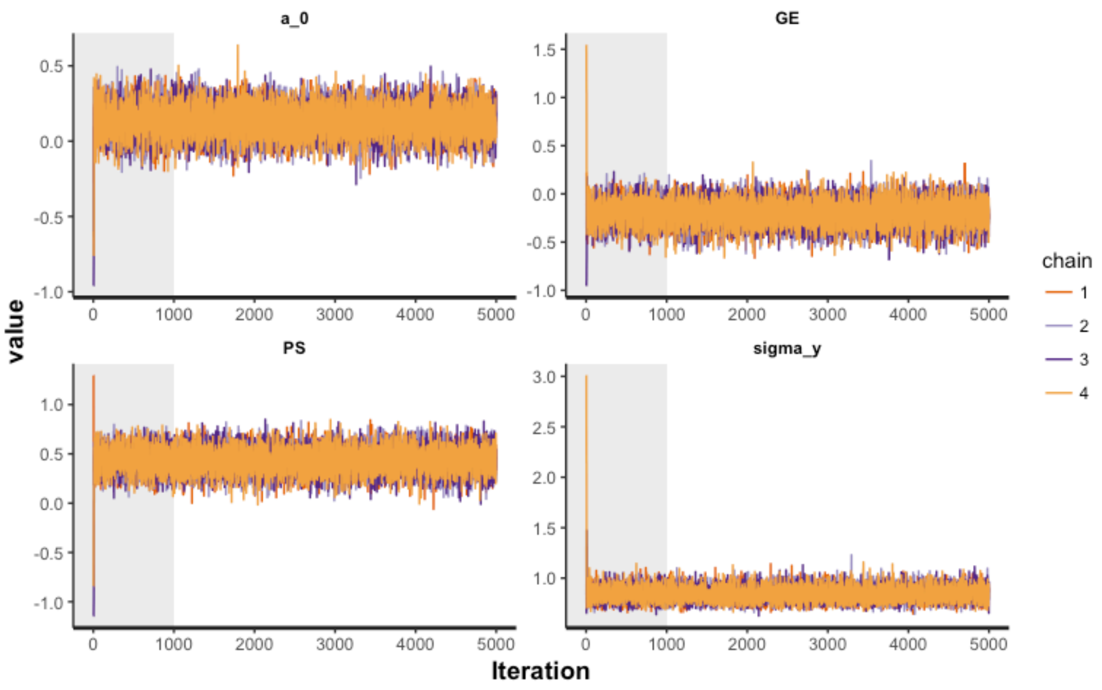

Conformity 3

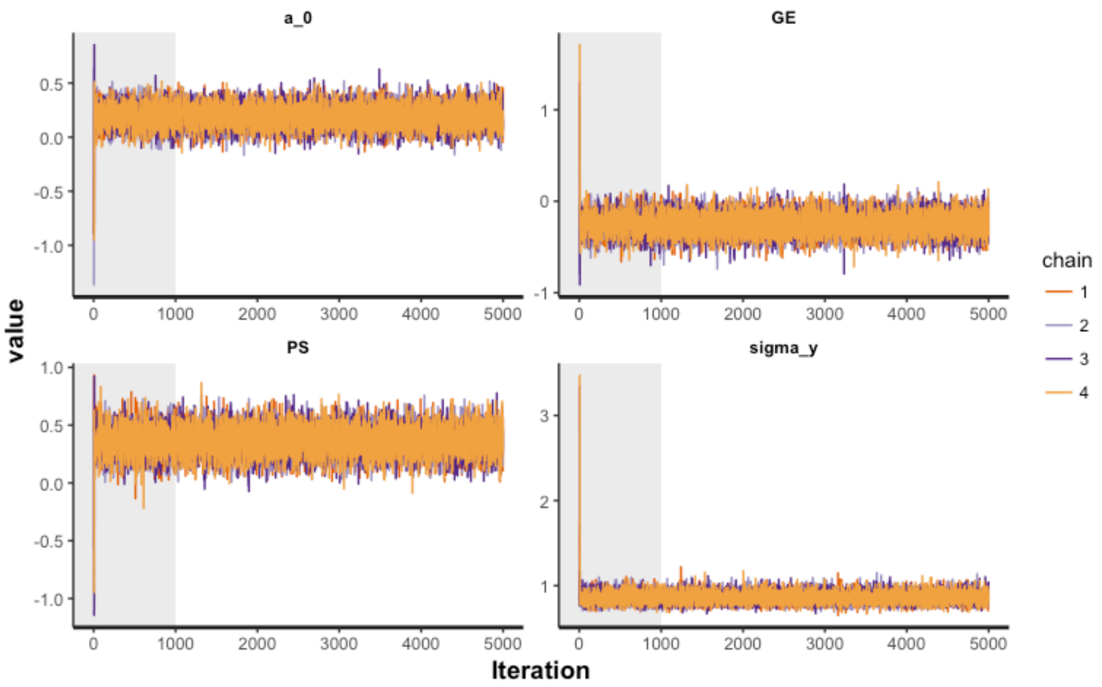

Conformity 4

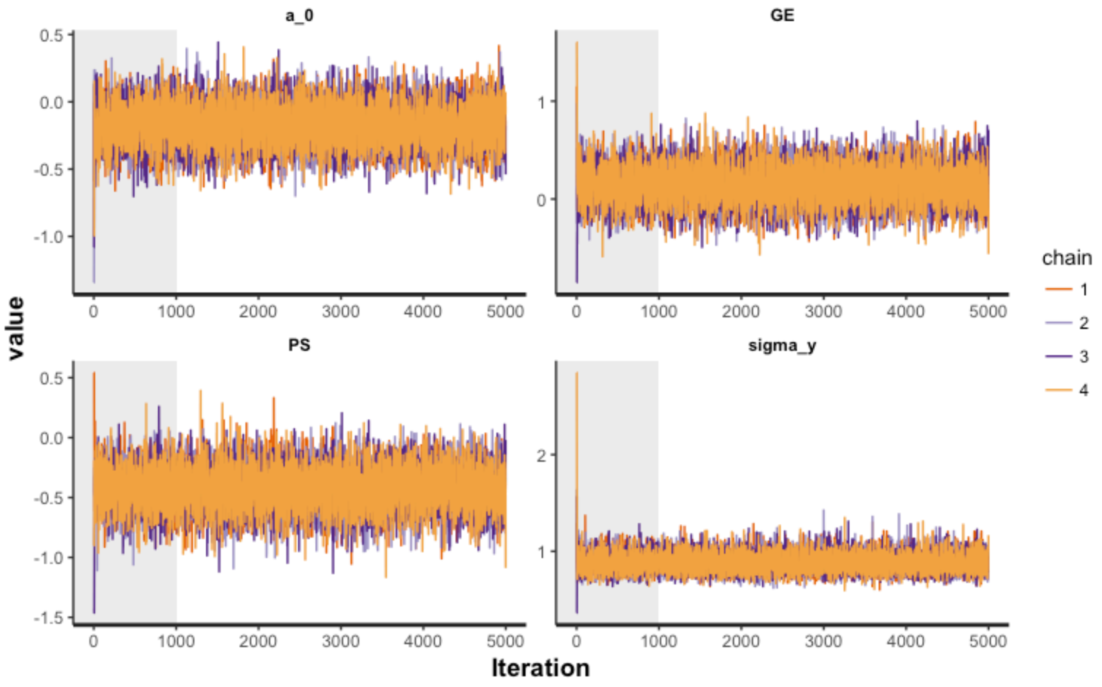

Model 2

Labels in the top of each graph represent types of parmeter: mu_a = μ_a, GE = GE, PS = PS, a[j] = a_j, sigma_a = σ_a, sigma_y = σ_y. Subscript numbers under a represent global regions (1: Sub-Saharan Africa, 2 East Asia and Pacific, 3: Europe and Central Asia, 4: Latin America and Caribbean, 5: Middle East and North Africa, and 6: South Asia).

Individualism

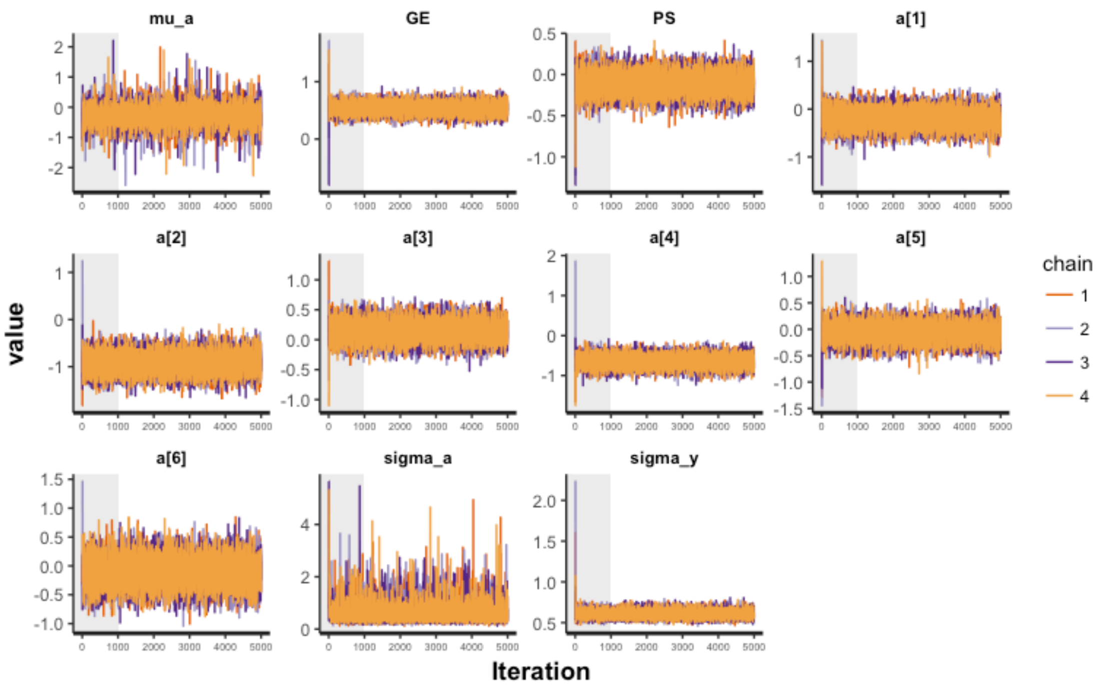

Conformity 1

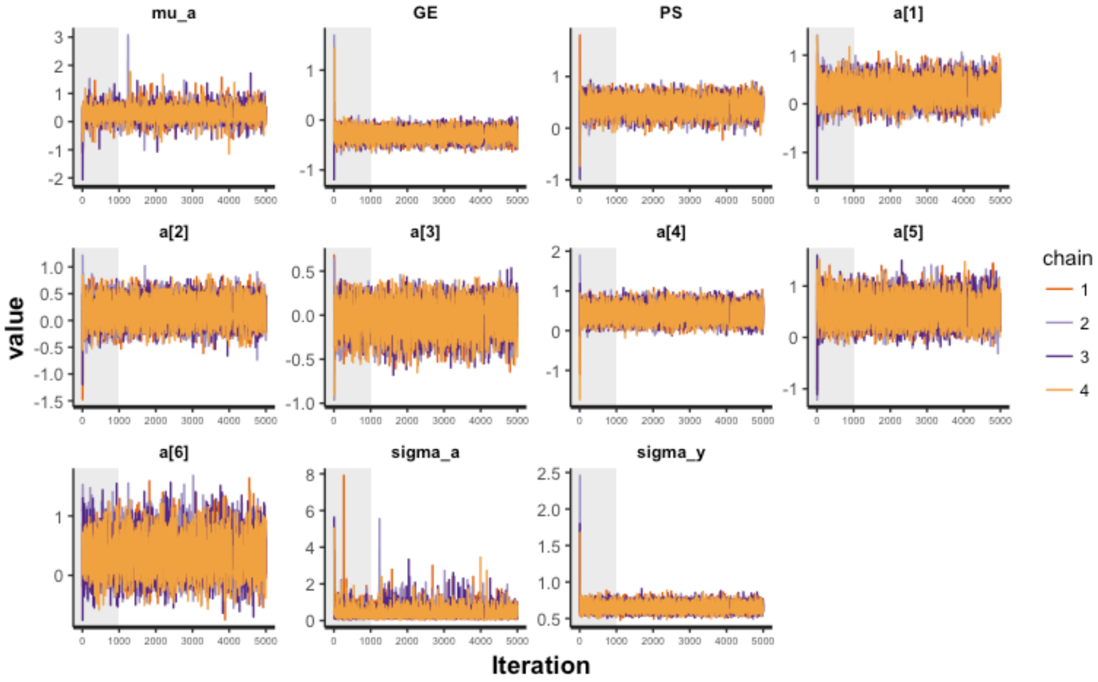

Conformity 2

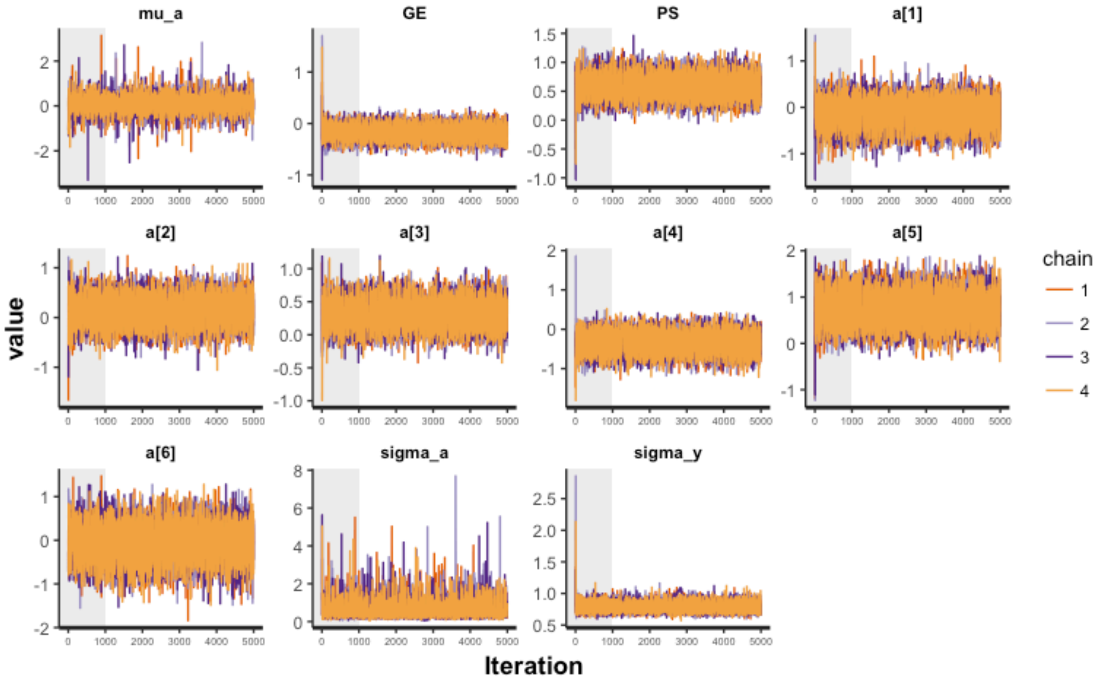

Conformity 3

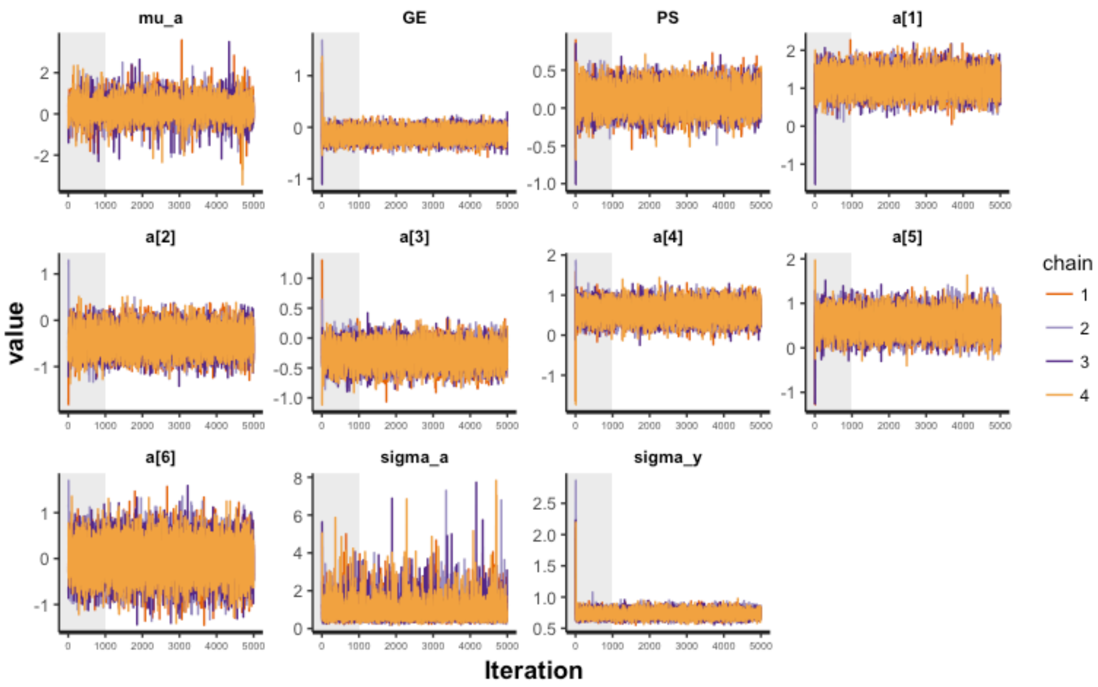

Conformity 4

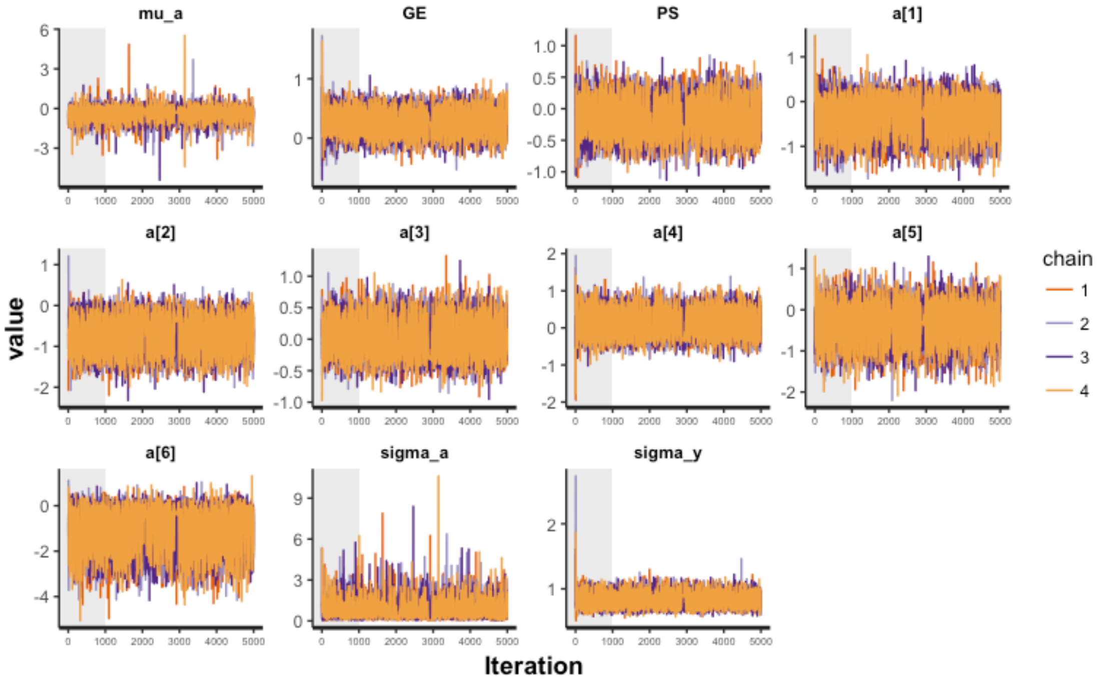

Model 3

Labels in the top of each graph represent types of parmeter: mu_a = μ_a, mu_GE = μ_{GE}, mu_PS = μ_{PS}, a[j] = a_j, GE[j] = GE_j, PS[j] = PS_j, sigma_a = σ_a, sigma_GE = σ_{GE}, sigma_PS = σ_{PS}, and sigma_y = σ_y. Subscript numbers under a, GE, and PS represent global regions (1: Sub-Saharan Africa, 2 East Asia and Pacific, 3: Europe and Central Asia, 4: Latin America and Caribbean, 5: Middle East and North Africa, and 6: South Asia).

Individualism

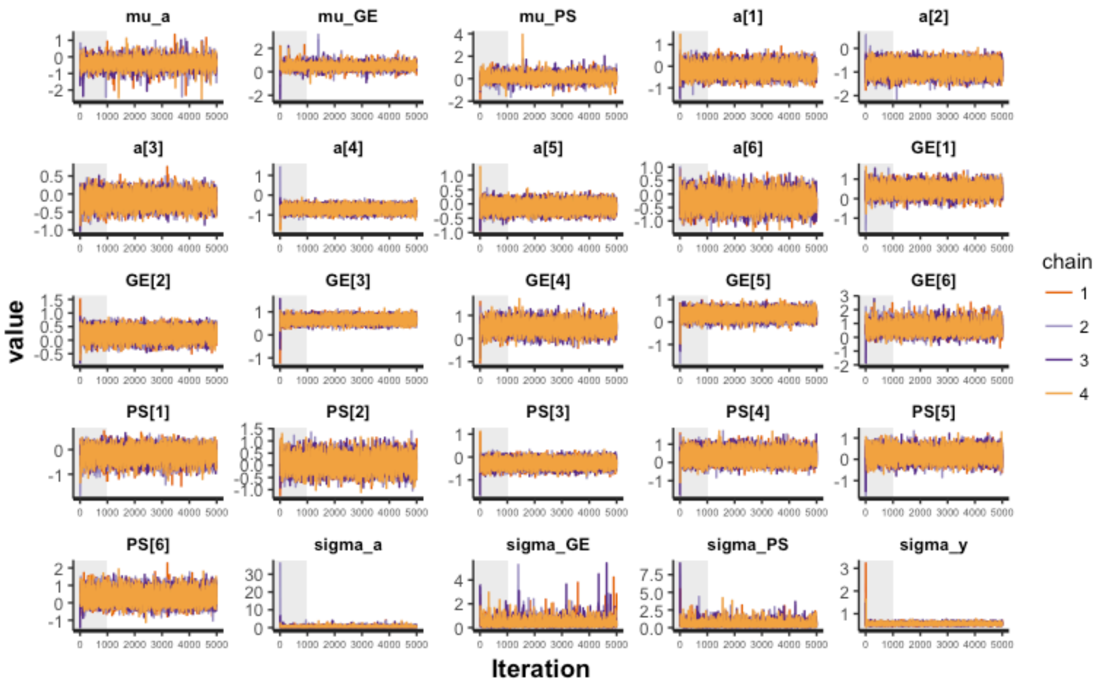

Conformity 1

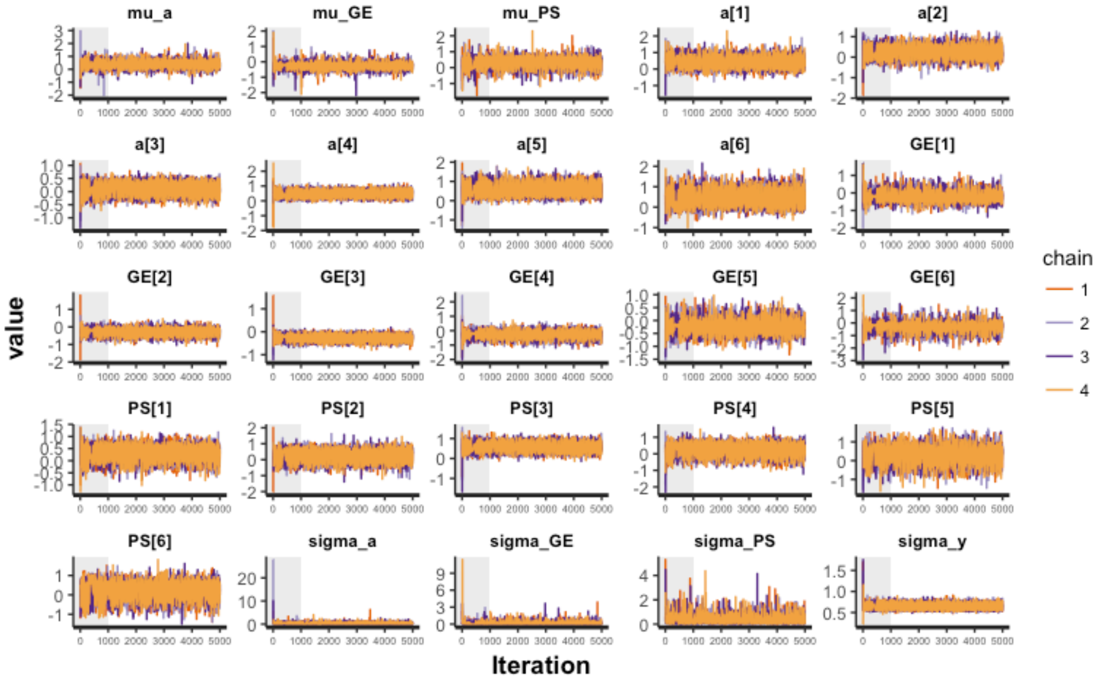

Conformity 2

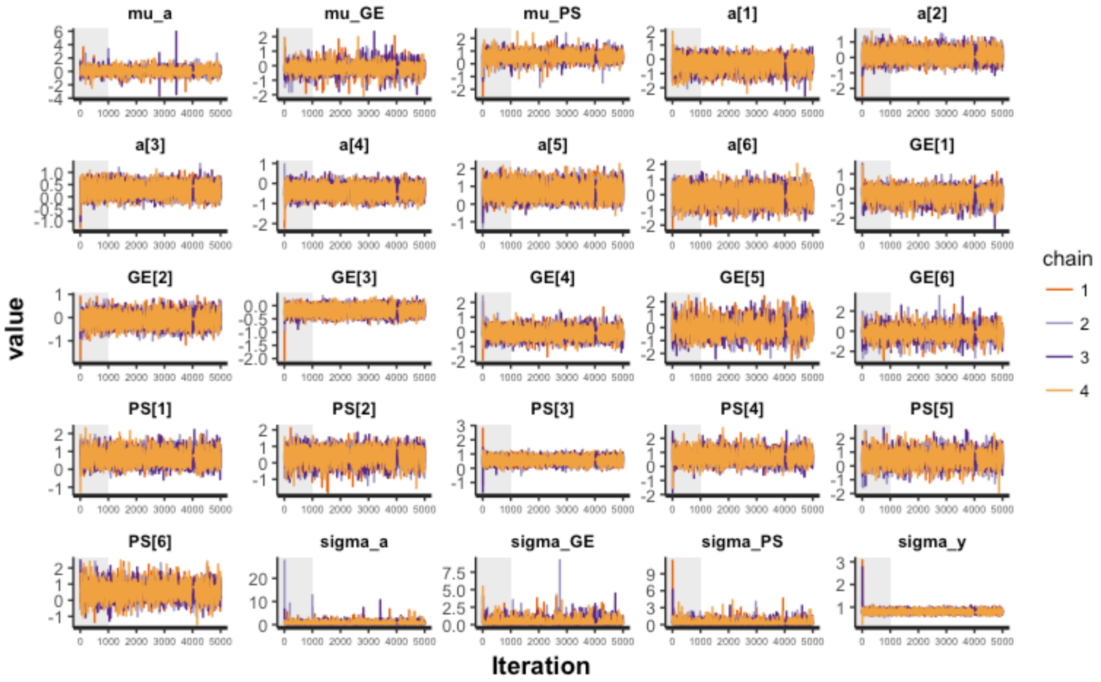

Conformity 3

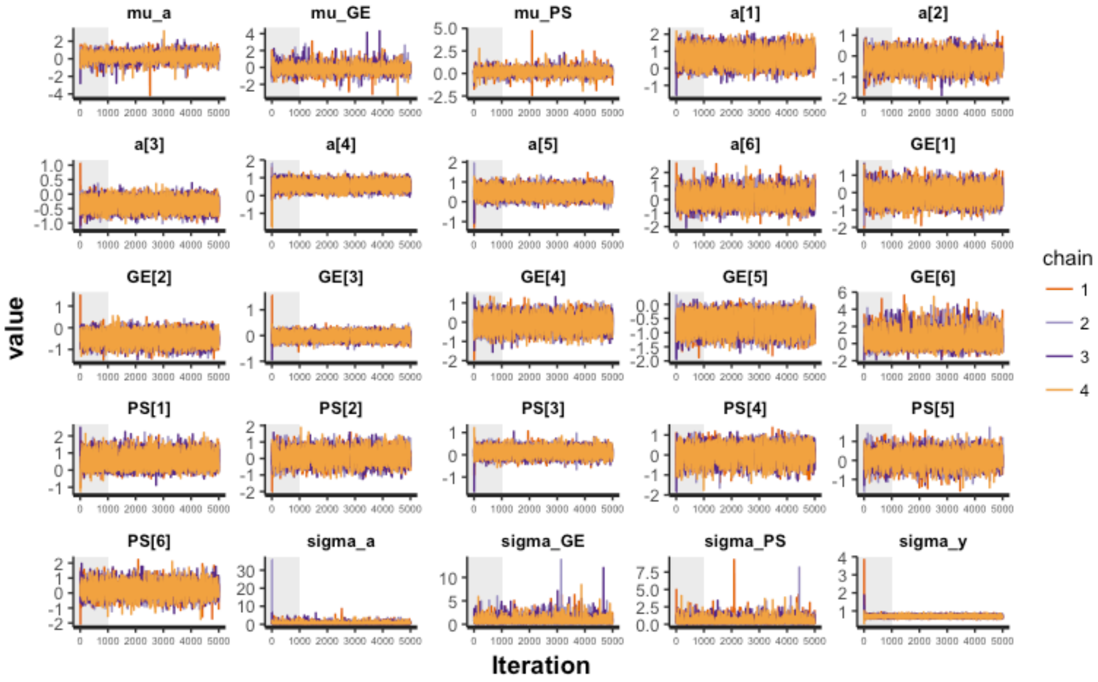

Conformity 4

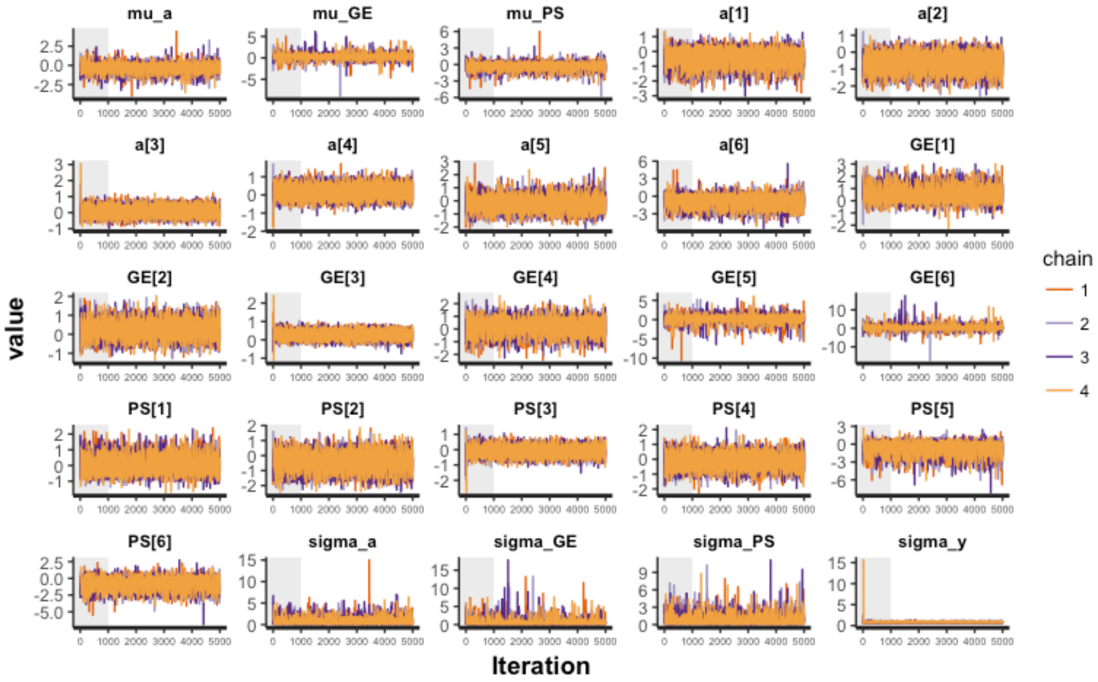

Results of each model when GDP per capita was used as an independent variable

Model 1

Labels in the top of each graph represent types of parmeter: a_0 = a_0, GDP = GDP, PS = PS, sigma_y = σ_y.

Individualism

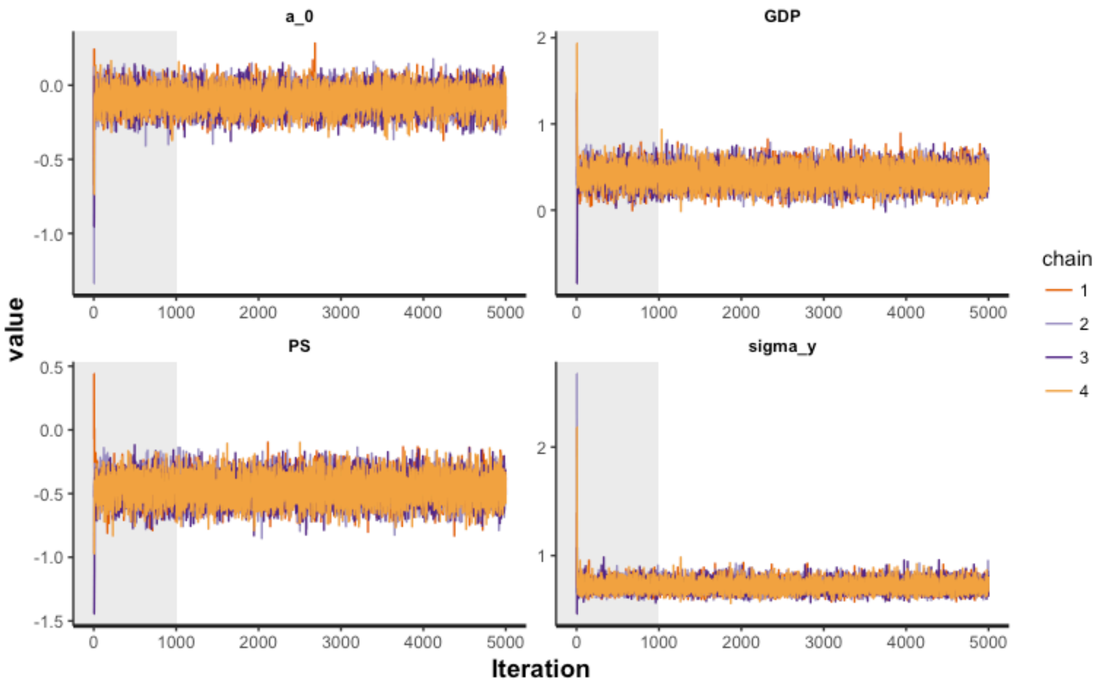

Conformity 1

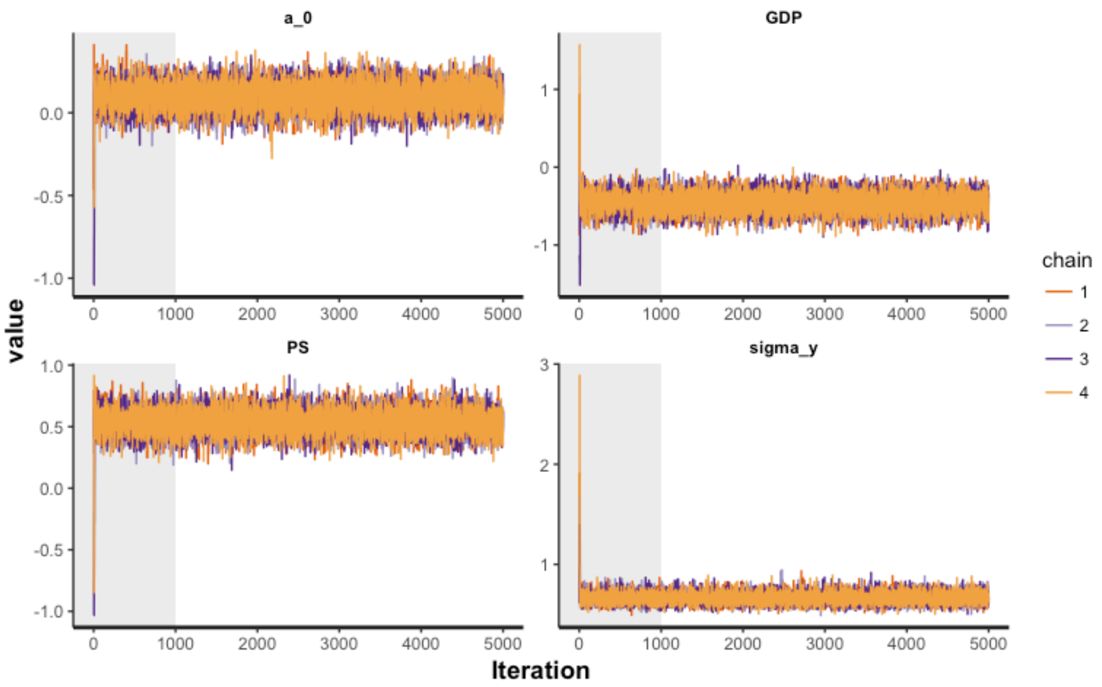

Conformity 2

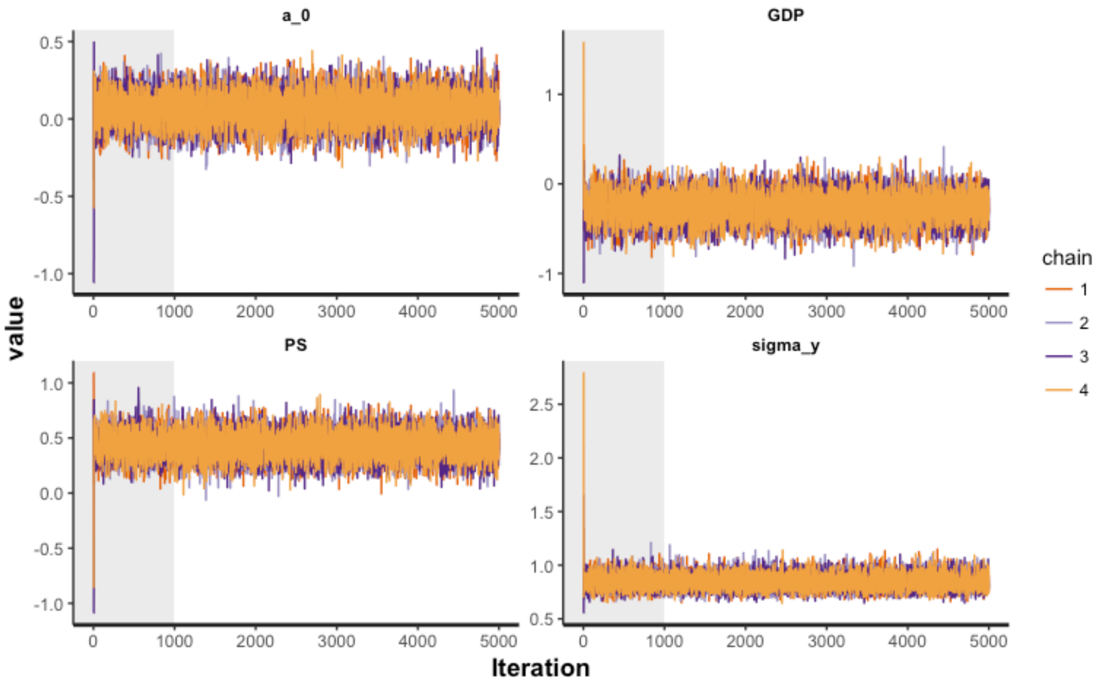

Conformity 3

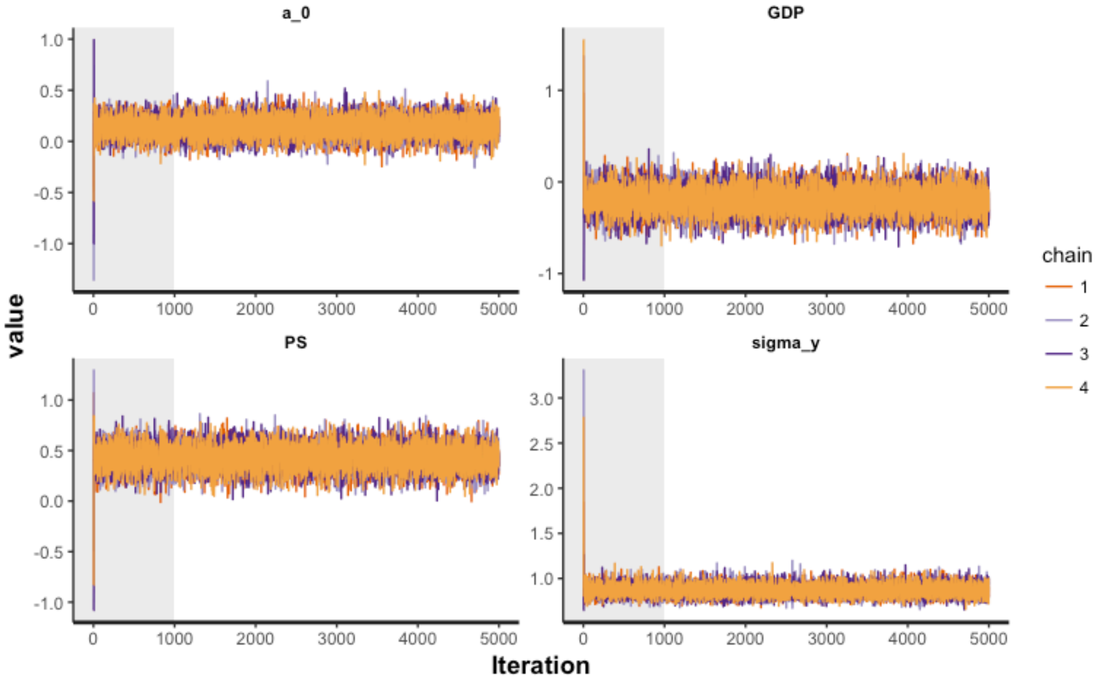

Conformity 4

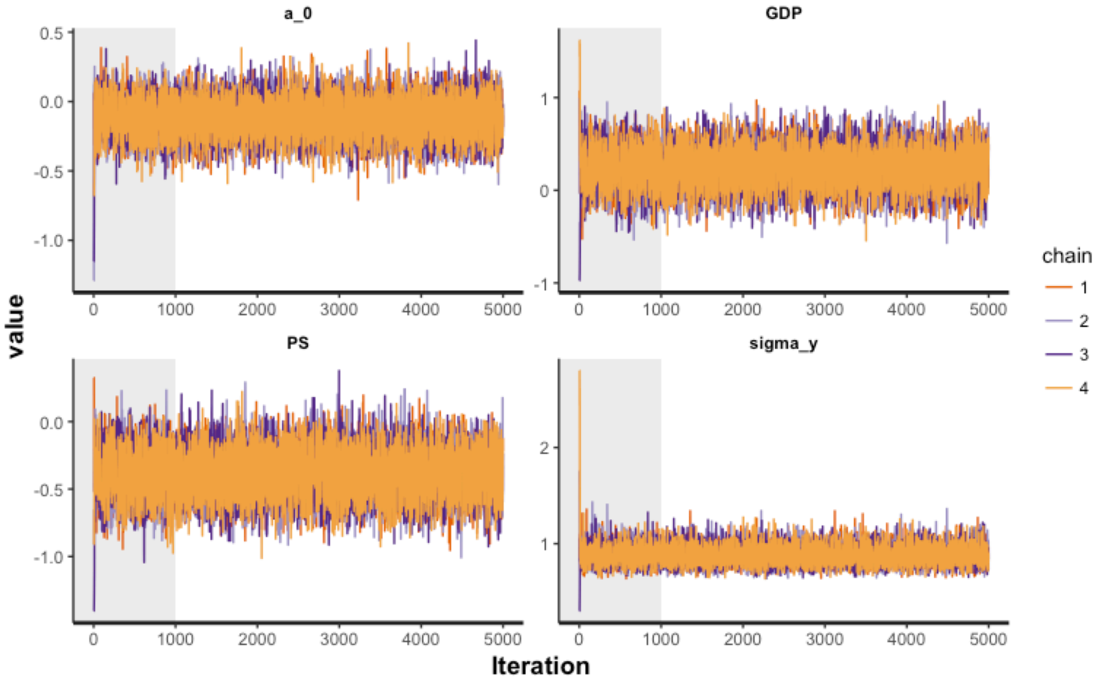

Model 2

Labels in the top of each graph represent types of parmeter: mu_a = μ_a, GDP = GDP, PS = PS, a[j] = α_j, sigma_a = σ_a, sigma_y = σ_y. Subscript numbers under α represent global regions (1: Sub-Saharan Africa, 2 East Asia and Pacific, 3: Europe and Central Asia, 4: Latin America and Caribbean, 5: Middle East and North Africa, and 6: South Asia).

Individualism

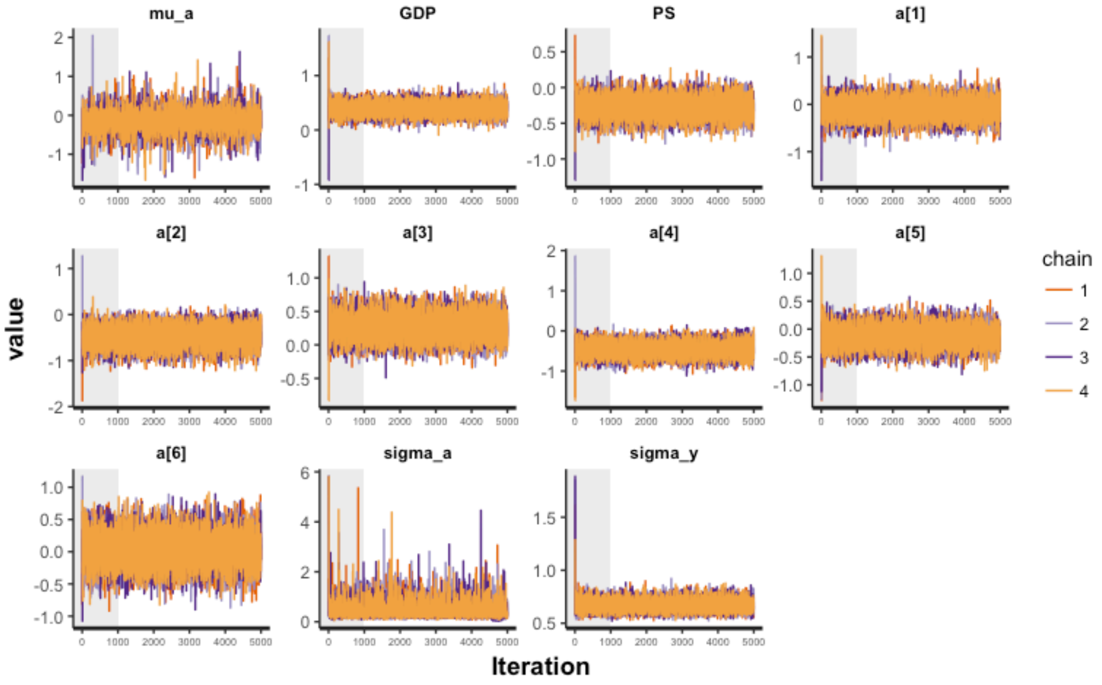

Conformity 1

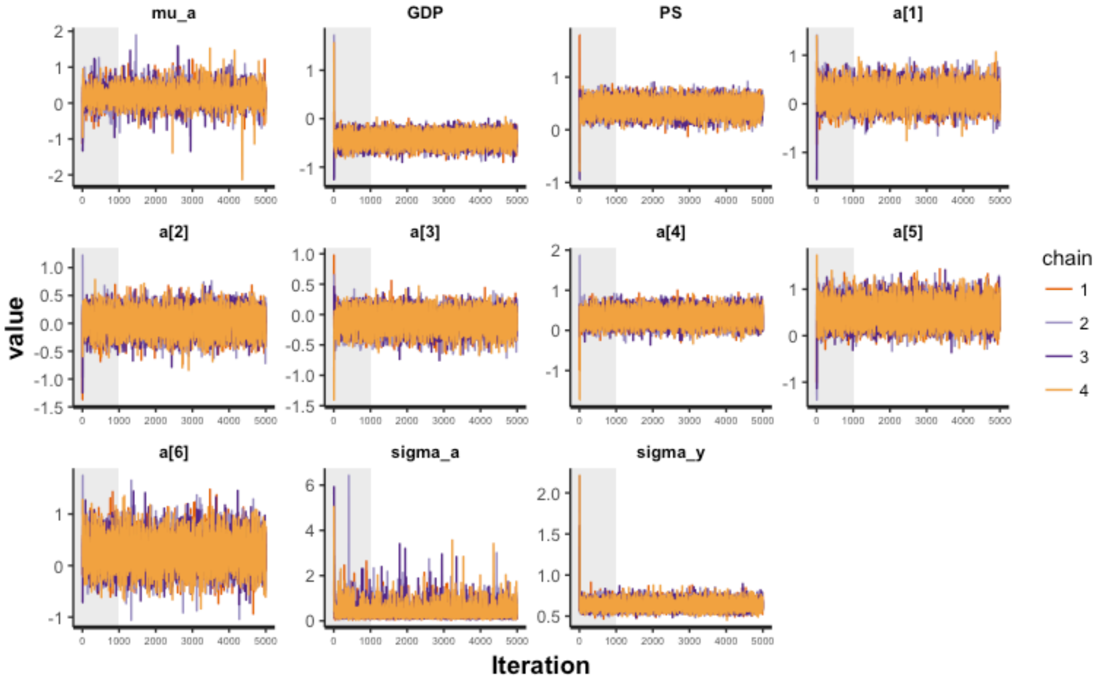

Conformity 2

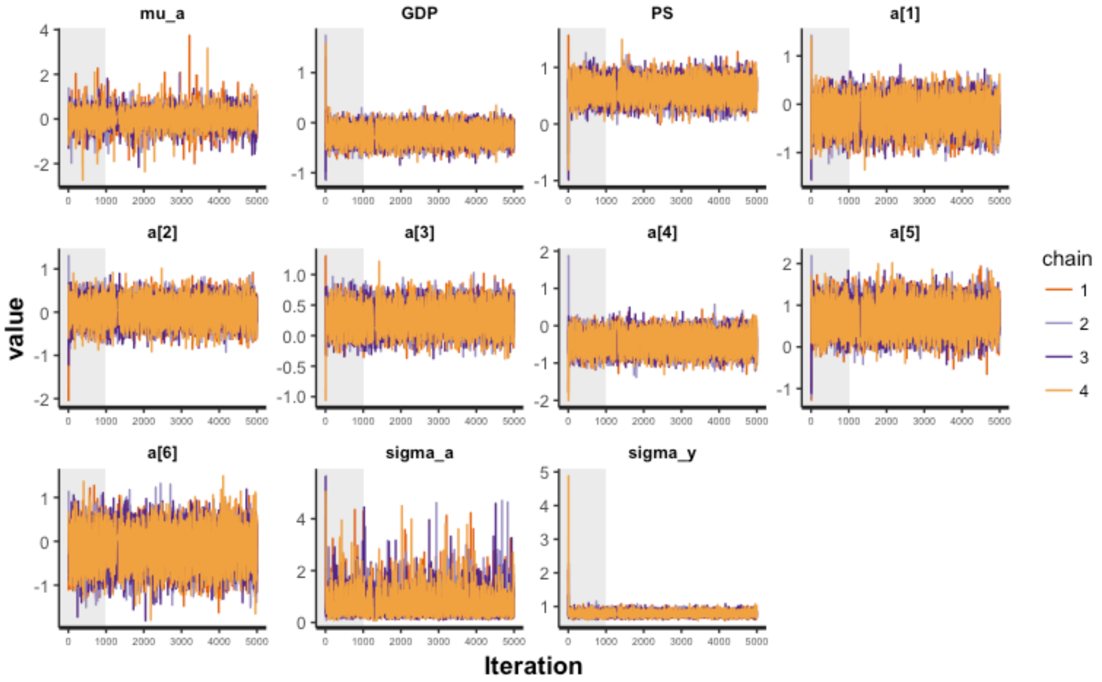

Conformity 3

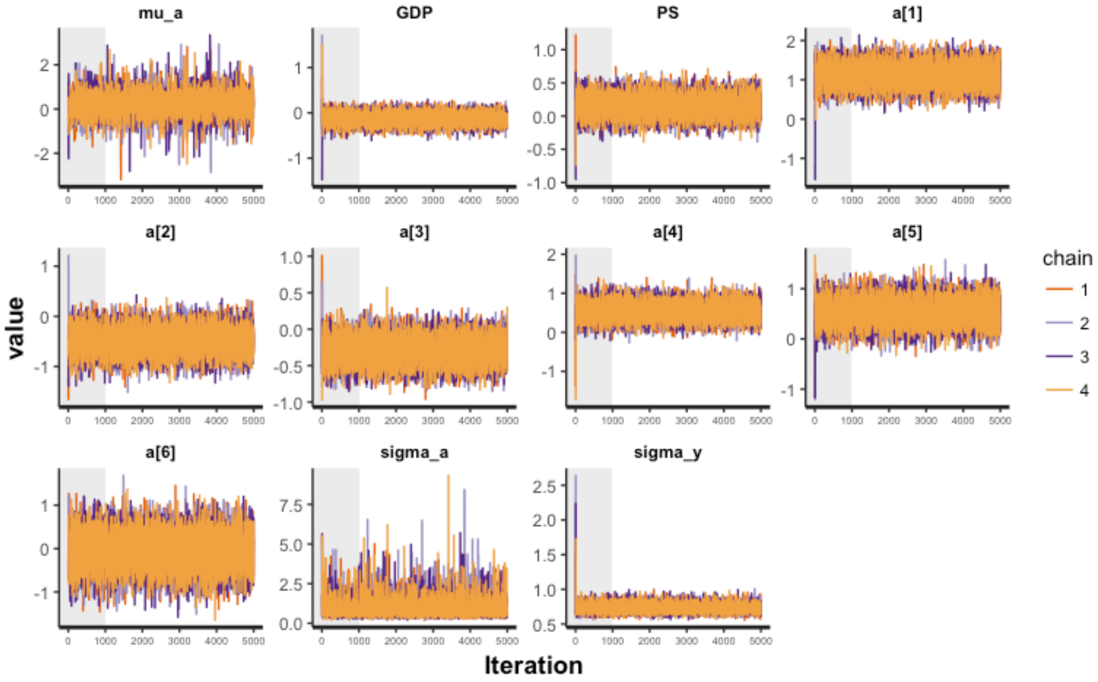

Conformity 4

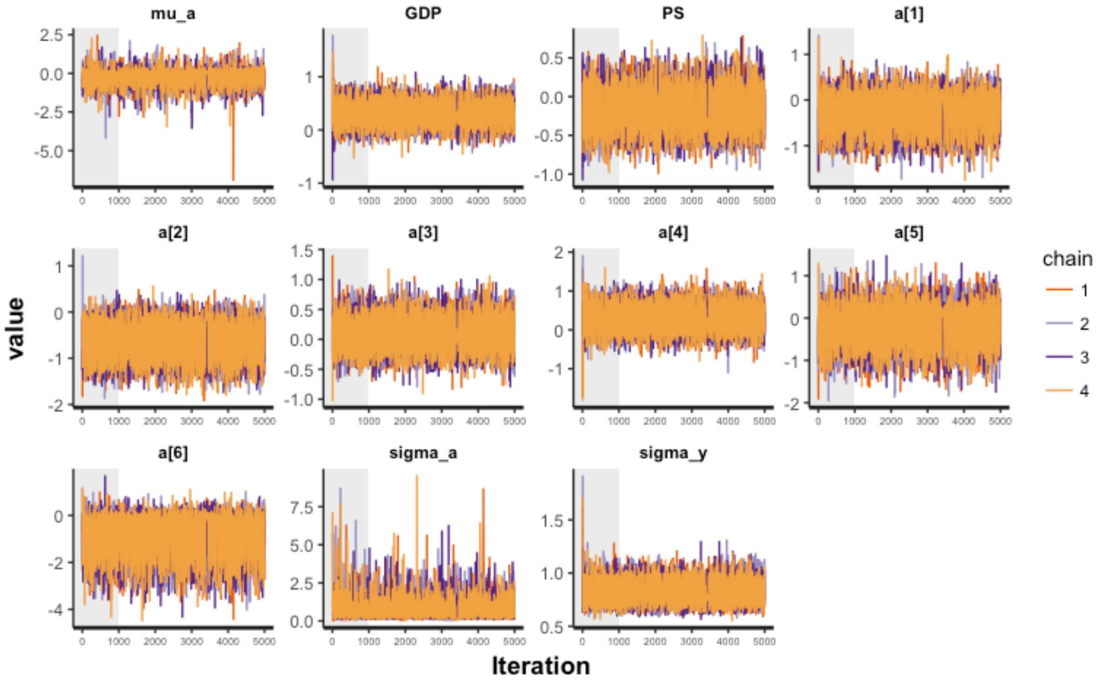

Model 3

Labels in the top of each graph represent types of parmeter: mu_a = μ_a, mu_GDP = μ_{GDP}, mu_PS = μ_{PS}, a[j] = a_j, GDP[j] = GDP_j, PS[j] = PS_j, sigma_a = σ_a, sigma_GDP = σ_{GDP}, sigma_PS = σ_{PS}, and sigma_y = σ_y. Subscript numbers under a, GDP, and PS represent global regions (1: Sub-Saharan Africa, 2 East Asia and Pacific, 3: Europe and Central Asia, 4: Latin America and Caribbean, 5: Middle East and North Africa, and 6: South Asia).

Individualism

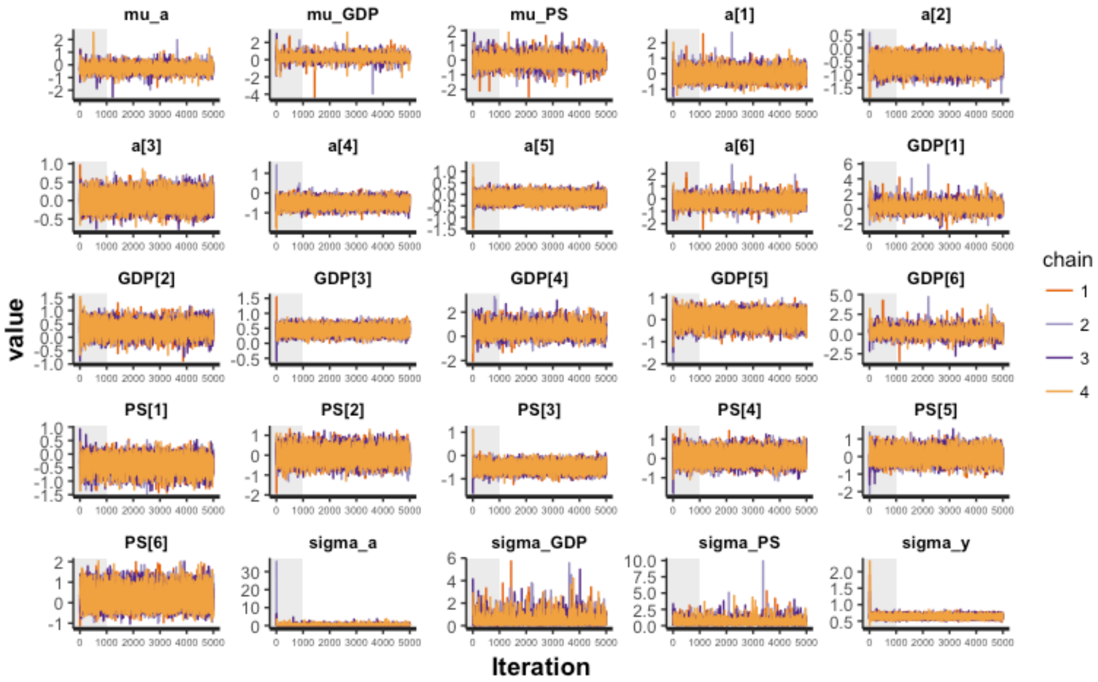

Conformity 1

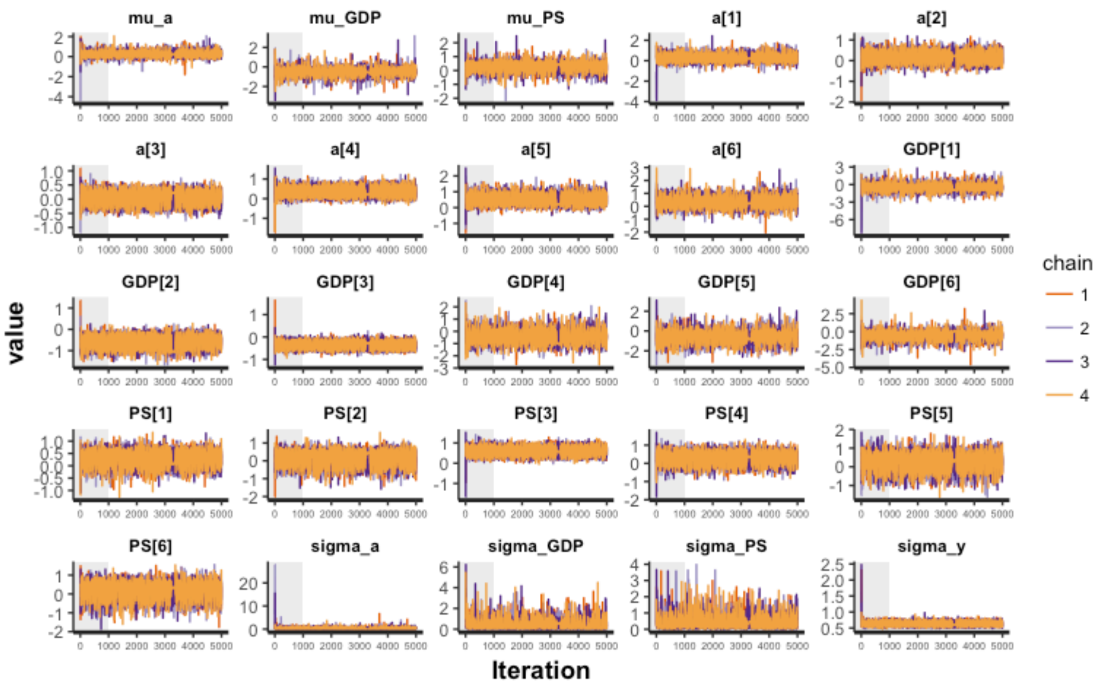

Conformity 2

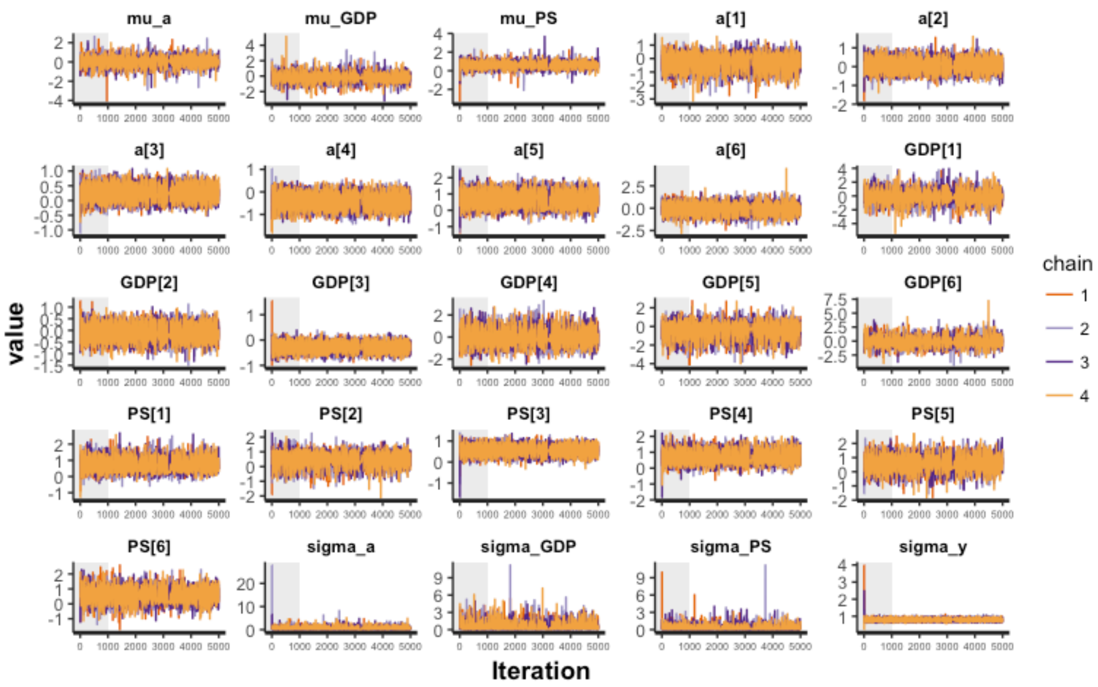

Conformity 3

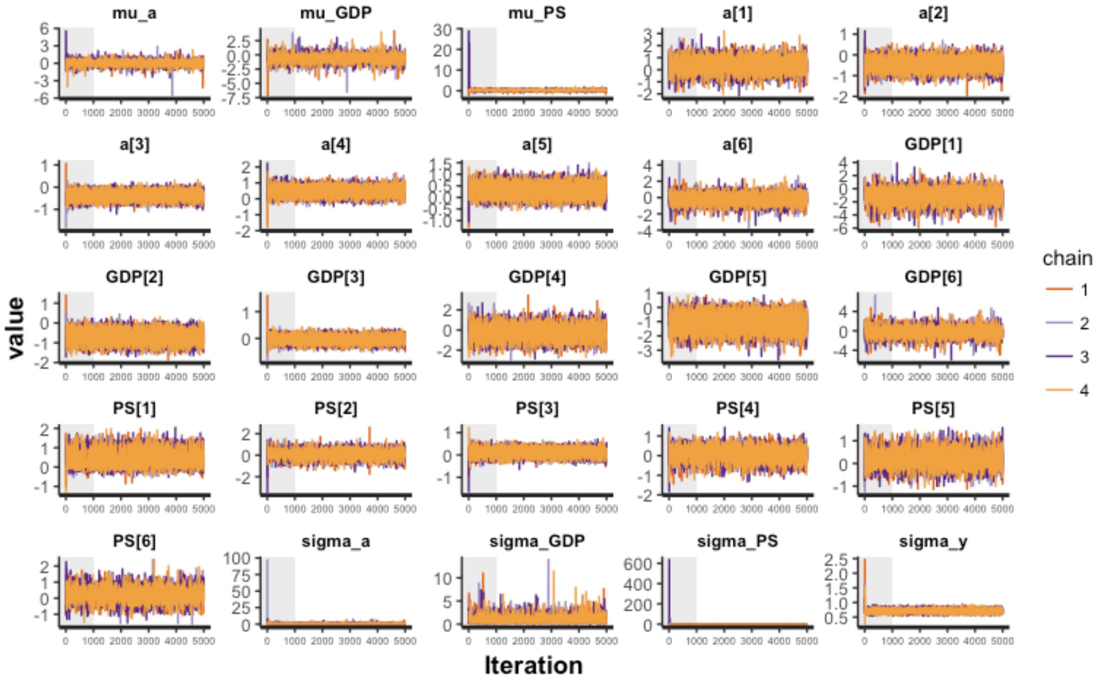

Conformity 4

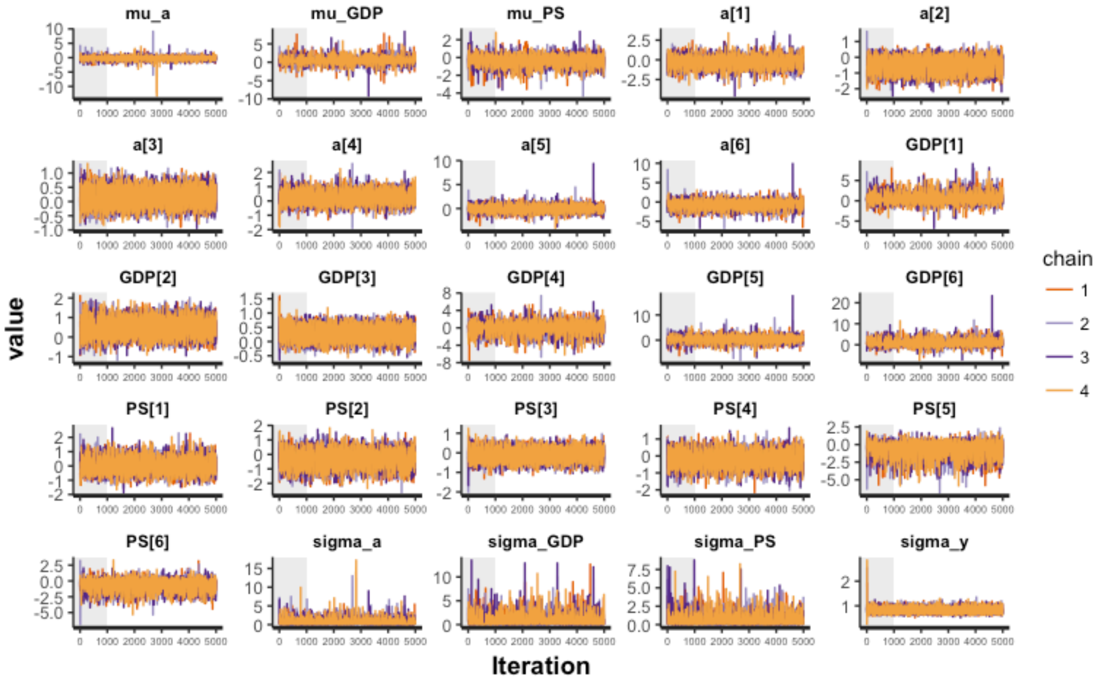

Supplement: Supplementary file 3 [file Data_Sheet_3.PDF]
